# Randomized, placebo controlled phase I trial of safety, pharmacokinetics, pharmacodynamics and acceptability of tenofovir and tenofovir plus levonorgestrel vaginal rings in women

**DOI:** 10.1371/journal.pone.0199778

**Published:** 2018-06-28

**Authors:** Andrea Ries Thurman, Jill L. Schwartz, Vivian Brache, Meredith R. Clark, Timothy McCormick, Neelima Chandra, Mark A. Marzinke, Frank Z. Stanczyk, Charlene S. Dezzutti, Sharon L. Hillier, Betsy C. Herold, Raina Fichorova, Susana N. Asin, Christiane Rollenhagen, Debra Weiner, Patrick Kiser, Gustavo F. Doncel

**Affiliations:** 1 CONRAD, Eastern Virginia Medical School, Arlington, Virginia, United States of America; 2 CONRAD, Eastern Virginia Medical School, Norfolk, Virginia, United States of America; 3 Profamilia, Santo Domingo, Dominican Republic; 4 Johns Hopkins University School of Medicine, Baltimore, Maryland, United States of America; 5 University of Southern California Keck School of Medicine, Los Angeles, California, United States of America; 6 University of Pittsburgh, Department of Obstetrics, Gynecology & Reproductive Sciences, Department of Infectious Diseases & Microbiology, Graduate School of Public Health, Pittsburgh, Pennsylvania, United States of America; 7 University of Pittsburgh School of Medicine, Departments of Obstetrics, Gynecology and Reproductive Sciences and Microbiology and Molecular Genetics, Pittsburgh, Pennsylvania, United States of America; 8 Albert Einstein College of Medicine, Bronx, New York, United States of America; 9 Laboratory of Genital Tract Biology, Brigham and Women’s Hospital and Harvard Medical School, Boston, Massachusetts, United States of America; 10 V.A. Medical Center, White River Junction, VT and Geisel School of Medicine at Dartmouth, New Hampshire; 11 FHI360, Durham, North Carolina, United States of America; 12 Northwestern University, Evanston, Illinois, United States of America; Imperial College London, UNITED KINGDOM

## Abstract

To prevent the global health burdens of human immunodeficiency virus [HIV] and unintended/mistimed pregnancies, we developed an intravaginal ring [IVR] that delivers tenofovir [TFV] at ~10mg/day alone or with levonorgestrel [LNG] at ~20μg/day for 90 days. We present safety, pharmacokinetics, pharmacodynamics, acceptability and drug release data in healthy women. CONRAD A13-128 was a randomized, placebo controlled phase I study. We screened 86 women; 51 were randomized to TFV, TFV/LNG or placebo IVR [2:2:1] and 50 completed all visits, using the IVR for approximately 15 days. We assessed safety by adverse events, colposcopy, vaginal microbiota, epithelial integrity, mucosal histology and immune cell numbers and phenotype, cervicovaginal [CV] cytokines and antimicrobial proteins and changes in systemic laboratory measurements, and LNG and TFV pharmacokinetics in multiple compartments. TFV pharmacodynamic activity was measured by evaluating CV fluid [CVF] and tissue for antiviral activity using in vitro models. LNG pharmacodynamic assessments were timed based on peak urinary luteinizing hormone levels. All IVRs were safe with no significant colposcopic, mucosal, immune and microbiota changes and were acceptable. Among TFV containing IVR users, median and mean CV aspirate TFV concentrations remained above 100,000 ng/mL 4 hours post IVR insertion and mean TFV-diphosphate [DP] concentrations in vaginal tissue remained above 1,000 fmol/mg even 3 days post IVR removal. CVF of women using TFV-containing IVRs completely inhibited [94–100%] HIV infection in vitro. TFV/LNG IVR users had mean serum LNG concentrations exceeding 300 pg/mL within 1 hour, remaining high throughout IVR use. All LNG IVR users had a cervical mucus Insler score <10 and the majority [95%] were anovulatory or had abnormal cervical mucus sperm penetration. Estimated in vivo TFV and LNG release rates were within expected ranges. All IVRs were safe with the active ones delivering sustained high concentrations of TFV locally. LNG caused changes in cervical mucus, sperm penetration, and ovulation compatible with contraceptive efficacy. The TFV and TFV/LNG rings are ready for expanded 90 day clinical testing.

**Trial registration** ClinicalTrials.gov #NCT02235662

## Introduction

Over 35 million people worldwide are infected with human immunodeficiency virus type 1 [HIV-1] [1]. Women increasingly bear the burden of the HIV-1 pandemic, with more than 50% of new infections occurring in women in sub-Saharan Africa [1]. Herpes simplex virus type 2 [HSV-2] is one of the most prevalent sexually transmitted infections [STIs] worldwide and is linked to an increased risk of HIV-1 acquisition and transmission [2, 3]. Almost half of all pregnancies worldwide, estimated to be over 100 million annually, are unintended [4–6]. Despite the existence of a variety of effective contraceptives, discontinuation and non-use remain high, primarily due to side effects, cost, inconvenient dosing schedules, limited access to prescription products and/or poor acceptance of the method by male partners. Highly effective contraceptives [e.g., sterilization, intrauterine devices, hormonal contraception] typically provide little or no protection against STIs, while barrier methods [e.g., male or female condoms] have unacceptably high contraceptive failure rates with typical use. Poverty, malnutrition, lack of education, and gender inequality fuel the global burdens of unplanned pregnancies, and HSV-2 and HIV-1 acquisition. Products that offer protection against multiple STIs [e.g. HSV-2 and HIV-1] or STIs and unintended pregnancy, termed multi-purpose prevention technologies [MPTs], are urgently needed to reduce these global health burdens.

The first randomized, double blind, placebo-controlled trial of tenofovir [TFV] 1% vaginal gel found a 39% overall reduction in HIV-1 incidence and a 54% reduction among women with high adherence to gel use, as well as a 51% reduction in HSV-2 incidence [7, 8]. However, follow-on studies of TFV vaginal gel found no preventative effect on HIV-1 incidence, likely due to poor adherence to either a daily [9] or a peri-coital dosing regimen [10], particularly among young women. The presence of TFV in plasma of TFV gel users was associated with a significant reduction in HSV-2 incidence in the VOICE study [11]. Recently, two phase III HIV-1 prevention trials using the dapivirine [DPV] intravaginal ring [IVR] reported a significant reduction in HIV-1 incidence among women randomized to the active arm [12, 13]. While these recent IVR trials are a landmark achievement for HIV-1 prevention, when participants were stratified by age, young women, less than 25 years old, showed significantly reduced adherence to IVR use, and women under 21 years old achieved no protective benefit from the DPV IVR [12, 13]. Thus there is a clear need to develop products with added value particularly for young women. It is possible that an MPT may be less stigmatizing than an HIV-1 microbicide or pre-exposure prophylaxis [PrEP] product and adherence may be augmented [14, 15].

CONRAD, in collaboration with the University of Utah and Northwestern University [Kiser Laboratory], developed two MPT IVRs which release TFV alone [potential efficacy against HIV-1 and HSV-2] and TFV in combination with levonorgestrel [LNG] [potential efficacy against HIV-1, HSV-2, and pregnancy] for at least 90 days [16, 17]. The study described herein is a phase I, first in women trial evaluating genital and systemic safety [primary objective], pharmacokinetics [PK] of TFV and LNG [secondary objective], pharmacodynamics [PD] of TFV, surrogates of contraceptive efficacy and acceptability [tertiary objectives] of these IVRs in healthy, HIV-1 uninfected women not at risk of pregnancy.

## Materials and methods

### Clinical study

CONRAD A13-128 was an outpatient, randomized, partially blinded, placebo-controlled, parallel study conducted at the CONRAD Intramural Clinical Research Center at Eastern Virginia Medical School [EVMS] [Norfolk, VA] and PROFAMILIA [Santo Domingo, Dominican Republic]. The study was approved by the Chesapeake Institutional Review Board [IRB] [#Pro00010196] and Comisiòn Nacional de Bioetica [#036–2014], respectively, and registered with ClinicalTrials.gov [#NCT02235662]. The study visits and procedures are summarized in Table 1.

**Table 1:** Study overview.

	Screening/ Enrollment	Pre-Treatment		IVR in Place					After IVR Removal	
Visit Number	V1	V2	V3	V4, Menstrual Cycle Day 6–8		V5	V6	V7	V8	V9
Menstrual Cycle or IVR Use Day		Menstrual Day 20–22	Luteal Phase	Pre-Insertion	Post Insertion	24 hours after V4	LH surge or day 17	IVR Removal, 8–10 days after V6	24 hours post removal	72 hours post removal
Informed Consent, Screening Blood and Genital Specimens	✓									
Confirm Ovulation Serum P4		✓								
Baseline Samples for Safety, PD			✓	✓						
Randomize and Initiate IVR				✓						
TFV and LNG PK Samples [Blood, CV Fluid, CM and or Tissue]					✓	✓	✓	✓	✓	✓
TFV and LNG PD Samples [CV Fluid, CM, CV Tissue]							✓	✓		
Post Treatment Safety Samples [Blood, CV Tissue]								✓		
Remove IVR								✓		

Written informed consent was obtained from all participants prior to any study procedures. Participants were healthy, 18–45 years old, had a body mass index [BMI] less than 30 kg/m^2^ and reported no use of exogenous hormones and regular menstrual cycles. All women underwent a screening visit [Visit 1, [V1]] to detect the presence of exclusion factors [e.g. bacterial vaginosis [BV], active HSV-2, *Neisseria gonorrhoeae*, *Chlamydia trachomatis*, *Trichomonas vaginalis*, HIV-1, Hepatitis B]. We instructed participants to refrain from vaginal intercourse and place nothing in the vagina during IVR use. Ovulation was confirmed by a luteal phase serum progesterone [P4] level of ≥3.0 ng/mL at visit 2 [V2]. Participants underwent baseline sampling in the luteal phase of the menstrual cycle at visit 3 [V3]. In the follicular phase of the subsequent menstrual cycle [menstrual cycle day 7 ± 1 day], we obtained additional baseline samples and randomized participants to the IVR and participants initiated IVR use at visit 4 [V4]. Blood draws were done at 1, 2, 4, and 8 hours after IVR insertion [V4] and 24 hours after IVR insertion [visit 5, V5]. Participants checked their urinary luteinizing [LH] hormone daily during IVR use with a Clearblue Advanced Digital Ovulation Predictor Kit [SPD Swiss Precision Diagnostics GmbH, Switzerland], starting on menstrual cycle day 10. We assessed their cervical mucus [CM] in the clinic within 24 hours of the LH surge or by menstrual cycle day 17, whichever came first at visit 6 [V6]. The IVR was removed 8–10 days after V6, at visit 7 [V7]. Participants filled out an acceptability questionnaire at V7. We obtained samples 24 hours after IVR removal at visit 8 [V8] and biopsies for PK in a randomly selected subset 72 hours post IVR removal at visit 9 [V9].

#### Randomization

We randomized participants in a 2:2:1 allocation ratio [TFV/LNG IVR: TFV IVR: Placebo IVR], stratified by study site, to use 1 of the 3 IVRs for approximately 15–18 days. To maximize blinding, the unequal allocation was effected by randomly assigning five blinded codes to treatment group [two codes each for the two active treatments and one for placebo]. Participants were then randomly assigned to one of these blinded codes in a 1:1:1:1:1 ratio, using permuted block randomization with randomly selected block sizes. The participants were not told which IVR they had received and the investigator and laboratory staff were blinded to the extent possible. Participants were further randomized to sampling time points for CV fluid [CVF] specimens [aspirate and swab] at 1, 2, 4, or 8 hours after insertion of the IVR at V4, and CV biopsy at either 24 [V8] or 72 hours [V9] post-removal of the IVR, in equal ratios within treatment groups.

#### Study product

TFV/LNG, TFV and placebo IVRs were manufactured under current good manufacturing practices [cGMP] at DPT Labs [San Antonio, TX] using manufacturing processes previously described [16, 17]. The unit dose for the IVRs was designed to be approximately 8–10 mg/day of TFV for 90 days of release. The TFV IVR is comprised of a hollow hydrophilic polyurethane reservoir sheath with a 0.7 mm wall thickness, 5.5 mm outer cross-sectional diameter and 55 mm outer diameter; the IVR is filled with a drug-loaded semisolid core composed of TFV [58.3%, total load of ~1.2–1.6 g TFV per IVR], glycerol [35.8%] and water [5.9%]. The TFV/LNG IVR has similar dimensions, composition and appearance to the TFV IVR except a 2 cm-long solid hydrophobic polyurethane reservoir segment loaded with 6 mg LNG, designed to release approximately 20 μg/day, and capped with 2 mm-wide hydrophobic polyurethane spacers was inserted and induction welded together. The placebo IVR has a similar appearance and dimensions to the TFV/LNG IVR; in lieu of TFV in the hollow reservoir core segment, a pre-gelatinized starch [starch 1500 partially pre-gelatinized maize starch] is used as a non-eluting filler. The IVRs do not require cold chain storage and were stored at room temperature. The mean force [in Newtons] to compress the IVRs to 10% of the diameter [F10] was 1.38 [range 1.15–1.66] for the TFV IVR, 2.40 [range 1.78–2.99] for the placebo IVR and 1.86 [range 1.40–2.30] for the TFV/LNG IVR; these values are within the range of other commercially available IVRs [18].

### Safety assessments

#### Adverse events

Treatment emergent adverse events [AEs] were the primary safety measure, along with any changes at the end of treatment in safety laboratory [complete blood count, fasting lipids, and comprehensive chemistry panel] measurements from baseline. We monitored AEs at each study visit and graded each for severity and relationship to study product or study procedures. We performed colposcopic examination of the lower genital tract prior to IVR insertion, 24 hours after insertion and at the end of treatment. We contacted the participant 1 to 2 weeks after final genital sampling to ask about AEs experienced and medications taken since the last visit.

#### Density and phenotype of immune cells in ectocervical tissue

One ectocervical biopsy prior to IVR insertion and on the day of IVR removal was placed in 10% neutral buffered formalin for 24–48 hours, transferred to an embedding cassette, and processed as per our immunohistochemistry protocol [19]. The antigens were detected using AEC chromogen—substrate kit [SkyTek labs, Mississauga, Ontario, Canada] and mounted with Accergyl mounting media [Accurate Chemicals, NY, USA]. Cell phenotype was identified using specific monoclonal antibodies against CD45, CD3, CD4, CD8, CD1a, CCR5 and HLA-DR. Positive stained cells were counted under the microscope [Nikon E-800]. In brief, 5–6 fields were randomly selected using a Nikon E800 microscope from each section and these images were captured using a CCD camera [Spot Camera, Diagnostic Instruments MI, USA]. Cell density was expressed as the mean of the counts in 5–6 fields in cells/mm^2^.

#### Semi-quantitative assessment of vaginal microbiota and Nugent score

We obtained vaginal secretions for Nugent score [20] and for a semi-quantitative assessment of vaginal microbiota just prior to IVR insertion, at V4 and just prior to IVR removal, at V7. Two Dacron swabs containing CVF were placed in a Port-A-Cul transport tube [Becton, Dickinson, Sparks, MD] and transported on ice within 24 hours of collection for cultivation of cultivable microbiota as previously described [21]. Semi-quantitative assessment of the following bacterial species were described: Anaerobic gram negative bacillus, Candida, *Escherichia coli*, *Enterococcus*, *Gardnerella vaginalis*, *Streptococcus agalactiae*, Hydrogen peroxide producing [H2O2 +] Lactobacillus, H2O2 non producing [H2O2 -] Lactobacillus and Mycoplasma. Vaginal secretions for pyrosequencing of the vaginal microbiome were also obtained at V4 and V7, but these data are still under analyses and will be reported in a separate manuscript.

#### Secreted soluble proteins from the CV mucosa

Concentrations of soluble proteins were measured in CV lavage [CVL] at V4, prior to IVR insertion and at the end of treatment, prior to IVR removal [V7]. CVLs were collected in 4 ml normal saline after speculum insertion by lavaging the cervical fornices and vaginal walls and avoiding spraying directly into the cervical os. The CVF was centrifuged at 4°C for 10 min at 800 g, and frozen at -80 °C and transported to the central lab [RF] where the supernatant was analyzed for safety biomarkers under accreditation by the College of American Pathology. ELISA was used to measure levels of the secretory leukocyte protease inhibitor [SLPI] [R&D Systems, Minneapolis, MN] and β-defensin- 2 [BD2] [Phoenix Pharmaceuticals, Burlingame, CA] using Victor2 reader [Perkin Elmer Life Sciences, Boston, MA]. Samples were screened at at 80-fold dilution for SLPI and 100-fold dilution for BD2, and all samples showing levels above the assay detection range were additionally diluted to obtain accurate measurements. Interleukin [IL]-1α, IL-6, IL-10, granulocyte-macrophage colony-stimulating factor [GM-CSF], interferon gamma-induced protein 10 [IP-10], macrophage inflammatory protein [MIP]-1α, RANTES and tumor necrosis factor [TNF]-α, and were measured in undiluted CVLs by a multiplex electrochemiluminescence [ECL] assay [Meso Scale Discovery [MSD], Gaithersburg, MD]. IL-8 and IL-1receptor antagonist [RA] were measured by separate single-plex MSD assays due to a required minimum 10-fold dilution. All measurements were performed in duplicate. A split quality control pool prepared from CVLs was tested on each plate showing inter-plate coefficient variation [CV] of 4.2% for SLPI, 5% for BD2, between 3.7% and 16.2% for the markers on the MSD multiplex plates, and 4% for IL-8 and IL-1RA.

### Acceptability assessments

Participants recorded expulsions, removals, symptoms or problems associated with IVR use and completed an acceptability questionnaire after removal.

### PK assessments

#### TFV PK

TFV concentrations in plasma, vaginal tissue close to the IVR in the posterior fornix and distal to the IVR near the vaginal introitus, and CVF collected by aspirates and on Dacron swabs were determined via previously described liquid chromatographic-mass spectrometric [LC-MS/MS] approaches [22, 23]. Quantification of TFV-diphosphate [TFV-DP] in tissue was conducted using a previously described indirect enzymatic approach [24]. All assays were validated in accordance with FDA, Guidance for Industry: Bioanalytical Method Validation recommendations [25]. Assay lower limits of quantification [LLOQ] were as follows: plasma TFV, 0.31 ng/mL; CVF [aspirate] TFV, 5 ng/mL; CVF [swab] TFV, 0.625 ng/Dacron swab; tissue TFV, 0.05 ng/sample; tissue TFV-DP; 50 fmol/sample. Results based on pre-sample measurements were normalized to net swab or biopsy weights provided by study sites or generated at the testing laboratory, and reported as ng/mg for TFV and fmol/mg for TFV-DP. Due to high TFV concentrations observed in CV aspirate, all samples were diluted prior to quantification and normalized to mL of volume. There were 25 vaginal tissue samples [obtained proximal to the IVR] from TFV containing IVR users which were compromised due to equipment malfunction during sample preparation at the lab and excluded from the analysis [8 at V5, 8 at V7, and 3 at V9].

#### LNG PK

LNG was quantified in serum by a specific and sensitive radioimmunoassay [RIA] as described previously [26]. Prior to RIA, each analyte was extracted with hexane:ethyl acetate [3:2] to remove potential interfering conjugated steroids. A highly specific antiserum was used in conjunction with an iodinated radio-ligand in the RIA. Separation of free from antiserum-bound LNG was achieved by use of second antibody. The sensitivity of the LNG RIA is 0.05 ng/mL, and intra-assay and inter-assay coefficients of variation are, on average, 4.4% and 8.9%, respectively. We also concurrently measured plasma sex hormone binding globulin [SHBG] to calculate a plasma free LNG index [ratio of LNG nmol/L to SHBG nmol/L].

### PD assessments

#### Anti-HIV activity in CVF

TZM-bl cells [27] were plated and CVL [1:5 final dilution] was applied to the appropriate wells. For toxicity testing, 100 μL of medium was added to each well. The next day, 100 μL of medium was removed and replaced with 100 mL of CellTiter-Glo, [Promega Corp.] and the luminescence measured. Viability was determined based on deviations from the cell-only control and presented as the percent viability. For efficacy testing, 100 μL of medium containing HIV-1_BaL_ was added to each well. After 48 h, 100 μL of medium was removed and replaced with 100 μL of Bright-Glo [Promega Corp.], and the luminescence was measured. Inhibition was determined based on deviations from the HIV-1-only control and presented as the percent inhibition as previously described [28, 29].

#### Anti-HSV-2 activity in CVF

Vaginal swabs were collected and placed in 500 μL of sterile normal saline and stored at -80°C until processing. Thawed samples were centrifuged at 2,000 rpm for 7 minutes at 4°C. Each swab was then centrifuged again to remove all secretions from the swab. The supernatant was collected, aliquoted into new Eppendorf tubes, and stored at -80°C. The activity of swab eluent against HSV-2 was measured within 6 months of collection as previously described using Vero [monkey kidney epithelial] cells, infected with HSV-2[G] mixed 1:1 with each CVF or control buffer as previously described [30].

#### p24 antigen production by tissue biopsies infected ex vivo with HIV-1_BaL_

An ectocervical biopsy was obtained and placed in a micro-centrifuge tube containing complete Leibovitz 15 tissue culture media [cL15, Gibco, Grand Island, NY] supplemented with 10% heat inactivate Fetal Bovine Serum [Hyclone, Logan, UT], 100 units/ml Penicillin and 100 μg/ml Streptomycin [Gibco]. Biopsy samples were kept on ice and shipped via overnight courier. HIV-1 infected biopsies were evaluated for HIV-1 p24 release. Values were normalized to tissue weight as previously described [31, 32].

#### LNG PD assessment

Contraceptive efficacy was assessed by several surrogates, including ovulation during IVR use, defined as a luteal phase serum P4 of ≥ 3 ng/mL pre-removal. At V6, we used ex vivo tests which are classically associated with fertility testing, including the Insler score and the sperm penetration assay [modified slide test] [33–38] to assess local micro-dose LNG effects on the CM. To determine the Insler score, at least 2 examiners assessed the CM on a scale of 0–3 for each factor [Spinnbarkeit, volume, viscosity, cellularity and ferning] with a score of 10 or more indicating normal, ovulatory, mid cycle mucus receptive to sperm penetration [36]. An aliquot of CM was placed on a slide and approximately 50 μL of donor sperm and the CM sperm interaction was assessed per standard guidelines [37]. Donor sperm were obtained under an EVMS IRB approved protocol [IRB #13-02-FB-0031].

### Residual drug assessments and estimated in vivo drug release rates

IVRs were stored in individual sealed foil packages at -80°C until shipped on dry ice. IVRs containing LNG segments were cut at the joint between the LNG segment end cap and the end of the sealed TFV segment to isolate the LNG segment. The LNG segments were then cut into 2–3 mm thick pieces and dissolved in dichloromethane, polyurethane was precipitated in acetonitrile, and the solution phase was filtered through a 0.2 μm polytetrafluoroethylene [PTFE] filter prior to analysis by high-performance liquid chromatography [HPLC]. TFV containing segments of IVRs were cut into 5–8 mm sections before dissolving residual TFV in 100 mL total volume of sodium phosphate buffer [100 mM, pH 7.4]. An aliquot of this solution was further diluted 100-fold before filtration through a 0.2 μm nylon membrane prior to analysis by HPLC. Analysis of TFV and LNG by HPLC was conducted similar to methods described previously [16, 17]. IVR release rates were estimated by subtracting the recovered active pharmaceutical ingredient [API] concentration result from the average control API recovery and dividing by the number of days of reported use.

### Sample size and statistical analyses

Sample size was based on feasibility rather than statistical considerations in this phase I study. We used SAS/STAT software version 9.3 [SAS Institute, Inc., Cary, NC, USA] [39] for analysis and GraphPad PRISM version 6 [San Diego, CA] for graphs. Imputation of drug concentrations below the limits of quantification [BLQ] was possible only for TFV in plasma. Planned PK parameters were maximum concentration [C_max_], time to C_max_ [T_max_ [days]], concentrations at 24 hours [C_24h_] and 15 days [surrogate for IVR removal time] [C_15days_], and areas under the curve [AUCs] through 24 hours [AUC_0-1day_] and 15 days [AUC_0-15days_]. AUCs were calculated from non-compartmental analysis using linear trapezoidal rule / linear imputation to C_max_ and log trapezoidal rule / log imputation after C_max_. If there was no blood draw on Day 15, C_15days_ was imputed from the model, and may have used the terminal-phase elimination rate constant. AUCs were computed as partial areas under the full curve, i.e. last non-zero measurement.

The effect of treatment was estimated from a general linear model with generalized estimating equations [GEE]. GEE analysis was conducted on natural-log transformed values, with treatment group and center as fixed effects, participant clustered within center, and visit as a repeated measure, assuming an unstructured correlation matrix. If the transformed values didn’t meet normality assumptions, data were dichotomized into meaningful categories and GEE modeled the treatment-group odds ratio. Depending on the objective, anatomical location of measurement [e.g., near IVR or distal] was either included as a repeated measure or, for consistency across visits, data were selected for a given anatomical location [e.g. near IVR]. Due to sparse data, randomization to final-visit biopsy sampling was ignored. This approach was also used to explore the predictive association of TFV concentration from a Dacron swab to that from tissue biopsy or CV aspirate. Correlation of TFV concentrations for pairings of measurements was evaluated non-parametrically using Spearman’s rho. The treatment effects on secreted soluble proteins and vaginal immune cell densities were evaluated post hoc using non-parametric Wilcoxon comparisons not adjusted for covariates in the GEE models.

Nominal p-values are reported, unadjusted for multiple analyses. Inferences based on statistical significance [or lack thereof] should be made cautiously due to both potentially low statistical power of tests to detect treatment-group differences [if they truly exist] as well as the number of statistical tests.

## Results

### Study population

**Fig 1:**
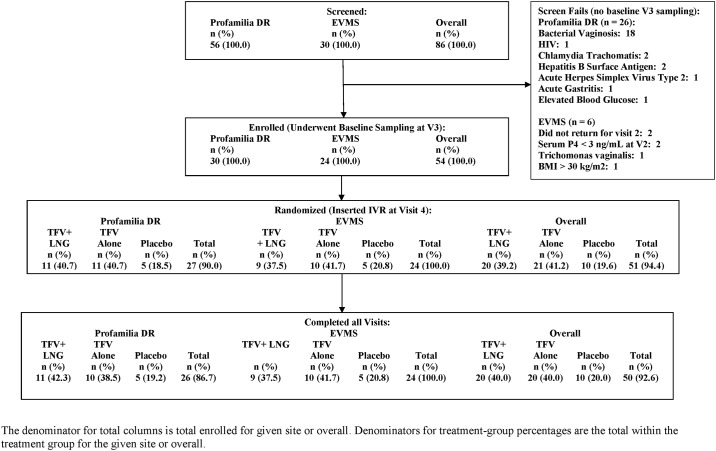
CONSORT figure CONRAD A13-128 study population.

We enrolled the first patient in October 2014 and the last patient completed the study in December 2015. As summarized in the CONSORT Fig 1, we screened 86 participants, with 51 women initiating IVR use and 50 completing all study visits. Twenty women were randomized to TFV/LNG IVR, 21 to TFV IVR and 10 to placebo IVR. The demographic data of the randomized participants are summarized in Table 2.

**Table 2:** Demographics of randomized population.

	TFV/LNG [n = 20]	TFV [n = 21]	Placebo [n = 10]	Total [n = 51]
**Age [years]**				
Mean [SD]	36.0 [4.9]	33.0 [6.3]	36.3 [4.8]	34.8 [5.6]
Median	35.0	33.0	37.5	34.0
Range	[28–44]	[23–45]	[29–44]	[23–45]
**Ethnicity**				
Hispanic/Latina	12	12	6	30
Not Hispanic/Latina	8	9	4	21
**Race**				
Black/African American	4	1	2	7
White	4	7	2	13
More than one race	11	12	5	28
Other	1	1	1	3
**Education [years]**				
Mean [SD]	12.5 [3.4]	12.7 [3.2]	12.5 [1.4]	12.6 [3.0]
Median	13.5	14.0	12.0	13.0
Range	[3–17]	[7–17]	[11–15]	[3–17]
**Partner Status**				
Living with partner	12	15	4	31
Not living with partner	4	1	4	9
No partner	4	5	2	11

### Randomization

There were no allocation errors.

### Safety endpoints

#### Duration of use and AEs

The mean duration of IVR use was consistent across treatment groups [14.6 to 15.7 days]. Total exposure to study IVRs was 9.9 person-months for the TFV/LNG group, 9.6 person-months for the TFV IVR group, and 5.1 person-months for the Placebo IVR [total: 24.6 person-months]. There were no serious adverse events [SAEs] or AEs leading to discontinuation of IVR use. One participant [TFV IVR] had two events considered possibly related to study product: a moderate headache lasting 2 days and mild vulvovaginal pruritus lasting 3 days, both of which resolved without sequelae. Only one moderate or severe urogenital AE occurred: a TFV IVR user had an episode of severe vulvovaginal pain considered related to a study procedure but not study product, which resolved without sequelae after 10 hours. No significant colposcopic findings or abnormalities in vital signs or physical exam were noted. Only one participant, randomized to the TFV IVR, had a lab result meeting the NIH/NIAID Division of AIDS criteria for grade 3 AE [40]. Her absolute neutrophils went from 0.90 K/μL at baseline [Grade 2] to 0.70 K/μL at V7 [Grade 3]. There was only one IVR discontinuation during the study, and this was due to personal reasons, not study product or procedures.

#### Ectocervical immune cells and epithelium

We found no statistically significant or meaningful differences in the epithelial thickness, number of cell layers, or density and phenotype of mucosal immune cells across treatment groups after IVR use [Table 3].

**Table 3:** Immune cells in ectocervical tissues at IVR removal [visit 7], based on treatment assignment. Completer population.

VARIABLE	Placebo IVR [n = 10]			TFV IVR [n = 20]			TFV LNG IVR [n = 18]			P value
	Mean	STD	Median	Mean	STD	Median	Mean	STD	Median	
**Epithelial Thickness [μm]**	217.46	72.95	228	213.85	87.3	209.5	224.87	94.24	216.15	0.98
**Number of Cell Layers**	20.12	5.12	19.6	20.96	5.63	21.35	22.23	5.09	21.3	0.65
**Cells in the Epithelium [cells/mm**^**2**^**]**										
**CD45**	129.09	51.04	132.35	115.27	73.91	94.8	138.41	72.62	145.05	0.44
**CD3**	99.37	44.87	102.05	87.67	62.12	67.05	97.53	54.97	93.75	0.56
**CD8**	64.38	27.53	59.75	60.16	52.25	43.75	71.63	43.8	60.05	0.33
**CD4**	0	0	0	1.12	3.63	0	2.93	5.81	0	0.11
**HLADR**	62.3	28.68	66.4	62.88	47.67	46.95	58.43	34.23	49	0.61
**CCR5**	0	0	0	0.15	0.67	0	0	0	0	0.72
**Calculated CD4 [CD3–CD8]**	34.99	28.27	34.95	27.51	14.39	25.75	25.9	18.56	20.45	0.44
**Cells in the Lamina Propria [cells/mm**^**2**^**]**										
**CD45**	115.81	45.8	104	104.75	49.01	88	125.32	96.32	89.35	0.50
**CD3**	74.05	31.7	65.3	73.42	38.97	64	91.96	75.52	64	0.78
**CD8**	49.38	26.55	40	48.39	28.1	37.3	58.65	49.11	38.65	0.92
**CD4**	3.99	4.39	4	4.91	4.26	4	8.76	12.74	6.65	0.91
**HLADR**	46.06	24.47	40.8	54.07	19.85	56	51.28	26.49	45.35	0.40
**CCR5**	3.44	2.86	3.35	5.26	7.76	3.2	3.64	7.57	1.35	0.79
**Calculated CD4 [CD3–CD8]**	24.67	16.01	21.35	25.03	14.19	20	33.31	31.62	23.85	0.45

#### Vaginal microbiota

Nugent scores were available for 41 participants prior to IVR insertion and 40 participants at IVR removal. Prior to IVR insertion, 28/41 [68.3%] participants had normal vaginal microbiota [Nugent 0–3], 8 [19.5%] participants had intermediate flora [Nugent 4–6] and 5 [12.2%] participants had developed asymptomatic BV [Nugent 7–10]. At V7, there were 4 participants who developed asymptomatic BV, 2 in the TFV IVR group and 2 in the placebo IVR group. Most participants, 26/40 [65%] had normal vaginal flora at the end of treatment. Due to the small sample size, statistical analyses of the Nugent score data were not planned.

Semi-quantitative description of the vaginal flora was available for 16 TFV/LNG IVR users, 19 TFV IVR users and 9 placebo IVR users. There were no obvious safety signals or trends in changes in the 9 tested species. Specifically, one TFV IVR user went from 3+ levels of *Gardnerella vaginalis* species to 4+ levels by the end of treatment. A total of 3 participants [2 TFV IVR users and 1 Placebo IVR user] had 0 to 3+ levels of H2O2+ Lactobacillus species at the start of IVR use and 4+ levels at the end of IVR use. No participant developed symptomatic BV during the study. Due to the small sample size and small cell size of each concentration level of each species [data not shown], formal statistical analyses were not performed.

#### Secreted soluble proteins from CV mucosa

In paired comparisons, there were no significant differences from baseline among the TFVLNG IVR users [all p values > 0.05, S1 Table]. Among TFV IVR users, there was a significant increase in IL-1α [p = 0.04] and significant decreases in the IL-1RA/IL-1α ratio [p = 0.01] and IP-10 [p < 0.01] [S1 Table]. Placebo IVR users also showed significant decreases in the ratio of IL-1RA/IL-1α and in addition in IL-8 [both p values = 0.02] with IVR use [S1 Table]. In independent group comparisons, there were no significant differences in the soluble protein content of the CVL at baseline [all p values > 0.14] or at the end of treatment [all p values > 0.06] [data not shown].

### Expulsions and adherence

There was only one reported spontaneous partial expulsion, secondary to a bowel movement, of one minute duration. This participant re-inserted the IVR and completed the study. Based on participant diaries, all participants kept the IVR in situ for the length of participation. Participants inserted the IVR in the clinic and the IVR was removed by the clinician at the end of treatment. All participants with V7 had the IVR in situ at that visit.

### Acceptability

Among the 50 participants who completed the acceptability questionnaire at IVR removal, previous use of intravaginal [NuvaRing, diaphragm, sponge] [n = 10] or intrauterine contraceptives [n = 6] was not common and the majority of participants [n = 32] normally used pads, not tampons, for menstrual protection. All but 2 participants reported that the IVRs were very or fairly easy to insert. The majority [n = 47] reported that they never were aware of the IVR during daily activities and the IVR was usually comfortable during use [n = 48]. Participants chose from a list of potential worries that they had regarding IVR use. Top choices included the IVR coming out by accident [n = 14] and the IVR not staying correctly in place [n = 12], although none of these problems were realized in the study. Although most participants [n = 35] reported that they were not worried at all about acquiring HIV-1 in the future, 44 said they would be likely or very likely to use the IVR in the future as an HIV-1 microbicide or an MPT.

### TFV PK assessments

#### TFV in plasma

In the first 8 hours of IVR use, the median plasma TFV levels were below the LLOQ [0.31 ng/mL]. Prior to IVR removal, plasma TFV levels rose to a median of 2.3 ng/mL [range 0.2–17.1 ng/mL]. Within 24 hours of removal, median plasma TFV levels fell to below LLOQ values. Nearly two-thirds [66%] of all plasma TFV concentrations throughout this study were below the LLOQ. S2 Table shows the calculated plasma TFV PK parameters in participants randomized to the TFV and TFV/LNG IVRs combined.

#### TFV in CV aspirate

**Fig 2:**
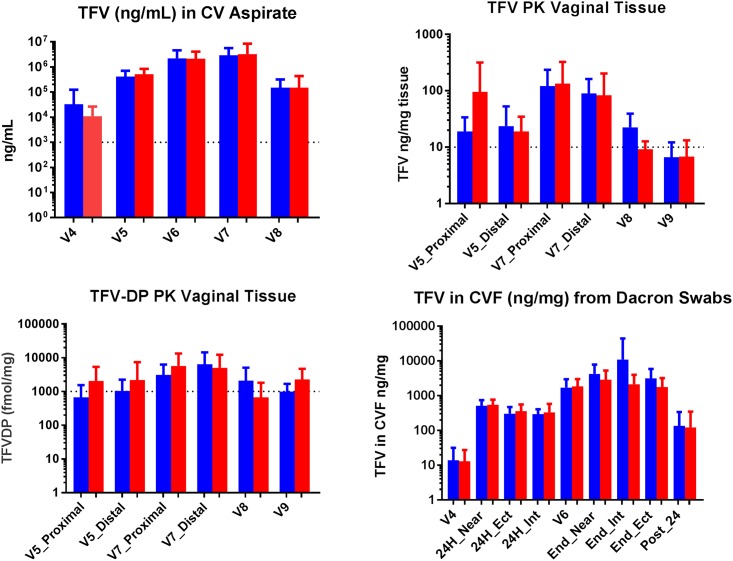
**(A) TFV in CV aspirate**. Blue Bars: TFV IVR, Red Bars: TFV/LNG IVR, Visit 4, pooled across sampling times of 1, 2, 4 and 8 hours post insertion [n = 18 for each IVR group], Visit 5: 24 hours post insertion [n = 20 for TFV and n = 19 for TFVLNG IVRs], Visit 6: LH surge or Menstrual Cycle day 17 [n = 20 for each IVR group], Visit 7: Prior to IVR Removal [n = 19 for each IVR group], Visit 8: 24 hours post removal [n = 15 for TFV and n = 20 for TFVLNG IVRs]. Reference line indicates 1,000 ng/mL, aspirate level associated with 75% reduction in HIV acquisition in CAPRISA 004 study subset [41, 42] **(B) TFV [ng/mg] in vaginal tissue**. Blue Bars: TFV IVR, Red Bars: TFV/LNG IVR, Proximal indicates biopsy was obtained close to the IVR, in the posterior vaginal fornix, Distal indicates biopsy was obtained farther from the IVR, in the lower 1/3 of the vagina closer to the introitus. Visit 5: 24 hours post insertion [for proximal biopsies n = 15 for TFV and n = 16 for TFVLNG IVR. For distal biopsies, n = 19 for TFV and n = 20 for TFVLNG IVR]. Visit 7: Prior to IVR Removal [for proximal biopsies n = 15 for TFV and n = 17 for TFVLNG IVR] and [for distal biopsies, n = 19 for TFV and n = 20 for TFVLNG IVR]. Visit 8: 24 hours post removal [n = 9 for TFV and n = 8 for TFVLNG IVR], Visit 9 72 hours post removal [n = 5 for TFV and n = 9 for TFVLNG IVR]. Dashed line indicates TFV level [10 ng/mg] associated with high TFV-DP concentrations of approximately 1,000 fmol/mg [24, 41, 42]. **(C) TFV-DP [fmol/mg] in vaginal tissue**. Blue Bars: TFV IVR, Red Bars: TFV/LNG IVR. Proximal indicates biopsy was obtained close to the IVR, in the posterior vaginal fornix. Distal indicates biopsy was obtained farther from the IVR, in the lower 1/3 of the vagina closer to the introitus. Visit 5: 24 hours post insertion [for proximal biopsies, n = 14 for TFV and n = 16 for TFVLNG IVR. For distal biopsies, [n = 19 for TFV and n = 20 for TFVLNG IVR], Visit 7: Prior to IVR Removal [for proximal biopsies, n = 14 for TFV and n = 17 for TFVLNG IVR]. For distal biopsies, n = 19 for both IVRs], Visit 8: 24 hours post removal [n = 8 for TFV and n = 7 for TFVLNG IVR], Visit 9 72 hours post removal [n = 5 for TFV and n = 9 for TFVLNG IVR]. Dashed line indicates levels found to be protective against SHIV transmission in non-human primates [43, 44] **(D) TFV in CV fluid [ng/mg] obtained from lower genital tract swabs**. Blue Bars: TFV IVR, Red Bars: TFV/LNG IVR, Visit 4: 1–8 hours post IVR insertion vaginal swab taken near IVR [n = 20 combined observations for TFV IVR and TFVLNG IVR], Visit 5: 24 hours post insertion taken near IVR [n = 20 for both IVRs], ectocervix [n = 20 for both IVRs] and introitus [n = 20 for both IVRs], Visit 7: Prior to IVR removal, taken near IVR [n = 20 for both IVRs], ectocervix [n = 20 for both IVRs] and introitus [n = 20 for TFV and n = 18 for TFV/LNG IVR], Visit 8: 24 hours post removal [n = 19 for TFV and n = 20 for TFVLNG IVR]. Cx = Ectocervix, Int = Introitus, IVR = vaginal near IVR.

In general, we found high local levels of TFV in CVF within the first hour of insertion [data not shown], and these levels remained high even 24 hours after removal of the IVR [Fig 2a]. Within 4 hours of insertion, median TFV concentrations exceeded 1,000 ng/mL and 75% of participants had concentrations of at least 933 ng/mL. By V6, the mean and median CV aspirate concentrations exceeded 10^6^ ng/mL. By the end of treatment, all evaluable samples [n = 38] had TFV concentrations in the CV aspirate above 66,000 ng/mL and, similar to V6, mean and median CV aspirate concentrations exceeded 10^6^ ng/mL. At 24 hours post removal, mean and median TFV concentrations exceeded 1,000 ng/mL. The effect of treatment group on TFV concentrations showed no significant difference on TFV concentrations in CV aspirate [p = 0.29, data not shown].

#### TFV and TFV-DP in vaginal tissue

Levels of TFV in vaginal tissues ranged from 2.0–822.5 ng/mg [Fig 2b] between 24 hours after use up until 72 hours post IVR removal. Within 24 hours of insertion, mean [median] TFV concentrations in the proximal and distal biopsies were 58.5 [17.1] and 21.3 [11.7] ng/mg, respectively. By the end of treatment [V7] mean [median] TFV concentrations in the proximal and distal biopsies were 127.7 [57.0] and 86.4 [46.7] ng/mg respectively. Mean [median] TFV concentrations were 16.2 [11.2] and 6.7 [4.5] ng/mg at 24 hours and 72 hours post IVR removal, respectively.

Within 24 hours of IVR insertion, mean [median] TFV-DP concentrations in proximal and distal vaginal tissue biopsies for all TFV containing IVR users were 1401 [415.8] and 1629 [422.2] fmol/mg respectively [Fig 2c]. By the end of treatment, the mean and median TFV-DP concentrations in proximal and distal vaginal tissues biopsies all exceeded 1,000 fmol/mg. Once the IVR was removed, TFV-DP levels declined, but overall, remained high even 72 hours post removal [mean = 1814 fmol/mg, median = 892 fmol/mg].

In post-hoc paired comparisons of TFV and TFV-DP concentrations from biopsies obtained proximal and distal to the IVR, we did not find significant differences in the concentrations of TFV or TFV-DP [data not shown, all p values > 0.09].

#### TFV in CV swabs

To explore a less invasive assay of measuring TFV levels in the genital tract, we assessed correspondence of TFV concentrations in CVF [obtained with Dacron swabs versus aspirates] and tissue biopsies at three locations in the genital tract: near the IVR in the vagina [proximal, near], ectocervix [Ect] and lower 1/3 of the vagina [introitus, distal from the IVR] [Fig 2d]. GEE modeling estimated that TFV tissue biopsy concentration would be two-thirds [0.67] that of a swab concentration [95% CI 0.41 to 1.1] and the concentration from CV aspirate estimated to be 1.89 that obtained from a swab [95% CI 1.54 to 2.31].

Correlation coefficients of concentrations from vaginal swabs and CV aspirate were significant [p values < 0.0001]: Concentrations obtained at the same anatomical location by swabs versus aspirate were highly correlated [near IVR: rho = .86; introitus: rho = .62] as were swab concentrations obtained at the same time point at different anatomical locations [near IVRversus introitus] [rho = .82]. As with CV aspirate and tissue, the effect of treatment group on TFV concentrations showed no significant difference on TFV concentrations in CV swabs [p = 0.23, data not shown].

### TFV-PD assessments

#### Anti-viral activity against HIV-1 in CVF

**Fig 3:**
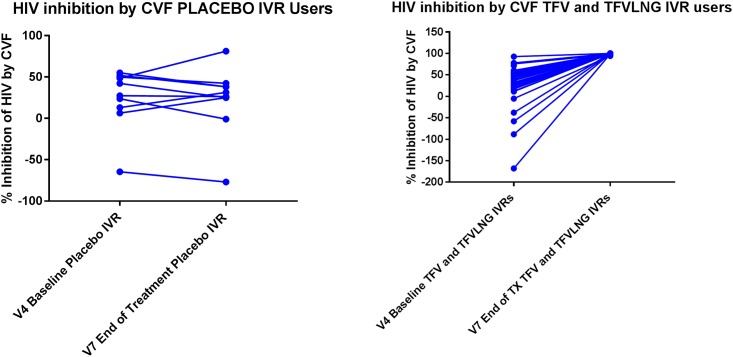
(A) HIV inhibition by cervico-vaginal fluids in vitro among placebo IVR users at baseline [visit 4] versus end of treatment [visit 7]. (B) HIV inhibition by cervico-vaginal fluids in vitro among TFV and TFV/LNG IVR users at baseline [visit 4] versus end of treatment [visit 7].

As outlined in Table 4 and Fig 3a and 3b, among placebo IVR users, there was no significant change in the anti-HIV-1 activity of CVF in vitro from baseline to after IVR use, whereas among TFV containing IVR users, the anti-HIV activity of CVF increased significantly from baseline to a range of 94–100% inhibition after treatment [V7].

**Table 4:** Percent inhibition of HIV-1 by cervico vaginal fluid in vitro.

RING	Visit 4 [Baseline Follicular Phase] [% Inhibition Compared to HIV-1 Growth in Media]			Visit 7 [At IVR Removal] [% Inhibition Compared to HIV-1 Growth in Media]			P value
	Median	Mean	STD	Median	Mean	STD	
TFV + LNG IVR [n = 20]	44.1	22.8	54.3	100.0	99.6	0.7	< 0.001
TFV IVR [n = 21]	44.0	38.2	37.5	100.0	99.6	1.4	< 0.001
Placebo IVR [n = 10]	34.5	25.3	36.1	29.0	23.0	40.6	0.34

#### p24 antigen production in tissue biopsies infected ex vivo with HIV-1BaL

In post hoc analysis we compared p24 antigen production between samples obtained at baseline [all arms; V3] and those obtained at the end of use [V7] of placebo and TFV-containing IVRs. Table 5 shows that the mean production of HIV-1 p24 antigen in tissue is lower in the presence of TFV. Lack of increased production of p24 throughout the culture period [21 days] in tissues exposed to TFV suggests significant inhibition of HIV-1 replication. These data, however, have considerable variability. When selecting participants whose baseline tissue samples were infected, p24 production [means and medians] in their tissues exposed to TFV after IVR use was several fold lower than that of baseline samples [data not shown].

**Table 5:** Comparison of viral replication [P24 antigen production] in ectocervical biopsy tissue obtained from participants at baseline [visit 3] and post-treatment [visit 7] after use of placebo, TFV and TFV/LNG rings.

P24 antigen production [pg/mg tissue] at time points in 21 day tissue culture	Luteal Phase Baseline [Visit 3] from Placebo, TFV and TFVLNG Rings [n = 24]			Post-treatment [Visit 7] from TFV/LNG and TFV Rings Combined [n = 19]			Post-treatment [Visit 7] from Placebo Rings [n = 5]		
	Mean	SD	Median	Mean	SD	Median	Mean	SD	Median
**p24 baseline**	0.44	0.43	0.36	0.42	0.37	0.38	0.50	0.67	0.30
**p24 7 days**	31.25	58.62	15.2	27.1	38.42	16.67	31.51	38.8	15.65
**p24 14 days**	59.05	176.86	16.97	17.59	14.55	12.82	23.87	28.15	16.23
**p24 21 days**	213.94	758.29	13.68	15.53	8.03	15.38	59.68	108.32	13.91
**P24 Cumulative**	305.6	938.19	55.15	60.64	50.89	46.27	115.56	174.51	46.18
**P24 Maximum**	227.52	756.16	26.88	32.45	37.99	19.66	62.99	106.53	16.23
**p24 Area Under the Curve**	1388.9	3925.51	322.64	368.67	343.2	256.18	598.28	843.96	273.21

#### Anti-viral activity against HSV-2 in CVF

Using GEE modeling, comparing each active treatment to placebo and the two active groups to each other showed no evidence of anti-viral activity against HSV-2 in CVF [data not shown]. This may be due to over-dilution of the CVF during collection and processing in regard to TFV HSV-2 inhibitory concentration. It also reflects the less potent activity of TFV against HSV-2 compared to HIV-1 in vitro[45].

### LNG PK assessments

#### LNG in plasma

**Fig 4:**
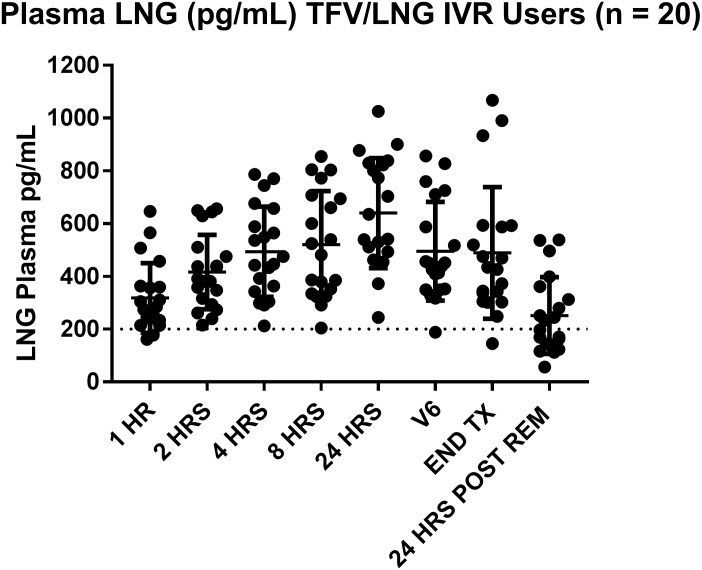
Plasma LNG [pg/mL] in TFV/LNG IVR users [n = 20]. 1 Hr = 1 hour post IVR insertion. 2 HRS = 2 hours post IVR insertion. 4 HRS = 4 hours post IVR insertion. 8 HRS = 8 hours post IVR insertion. 24 HRS = 24 hours post IVR insertion. V6 = Visit 6 [performed at the urinary LH surge or menstrual cycle day 17, whichever came first]. END TX = End of Treatment, at V7, 8–10 days after V6. 24 HRS POST REM = 24 hours after IVR removal. Dashed line indicates concentration above which data support contraceptive efficacy of systemic LNG methods [reviewed in [46]].

Participants using the TFV/LNG IVR [n = 20] had mean LNG plasma concentrations exceeding 300 pg/mL throughout IVR use [Fig 4]. Mean LNG concentrations at 1, 2, 4 and 8 hours after IVR insertion were 319, 417, 493 and 520 pg/mL, respectively. At 24 hours post insertion, V6 and end of treatment, mean LNG concentrations were 640, 495 and 489 pg/mL, respectively. Within 24 hours of removal [V8], plasma levels fell to a mean of 252 pg/mL [range 56–538 pg/mL]. Plasma LNG PK parameters are summarized in S2 Table.

By 8 and 24 hours post insertion, the mean free LNG index was 3.2 [range 1.5–5.7] and 4.2 [range 2.0–6.7], respectively. At removal, the mean index was 3.3 [range 1.5–6.0] and decreasing to 1.7 [range 0.6–3.2] 24 hours after IVR removal.

### LNG PD assessment

We pre-specified the criterion for pregnancy protection as the presence of one or more of the following surrogates of contraceptive efficacy: anovulation, an Insler score of <10 indicating poor CM quality and abnormal sperm penetration into the cervical mucus. TFV/LNG IVR users had a significantly higher proportion of participants with anovulation, poor CM quality and abnormal sperm penetration into the CM [Table 6]. In some instances, there was not enough CM to assess both the Insler score and the sperm penetration assay. All TFV/LNG IVR users had poor CM quality. The majority [95%] had poor CM and anovulation or abnormal sperm penetration and 53% had anovulation, poor CM and abnormal sperm penetration.

**Table 6:** Surrogates of contraceptive efficacy in completer population.

VARIABLE	TREATMENT GROUP		
	TFV+LNG IVR [N = 20]	TFV Alone IVR [N = 20]	Placebo IVR [N = 10]
**Visit 7: Pre-Removal Serum P4 [ng/mL]**			
< 3	11 [55.0]	5 [25.0]	3 [30.0]
> = 3	9 [45.0]	15 [75.0]	7 [70.0]
Mean [SD]	3.9 [3.60]	8.2 [5.34]	7.8 [6.44]
Median [Interquartile Range]	2.8 [1.0 to 5.7]	9.1 [2.9 to 11.6]	7.6 [0.6 to 11.7]
Range [Min to Max]	[0.3 to 13.5]	[0.3 to 17.9]	[0.2 to 19.8]
**Visit 6: Cervical Mucus Insler Score [0–15]**			
Poor [< = 10]	19 [100]	9 [45.0]	4 [40.0]
Good [> 10]	0 [0.0]	11 [55.0]	6 [60.0]
<7	14 [73.7]	7 [35.0]	1 [10.0]
7–10	5 [26.3]	2 [10.0]	3 [30.0]
>10	0 [0.0]	11 [55.0]	6 [60.0]
Mean [SD]	4.4 [2.71]	9.6 [4.03]	10.2 [2.66]
Median [Interquartile Range]	3.0 [2.0 to 7.0]	11.5 [5.5 to 13.0]	11.0 [9.0 to 12.0]
Range [Min to Max]	[1.0 to 9.0]	[3.0 to 15.0]	[5.0 to 13.0]
**Visit 6: Sperm Penetration Assay**			
Abnormal Results	5 [35.7]	6 [31.6]	0 [0.0]
Normal Results	3 [21.4]	10 [52.6]	8 [88.9]
Penetration with Poor Motility	1 [7.1]	1 [5.3]	0 [0.0]
Missing Data [not enough CM for assay] or Poor Result	5 [35.7]	2 [10.5]	1 [11.1]

### Estimated in vivo release rates from IVRs

Average estimated release rates of TFV were [mean ± std] 5.68 ± 8.72 mg/day and 11.32 ± 8.88 mg/day for TFV/LNG IVRs and TFV IVRs, respectively. The average estimated release rate of LNG from TFV/LNG IVRs was 48.15 ± 17.74 μg/day. The estimated release of LNG is higher than expected likely due to a higher release rate at the beginning of IVR use and the relatively short number of days used to calculate the in vivo rate.

## Discussion

The IVRs were safe and well tolerated. Under the conditions of this study, use of the TFV-based IVRs, with and without LNG, did not cause macro- or microscopic alterations of the CV mucosa or its microbiota. A previously tested LNG IVR [silicone-based] developed by the World Health Organization [WHO] to release ~20 ug/day of LNG had a larger cross sectional diameter [9.5 mm] and resulted in colposcopic findings in 35% of participants in an early safety study [47]. When the cross sectional diameter of the WHO IVR was reduced [to 6.0 mm], to provide greater flexibility, no clinically significant colposcopic findings were found in a multi-center randomized study using the placebo version of this IVR [48]. Similarly, we found no colposcopic findings with any of the IVRs used in this study, and no histological evidence of epithelial or mucosal changes after a mean of 15 days of use.

After the conclusion of this study, in vitro modeling data published by another group raised concern that TFV or more potent pro-drugs might impair wound healing in the female reproductive tract [49]. This could potentially uncover safety signals when topical ARVs are used in sexually active women, as consensual vaginal intercourse can cause micro-trauma to the CV mucosa [50]. It is, however, reassuring that topical TFV [gel] has been used by thousands of sexually active women in Phase II/III trials, with peri-coital or daily dosing, and demonstrated a good safety profile [8, 10, 51]. We utilized colposcopy to assess alterations of the lower genital tract mucosa as a primary safety endpoint in this trial. Colposcopy of the lower genital tract is a classic, standard safety endpoint, used for decades to evaluate intravaginal devices and topical products, and was therefore included in this phase I first-in-woman study [52]. However, colposcopy has been re-evaluated [53] and other sub-clinical safety assessments, such as changes in levels of soluble immune mediators have been correlated with mucosal tissue damage [54–56] or linked with increased risks of HIV shedding and/or microbiota disruptions in users of depot medroxyprogesterone acetate [DMPA] and combined oral contraceptives including LNG [57, 58]. Changes in the density and phenotype of genital tract immune cells and HIV-1 target cells have also been advocated in early phase I trials [59].

We did not find any significant increases in CV mucosal HIV-1 target cells with IVR use. Furthermore, there were no histological signs of epithelial compromise. The small increase in IL-1a and reciprocal decrease in the IL-1RA:IL-1 ratio in TFV IVR users was not accompanied with other signs of tissue damage, inflammation or toxicity such as increased pro-inflammatory cytokines and chemokines and global decreases in protective antimicrobial proteins.

Participants had no AEs related to device usage that would heighten suspicion for impaired genital mucosal integrity, but we recognize that women used the IVR for a short duration and were sexually abstinent. Of note, in CONRAD’s ongoing 3 month safety, PK and PD study of the TFVLNG IVR [CONRAD A15-138, ClinicalTrials.gov # NCT03279120], participants using the TFV/LNG IVR are allowed to have vaginal intercourse, which will make the findings more generalizable to an at risk population. Thus far, preliminary data from this study do not show any safety signals.

Recently, a 90-day phase I trial of the tenofovir disoproxil fumarate [TDF] IVR in sexually active women was prematurely terminated due to grade I genital ulcers in 8 of 12 women randomized to the TDF IVR; no ulcers were observed in the 5 participants randomized to the placebo IVR [60]. The TDF IVR and the TFV IVR are however different products. The IVRs are comprised of different polyurethane polymers, release different active drugs [TDF and TFV], contain different excipients, and exhibit different release profiles. Unlike TFV, TDF is a prodrug that is hydrolyzed primarily within cells but may also be hydrolyzed when it comes in contact with CVF, releasing fumarate/fumaric acid and potential degradation products such as formaldehyde [61, 62]. While one possible hypothesis for the cause of the grade 1 genital ulcers seen in the phase I TDF IVR study is the effect of sustained TFV-DP concentrations, another plausible hypothesis is that the epithelial lesions are related to TDF degradation products, alone or synergized by semen or mechanical effects of sex. There is ongoing in vitro work to understand the relative contribution of several factors to mucosal injury with TDF and other TFV prodrugs, including the effect of breakdown products [fumarate or formaldehyde], exogenous systemic hormones, semen, and intercourse. Our colleagues noted several changes in CV cytokines and chemokines when sexually active women used the TDF IVR [60]. In the current study, we report no changes in these endpoints with the TFVLNG IVR and minimal changes when participants used the TFV or placebo IVR. In our current ongoing study of the TFVLNG and placebo IVRs [CONRAD A15-138], we are measuring secreted CV soluble proteins at baseline and after sexually active women use the IVR for 3 months.

The maximum plasma concentration of TFV was well within the low range expected from topical, as opposed to oral, dosing of TFV [24, 63, 64]. Surrogates of protection against HIV-1 for TFV containing topical microbicides are currently modeled by concentrations of TFV in the CV aspirate and its association with efficacy data in the CAPRISA 004 study [41]. A CV aspirate TFV concentration over 100 ng/mL conferred an estimated 65% protection against HIV-1 acquisition, while a CV aspirate TFV concentration of over 1,000 ng/mL provided an estimated 76% protection against HIV-1 [41, 42]. Using these data as the benchmark for CVF TFV concentrations, the TFV and TFV/LNG IVRs deliver high TFV local concentrations which should be as effective as adherent TFV gel users in preventing HIV-1. In past studies, tissue concentrations of TFV following the administration of TFV vaginal gel as a single dose, two doses or 14 daily doses, with or without intercourse, were highly variable ranging from 5.3 ng/mg– 258 ng/mg [24, 63, 64]. High TFV-DP concentrations were correlated with TFV concentrations of at least 10 ng/mg of tissue [24, 63, 64]. Our data support that the TFV and TFV/LNG IVRs deliver sustained TFV concentrations to the vaginal tissues similar to concentrations seen with TFV gel, and should be protective.

This is reinforced by tissue levels of TFV’s active metabolite, TFV-DP. The benchmark of 1,000 fmol/mg for TFV-DP levels in tissue comes from PK and efficacy studies of TFV gel in macaques, demonstrating that TFV gel, when applied 30 minutes [44] or even 3 days [43] prior to simian human immunodeficiency virus [SHIV] challenge, protected all or the majority of macaques, respectively. Our data demonstrate that both TFV and TFV/LNG IVRs resulted in high median concentrations of TFV-DP, which remained high even after IVR removal. Maintaining drug concentrations post removal is important, since the IVR is a less familiar dosage form in sub-Saharan Africa and previous acceptability studies indicate that some women would want to remove IVRs during intercourse or menses [65, 66]. We acknowledge that participants used the IVRs for an average of 15 days. Although the TFV concentrations in CV aspirate were high at both V6 and V7, and tissue data at the end of treatment met established benchmarks, we will confirm steady state concentrations of TFV and TFV-DP in all compartments during an extended, 90 day PK study, [CONRAD A15-138 Protocol], which is currently enrolling participants.

Consistent with our TFV measurements in vaginal tissues obtained proximal or distal to the IVR, tissue concentrations of DPV after DPV IVR use were similar in tissues obtained from the introitus and cervix [67]. Our tissue data from sites distal and proximal to the IVR support TFV was uniformly distributed throughout the vagina. Although there are no established animal or human efficacy data for TFV concentrations in CV swabs, our correlations of TFV concentrations in tissue, CV aspirate and CV swabs may provide the foundation for developing future PK benchmarks using less invasive sampling methods.

The inhibitory activity of the CV secretions against HIV-1 at baseline was similar to previous data in healthy women [30, 31, 68]. Consistent with the high local levels of TFV, once participants were exposed to TFV containing IVRs, the inhibitory activity of the CVF against HIV-1 in vitro increased significantly, similar to levels seen with use of TFV vaginal gel [23, 30, 69]. Our tissue PK data supports that anti-HIV efficacy would be achieved with TFV containing IVRs in many users. Lack of increased production of p24 throughout the culture period in tissues exposed to TFV suggests significant inhibition of HIV-1 replication, but the in vitro/ex vivo model used to assess activity was not able to show statistically significant reductions in p24 antigen production. This is likely due to the high inter and intra assay variability in p24 antigen production in ex vivo biopsy challenge experiments [70].

Although not the primary outcomes of the trials, topical TFV gel reduced acquisition of genital HSV-2 compared to placebo in two phase IIb trials [7, 8, 11]. In this study, levels of TFV in the CV aspirate were consistent with HSV-2 protection [7]. However, CVF collected during IVR use did not show higher inhibition of HSV-2 in vitro when compared to baseline. This likely reflects sample processing which results in dilution of the CVF to below the concentration required to inhibit HSV-2 [71]. We are working to improve sample preparation for this in vitro assessment of anti-HSV activity for future studies, including challenge of biopsy tissue ex vivo with HSV-2.

After completion of this study, data from other groups indicating that BV associated microbiota could degrade TFV in the CV fluid [72, 73] among TFV 1% vaginal gel and TFV vaginal film users were published. Although clinical BV was an exclusionary criterion in this study at screening [V1], we did find that a small number of women had Nugent scores consistent with asymptomatic BV at IVR insertion [V4] and at the end of treatment [V7]. Our Nugent score and semi-quantitative vaginal culture data do not show any concerning safety signals. Although semi-quantitative assessment of cultivatable species is a less sensitive methodology than pyrosequencing, this standard assessment of the microflora has been used in numerous safety studies of contraceptive IVRs, including a recent one year contraceptive IVR [74] and was therefore included in this first in woman study. We obtained vaginal swabs for pyrosequencing of the microbiome at baseline and after IVR use and found that these data were consistent with our reported findings that the IVRs had no adverse effect on the vaginal microbiota. Because pyrosequencing was an exploratory endpoint beyond the scope of this manuscript, we plan to publish these data separately. We recognize that BV is very common in high HIV-1 incidence areas and that the impact of BV on TFV PK remains an important issue. In addition, our pyrosequencing data support that the high mucosal TFV and TFV-DP concentrations achieved by these IVRs are not significantly different in women with Lactobacillus dominated or community state type 4 microbiota [75].

Changes in tissue gene expression and proteomic analyses of CV secretions have recently been added to the armamentarium of safety endpoints, primarily for rectally applied microbicides [76–79]. In this study, we obtained a vaginal biopsy to evaluate changes in gene expression from baseline. These data are currently under analysis and will be published separately.

The contraceptive efficacy of a 20 μg/day LNG IVR was previously demonstrated in two large, phase 3 clinical studies, enrolling over 2,700 women [80, 81]. The expulsion rates of these larger IVRs were higher [82] than what has been observed with the etonogestrel/ethinyl estradiol contraceptive vaginal IVR [83]. Our IVR has similar dimensions and flexibility to the current, marketed contraceptive IVR [18]. In addition, in the UK study, 61% of women found the LNG IVR to be acceptable or very acceptable [81]. Although recent adherence data from the DPV IVR trials indicates variable adherence with age [12, 13], we believe that acceptance of an IVR for both contraception and HIV-1 prevention has changed since the WHO LNG IVR trials [84]. Importantly, we believe that an MPT option would be highly desirable for many women, ultimately increasing adherence and efficacy.

We chose the 20 μg/day LNG dose based on the wealth of previous safety, PK and efficacy data and the current trend in contraceptive development to reduce systemic side effects and menstrual cycle disruption by focusing on local and/or microdose products [85]. Data support that high dose systemic progestin, specifically DMPA may be a co-factor in HIV-1 acquisition [reviewed in [86]]. To our knowledge there are no published clinical data about the effect of topical LNG on mucosal factors facilitating HIV-1 infection. The data presented in this paper may be the first. Unlike both DMPA and oral, systemic LNG combined with estrogen [58, 87], the locally applied LNG combined with TFV in the IVR device did not cause any significant changes in soluble CV biomarkers in our trial. According to our findings, unlike systemic DMPA [88], the tested micro-dose of topical LNG did not increase the number of mucosal immune cells or their activation status. Also unlike DMPA [77], local LNG did not decrease the number of epithelial layers or compromise their integrity. Many of the adverse effects observed with DMPA may be due to induced hypoestrogenism [89], an effect not seen with the TFV/LNG ring. Other effects of DMPA underlying its associated increased susceptibility to HIV-1 have been linked to activation of the glucocorticoid receptor [90]. LNG has much lower affinity for this receptor than DMPA [91, 92].

An essential mechanism of the microdose LNG is local effect on CM, which has been characterized with the LNG intrauterine system [93], but have not correlated with plasma LNG concentrations measured from systemically administered contraceptives [reviewed in [46]]. Plasma levels of LNG, measured in previous studies of the 20 ug LNG IVR, ranged from 187–682 pg/mL [94–96]. Plasma levels of LNG in LNG implant [Norplant, Jadelle] users generally exceed a mean of 200–300 pg/mL [97–100], measured by RIA, and are often considered the plasma benchmark for contraceptive efficacy [reviewed in [46]]. We used RIA to provide comparisons to these benchmark data from other LNG based contraceptives [97–100]. Our data support that the TFV/LNG IVR delivers levels of LNG consistent with previously tested LNG IVRs from the WHO [94–96] and in the range of effective systemic LNG implants [97–100] and current, microdose LNG contraceptive intrauterine systems [101, 102]. The free LNG index is a surrogate to estimate free unbound exogenous hormone, as LNG is highly bound to SHBG, and is described for other long acting systemic LNG based contraceptives [99, 103–105]. The range of free LNG indices measured in this study is within the range seen in the last 2 years of LNG implant use, a time of high contraceptive efficacy despite normal luteal activity [105].

We recognize that there is an inverse relationship between plasma LNG levels and BMI, suggesting potentially lower contraceptive efficacy among overweight or obese women [106, 107]. A limitation of this study is that we did not enroll participants with a BMI of 30 kg/m^2^ or higher. Obesity was hypothesized as a potential cause of reduced contraceptive efficacy in previous microdose LNG IVR trials [108]. We plan to investigate the extended and PK tail concentrations of the IVRs in a 90 day safety, PK and PD study and plan to enroll women with a BMI of 30 kg/m^2^ and higher in future studies. Ultimately, the contraceptive efficacy of this product will need to be demonstrated in clinical trials, and our initial LNG PK data are promising.

Participants randomized to the TFV/LNG IVR had higher anovulation rates, CM alterations and abnormal sperm penetration than participants using the other IVRs. Ninety-five percent of women using the TFV/LNG IVR showed two or more surrogates of contraceptive efficacy, and 100% showed at least one. The anovulation rate [55%] was similar to that seen in previous studies of LNG microdose contraceptive IVRs [95, 109–111]. CM for Insler score, sperm penetration and ovulation was assessed only once during this relatively short treatment, but the evaluation was timed to urinary LH surge. Despite this deliberate timing, we found some anovulation, poor CM and abnormal sperm penetration even in the non-LNG groups. It is likely that some TFV or placebo IVR users either experienced an anovulatory cycle or that our measurements were mistimed despite our best efforts. We plan to use twice weekly serum estradiol and P4 testing to further pinpoint the window of optimal CM testing and confirm our findings of high rates of local CM effects and moderate rates of anovulation in the 90 day TFV/LNG IVR study.

The estimated release rate for TFV was in the expected range for both IVRs. The release rate for LNG was higher than the 20 μg/day expected for average steady state release rate over 90 days. As seen in in vitro studies, a small burst of LNG is observed initially when the IVR is placed for the first time [16]. When averaging release rates from 15 days of use rather than 90 days as designed, the estimated daily rates are expected to be higher and have high standard deviations. We expect to refine estimates of the average steady state release rates in our current study of the TFVLNG IVR over the full 90 day dosing regimen. We also developed additional objective measures of adherence to IVR use for these IVRs since the conduct of this study [112, 113], which will assist in the analysis for the 90 day study.

The majority of participants had little to no experience with intravaginal products for contraception or menstrual protection, but found the product to be acceptable and easy to use. We recognize that this initial study of the TFV and TFV/LNG IVR was performed in a highly selected population of women who were at low risk of HIV acquisition and who were not currently at risk of pregnancy. This population is ideal for a phase I study, but acceptability is most relevant when evaluated among ultimate end users of this product. As participants were sterilized or abstinent, our acceptability assessments focused primarily on physical symptoms and problems or perceived issues with product use, not motivators for future use. Participants refrained from sexual activity during use of this initial safety study, and therefore we have no data on IVR use with sexual activity. We have conducted end user research in South Africa and found that an MPT was highly desired by young women and found that an MPT was a highly desirable aspect for young women [114].

## Conclusions

This first-in-woman study of two IVRs releasing TFV and TFV/LNG showed that the IVRs were well tolerated and safe. Uniquely, these IVRs are capable of releasing in a controlled manner two very different drugs in amounts that differ by roughly 500-fold. Drug release from the IVRs achieved high local concentrations, compatible with HIV protection and contraceptive efficacy. The TFV/LNG IVR is being tested for its intended duration of use in an extended 90 day safety, PK and PD study in women [CONRAD A15-138 study] and other studies with both IVRs are planned. Maintaining high local concentrations post IVR removal [dosing forgiveness] and multipurpose prevention of STIs [primarily HIV-1 and HSV-2] and pregnancy are value-added features of these IVRs. Ultimately, for MPTs to be successful, each component must offer end users a contraceptive and anti-infection option that fits their lifestyle and adds benefits to their current prevention practices. The ultimate efficacy of this MPT must be tested in future prevention trials. We propose that these IVRs will fill an important gap in the current prevention methods that women, particularly those in less developed countries, can utilize to protect themselves from HIV-1, HSV-2 and unintended pregnancies.

## Supporting information

S1 Table Changes in soluble proteins in the CV supernatant with IVR use. (DOCX)

S2 Table TFV and LNG pharmacokinetics summary table. (DOCX)

S1 Fig CONSORT 2010 flow diagram. (DOC)

S1 Checklist CONSORT checklist. (DOC)

S1 Protocol CONRAD 128 protocol V4. (PDF)

S1 Data Demographic data. (ZIP)

S2 Data PK data. (ZIP)

S3 Data PD data. (ZIP)

S4 Data Accept adhere data. (ZIP)

S5 Data Safety data. (ZIP)
