# Supplementary material for: Randomized, placebo controlled phase I trial of safety, pharmacokinetics, pharmacodynamics and acceptability of tenofovir and tenofovir plus levonorgestrel vaginal rings in women
Source: PLoS One. 2018 Jun 28;13(6):e0199778. doi: 10.1371/journal.pone.0199778 (PMC6023238; doi:10.1371/journal.pone.0199778)
Supplement: S1 Table — (DOCX) [file pone.0199778.s001.docx]

Supplemental Table 2: Changes in soluble proteins in the CV supernatant with IVR use

| Variable (pg/mL) | Pre-Insertion (Follicular Phase, Visit 4) Median | End of Treatment (Visit 7) Median | P value |
| --- | --- | --- | --- |
| Placebo IVR |  |  |  |
| RANTES | 8.21 | 3.36 | 0.34 |
| BD2 | 45073.78 | 124223.6 | 0.34 |
| GMCSF | 0.92 | 1.02 | 0.13 |
| IL_10 | 0.68 | 0.45 | 0.13 |
| IL_1A | 178.07 | 188.11 | 0.34 |
| IL_1RA | 347760.8 | 175149.2 | 0.11 |
| IL_6 | 14.72 | 9.46 | 0.18 |
| IL_8 | 1808.97 | 1267.89 | 0.02 |
| IP_10 | 122.16 | 74.67 | 0.19 |
| SLPI | 434475.2 | 196406.5 | 0.34 |
| TNFA | 0.92 | 0.92 | 1.00 |
| MIP_1a | 20.54 | 20.76 | 0.50 |
| IL1RA/IL1A | 1877.94 | 762.59 | 0.02 |
| TFV IVR |  |  |  |
| RANTES | 7.51 | 2.22 | 0.75 |
| BD2 | 46903.43 | 119891.2 | 0.15 |
| GMCSF | 1.02 | 0.85 | 0.21 |
| IL_10 | 1.37 | 0.68 | 0.27 |
| IL_1A | 224.71 | 413.99 | 0.04 |
| IL_1RA | 194882 | 125473.1 | 0.12 |
| IL_6 | 57.89 | 17.77 | 0.12 |
| IL_8 | 3298.65 | 1621.65 | 0.26 |
| IP_10 | 172.03 | 82.36 | < 0.01 |
| SLPI | 143263.3 | 165659.9 | 0.26 |
| TNFA | 2.17 | 0.74 | 0.10 |
| MIP_1a | 39.57 | 17.77 | 0.73 |
| IL1RA/IL1A | 1007.77 | 434.8 | 0.01 |
| TFVLNG IVR |  |  |  |
| RANTES | 6.13 | 4.19 | 0.28 |
| BD2 | 37773.61 | 123019.3 | 0.10 |
| GMCSF | 1.19 | 0.93 | 1.00 |
| IL_10 | 0.89 | 1.01 | 0.42 |
| IL_1A | 210.26 | 441.55 | 0.12 |
| IL_1RA | 155254 | 141048.2 | 1.00 |
| IL_6 | 25.35 | 7.62 | 0.06 |
| IL_8 | 1433.23 | 1066.17 | 1.00 |
| IP_10 | 86.42 | 155.41 | 0.08 |
| SLPI | 204796.9 | 575986.3 | 0.50 |
| TNFA | 2.33 | 1 | 0.24 |
| MIP_1a | 39.98 | 41.23 | 1.00 |
| IL1RA/IL1A | 939.44 | 547.99 | 0.11 |
