# Supplementary material for: Randomized, placebo controlled phase I trial of safety, pharmacokinetics, pharmacodynamics and acceptability of tenofovir and tenofovir plus levonorgestrel vaginal rings in women
Source: PLoS One. 2018 Jun 28;13(6):e0199778. doi: 10.1371/journal.pone.0199778 (PMC6023238; doi:10.1371/journal.pone.0199778)
Supplement: S2 Table — (DOCX) [file pone.0199778.s002.docx]

Supplemental Table 2: TFV and LNG Pharmacokinetics Summary Table

| PK Variable | TFV in Plasma (ng/mL) | | | LNG in Plasma (pg/mL) | | |
| --- | --- | --- | --- | --- | --- | --- |
|  | N | Median | Interquartile Range 25 – 75% | N | Median | Interquartile Range 25 – 75% |
| C24hrs | 40 | 0.5 | 0.3 – 0.8 | 20 | 588.0 | 477.5 – 825.5 |
| C15 Days | 27 | 1.8 | 0.4 – 2.8 | 18 | 312.6 | 207.8 – 498.6 |
| Cmax | 40 | 2.9 | 1.9 – 3.8 | 20 | 613.5 | 496.0 – 830.0 |
| Tmax (days) | 39 | 13.0 | 0.9 – 15.0 | 20 | 1.0 | 1.0 – 1.0 |
| AUC 0 – 24 hours | 40 | 0.3 | 0.2 – 0.4 | 20 | 508.9 | 387.3 – 700.1 |
| AUC 0 – 15 days | 27 | 26.8 | 17.5 – 33.6 | 18 | 6638.9 | 5596.4 – 8192.6 |
