# Supplementary material for: Randomized, placebo controlled phase I trial of safety, pharmacokinetics, pharmacodynamics and acceptability of tenofovir and tenofovir plus levonorgestrel vaginal rings in women
Source: PLoS One. 2018 Jun 28;13(6):e0199778. doi: 10.1371/journal.pone.0199778 (PMC6023238; doi:10.1371/journal.pone.0199778)
Supplement: S1 Fig — (DOC) [file pone.0199778.s003.doc]

**
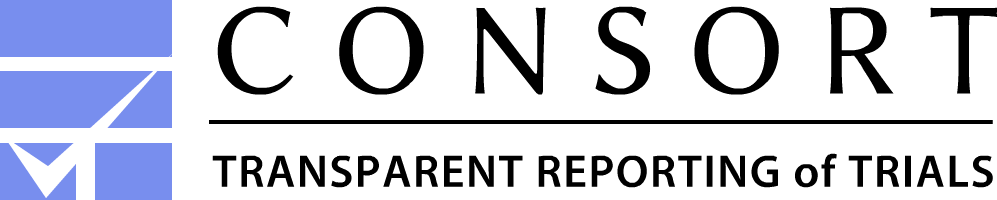
**

**CONSORT 2010 Flow Diagram**

**Allocation**

**Analysis**

**Follow-Up**

**Enrollment**

Assessed for eligibility (n = 86)

Excluded (n = 32)

♦  Not meeting inclusion criteria (n = 30)

♦  Declined to participate (n = 0 )

♦  Other reasons (n = 2)

Analysed (n= 50 )
♦ Excluded from analysis (n = 0)

Lost to follow-up (n =0 )

Discontinued intervention (TFV IVR) (n = 1 ) (Scheduling conflict/personal)

Allocated to intervention (TFV or TFVLNG IVR) (n = 41)

♦ Received allocated intervention (n = 41)

♦ Did not receive allocated intervention (n = 0)

Lost to follow-up (give reasons) (n= 0 )

Discontinued intervention (n = 0 )

Allocated to control (Placebo IVR) (n = 10)

♦ Received allocated intervention (n= 10 )

♦ Did not receive allocated intervention (n = 0)

Analysed (n=10 )
♦ Excluded from analysis (n= 0)

Randomized (n = 51)
