# Supplementary material for: Randomized, placebo controlled phase I trial of safety, pharmacokinetics, pharmacodynamics and acceptability of tenofovir and tenofovir plus levonorgestrel vaginal rings in women
Source: PLoS One. 2018 Jun 28;13(6):e0199778. doi: 10.1371/journal.pone.0199778 (PMC6023238; doi:10.1371/journal.pone.0199778)
Supplement: S1 Protocol — (PDF) [file pone.0199778.s005.pdf]

CONRAD

Tenofovir/Levonorgestrel Intravaginal Ring

Tenofovir Intravaginal Ring

Phase I One-Month Safety, Pharmacokinetic, Pharmacodynamic, and Acceptability Study of Intravaginal Rings Releasing Tenofovir and Levonorgestrel or Tenofovir Alone

IND #118,510 (tenofovir/levonorgestrel intravaginal ring)

IND #116,797 (tenofovir intravaginal ring)

PROTOCOL A13-128

27 May 2015

VERSION 4.0

Prepared By:
CONRAD
1911 Fort Myer Drive
Suite 900
Arlington VA 22209
Phone: 703-524-4744

INVESTIGATOR'S AGREEMENT

Phase I One-Month Safety, Pharmacokinetic, Pharmacodynamic, and Acceptability Study of Intravaginal Rings Releasing Tenofovir and Levonorgestrel or Tenofovir Alone

Protocol A13-128

27 May 2015

VERSION 4.0

This document may contain confidential information. It is understood that persons to whom this information is disclosed will not disclose it further without permission from CONRAD, unless such information is published or otherwise becomes public knowledge.

I have received and read the Investigator's Brochure for the Tenofovir / Levonorgestrel Intravaginal Ring as well as the Investigator's Brochure for the Tenofovir Intravaginal Ring. I have read Protocol A13-128 and agree to conduct the study as outlined. I will comply with all requirements regarding the obligations of clinical investigators as fully outlined in 21 Code of Federal Regulations (CFR) Parts 50, 56, and 312.60 and in the Statement of Investigator (1572), which I have also signed. I will ensure that all associates, colleagues, and employees assisting in the conduct of this study are informed about the obligations incurred by their contribution to the study. I agree to maintain the confidentiality of all information received or developed in connection with this protocol.

____

Printed Name of Investigator

____

Signature of Investigator

____

Date

PROCEDURES IN CASE OF EMERGENCY

Table 1: Emergency Contact Information

Role in Study	Name	Email and Telephone number
Clinical Study Leader	Christine Mauck, M.D.	cmauck@conrad.org<br>703-276-3912
Medical Director/SAE Reporting	Jill Schwartz, M.D.	jschwartz@conrad.org<br>703-276-3913
Director, Clinical Research	Kim Linton, M.H.S.	klinton@conrad.org<br>703-276-4021

2. SYNOPSIS

Name of Sponsor:

CONRAD
1911 Fort Myer Drive
Suite 900
Arlington VA 22209

Name of Investigational Product:

Tenofovir/Levonorgestrel or Tenofovir Intravaginal Ring

Name of Active Ingredient:

Tenofovir (TFV), Levonorgestrel (LNG)

Title of Study:

Phase I One-Month Safety, Pharmacokinetic, Pharmacodynamic, and Acceptability Study of Intravaginal Rings Releasing Tenofovir and Levonorgestrel or Tenofovir Alone, Protocol A13-128

Principal Investigators and Study Centers:

- Andrea Thurman, M.D., Clinical Research Center, Eastern Virginia Medical School, Norfolk, VA, USA
- Vivian Brache, Lic., PROFAMILIA, Santo Domingo, Dominican Republic

**Studied period:**	**Phase of development:**
Estimated date first patient enrolled: 31August 2014<br>Estimated date last patient completed: 31 July 2015	Phase I

Objectives:

Primary objective:

- Evaluate genital and systemic safety of the TFV/LNG intravaginal ring (IVR), TFV-only IVR, and placebo IVR

Secondary objectives:

- Evaluate pharmacokinetics (PK) of TFV in users of the TFV/LNG and TFV-only IVRs
- Evaluate PK of LNG in users of the TFV/LNG IVR

Tertiary objectives:

- Evaluate pharmacodynamic (PD) surrogates of contraceptive efficacy of LNG in blood and cervical mucus in users of the TFV/LNG IVR
- Evaluate acceptability of the TFV/LNG IVR, TFV-only IVR, and placebo IVR

Exploratory objectives:

- Evaluate PD surrogates of microbicidal efficacy of TFV in users of the TFV/LNG and TFV-only IVRs
- Evaluate PD endpoints of LNG in the endometrium in users of the TFV/LNG IVR
- Evaluate genital safety using exploratory markers in users of the TFV/LNG IVR, TFV IVR, and placebo IVR
- Assess the correlation of TFV levels between less-invasive swabs and more invasive PK measures

- Develop objective markers of IVR adherence

Endpoints:

Primary endpoints: Genital and Systemic Safety

- Treatment-emergent adverse events, as reported by the participant
- Changes in serum chemistries, lipids, and complete blood count (CBC)
- Development of cervicovaginal ulcerations, abrasions, edema, and other findings as assessed by naked eye and colposcopic visualization of the cervicovaginal epithelium
- Changes in soluble markers of innate mucosal immunity and inflammatory response in cervicovaginal lavage (CVL) fluid, e.g., interleukins (IL)-1β, IL-6, IL-8, tumor necrosis factor alpha (TNF-α), human β defensins 1 and 2, secretory leukocyte protease inhibitor (SLPI), human defensin 5, IL-1 receptor antagonist (IL-1 RA)
- Changes in HIV-1 target immune cell phenotype (e.g., CD45, CD68, CD4, and CD1a) and HIV-1 activation/proliferation markers (e.g., CD38, CCR5, HLA-DR, Ki67) in cervicovaginal tissue (biopsy)
- Changes in hydrogen peroxide-secreting Lactobacilli concentration and other endogenous vaginal bacteria by semi-quantitative vaginal culture and/or unculturable 16S RNA bacteria by quantitative PCR
- Changes in Nugent Score

Secondary endpoints: Pharmacokinetics of LNG and TFV

- Concentrations of LNG, TFV, and tenofovir diphosphate (TFV-DP) as follows:
  - TFV concentrations in plasma, cervicovaginal fluid (aspirate and swab), and genital tissue
  - TFV-DP concentrations in peripheral blood mononuclear cells (PBMCs) and genital tissue
  - LNG in blood, vaginal secretions (swabs) and cervical mucus
  - Steroid hormone binding globulin (SHBG) in blood
- Weight of returned IVRs
- Amount of drug remaining in returned IVRs

Tertiary endpoints: Pharmacodynamics of LNG

- Surrogates of contraceptive efficacy:
  - Cervical mucus assessment
    - Cervical mucus quality (score of >10)
    - Sperm migration on the Simplified Slide test
  - Ovulation by serum progesterone (P4)
  - Effect on follicular development by serum estradiol concentration

Tertiary endpoints: Acceptability

- Discontinuations
- Expulsions
- Removals
- Visible changes (such as discoloration) documented on photographs of returned IVRs
- Responses to key questions on acceptability questionnaire

Exploratory endpoints: Pharmacodynamic surrogates of TFV

- Anti-HSV-2 and anti-HIV-1 activities in cervicovaginal fluid (CVF)

- TFV anti-HIV efficacy in cervicovaginal tissues (Eastern Virginia Medical School [EVMS] only):
  - Comparison of cervicovaginal tissue permissiveness to ex vivo infection with HIV-1 BaL between the control cycle and treatment cycle

Exploratory endpoints: Pharmacodynamic surrogates of LNG in endometrium

- Findings on endometrial biopsy (EVMS site only):
  - Histology (e.g., proliferative, secretory, atrophic)
  - Markers of endometrial function
- Endometrial thickness as assessed by transvaginal ultrasound

Exploratory endpoints: Exploratory markers of genital safety (EVMS only)

- Changes in cervicovaginal epithelial histology (thickness and number of cell layers) and epithelial integrity, as measured by immunohistochemistry (IHC) of epithelial junction proteins in cervicovaginal tissue (biopsy)
- Changes in markers of mucosal alteration and inflammation (e.g., expression of COX-2) in cervicovaginal and endometrial tissue (biopsy)
- Microbial growth on swabs obtained from returned IVRs and microbial levels in returned IVRs

Exploratory endpoint: Correlation between less-invasive and more-invasive PK measures

- Level of association of TFV levels between less-invasive swabs and the more invasive biopsies, and possibly between swabs and aspirates

Exploratory endpoint: Develop objective markers of IVR adherence

- Characterization of returned IVRs for physicochemical properties and potential chemical and/or biological measures of adherence

Study Design:

This will be an outpatient, randomized, partially blinded, placebo-controlled, parallel study. Eligible participants will be randomized in a 2:2:1 ratio (TFV/LNG:TFV-only:placebo) to use 1 of the 3 IVRs for approximately one month.

All women will undergo a screening visit to detect the presence of exclusion factors. Blood will be drawn for P4 at Visit 2, scheduled on Day 21 of the participant's menstrual cycle, to confirm ovulation in the control cycle. Ovulation will be considered confirmed by a P4 level of ≥3.0 ng/ml. Once ovulation is confirmed, participants will undergo baseline sampling at Visit 3.

Participants will initiate use of the IVR in the clinic on day 7 of their next menstrual cycle (Visit 4). Participants will undergo colposcopy and collection of blood and genital samples for safety, PK, and PD before IVR insertion. After observed IVR insertion, assessment of PK in blood will be done at 1, 2, 4, and 8 hours for all participants and in CVF at a randomly selected time point 1, 2, 4, or 8 hours (Visit 4 post-insertion).

Visit 5 will take place 24 hours after insertion and will include assessment of PK in blood, CVF, and tissue. Colposcopy will be performed.

Starting on day 10 of their cycle, participants will use an ovulation predictor kit to determine the day of ovulation, at which point their cervical mucus will be assessed for PK and PD (Visit 6). They will undergo colposcopy and collection of blood and CVF for PK.

Visit 7 will occur 8-10 days after Visit 6. Participants will complete an acceptability questionnaire;

undergo colposcopy and collection of blood and genital samples for safety, PK, and PD. The IVR will be removed prior to genital biopsies. Visit 8 will take place 24 hours after IVR removal, and will include assessment of PK in blood and cervicovaginal fluid, and PK in cervical mucus.<br><br>Assessment of PK in tissue after IVR removal will occur at a randomly selected time point (24 hours [Visit 8] or 72 hours [Visit 9]).<br><br>The site will contact the participant 1-2 weeks after final genital sampling to ask about adverse events (AEs) experienced and medications taken since the last visit. The participant will then be exited from the study, unless she has symptoms that require follow-up. There will be approximately 8 or 9 visits and one scheduled follow-up call/contact over approximately 2½ months.<br><br>The TFV-only IVR is visually different from the other two IVRs which are similar and difficult to distinguish from each other. The participants will not be told which IVR they have received and the lab staff will be blinded to the extent possible.<br><br>Participants will be given a guidebook in which to record AEs and concomitant medications. All participants will be instructed not to remove the IVR but, if removal becomes necessary for some reason, or if the IVR is expelled, to record the time of expulsion/removal and reinsertion in their guidebook. Menses, bleeding/spotting and whether intercourse took place will also be recorded. Participants will abstain from vaginal intercourse from Visit 1 (48 hours prior to Visit 3 if protected from pregnancy via sterilization of either partner) until the sixth day after the last study visit, and from all other vaginal activity, anal intercourse, and use of all non-study vaginal products starting 48 hours before Visit 3 until the sixth day after the last study visit.<br><br>An interim analysis may be conducted after at least 15 participants (both sites combined) have completed the study in order to evaluate drug-release parameters to help inform product development.
**Number of patients (planned):**<br><br>Approximately 100 women may be consented and undergo assessment procedures in order to have at least 50 women complete the study. Women will be randomized in a 2:2:1 ratio (TFV/LNG:TFV-only: placebo).
**Diagnosis and main criteria for inclusion:**<br><br>Healthy, non-pregnant, ovulatory, HIV-uninfected women aged 18 to 45 with a body mass index (BMI) less than 30 kg/m^2, menstrual cycles of 26-35 days by participant report and willing to abstain from vaginal activity and intercourse during the study. Women will be protected from pregnancy by sterilization of either partner or by being willing to remain abstinent from vaginal intercourse from Visit 1 until the sixth day after the last study visit. (Women who have been sterilized or who have a sterilized partner will abstain from intercourse from 48 hours prior to Visit 3 until the sixth day after the last study visit.) All randomized participants will be included in the analysis.
**Investigational product, dosage and mode of administration:**<br>IVR releasing either 8-10 mg of TFV and 20 µg of LNG per day, or 8-10 mg of TFV only per day
**Duration of treatment:**<br>Approximately one month, depending on cycle length
**Reference therapy, dosage and mode of administration:**

Placebo IVR
**Criteria for evaluation:**<br>***Safety:*** It is expected that there will be no clinically significant difference in safety endpoints between the placebo group and either of the treatment groups.
**Statistical methods:**<br>*Sample Size Justification:* The sample size is based on feasibility considerations (e.g., cost and time) and not on statistical criteria.<br>*Evaluation of Objectives:* Study objectives will be evaluated by clinical review of descriptive summaries and graphical displays. To supplement clinical judgments, comparisons on selected continuous endpoints between each active group vs. placebo (or, as relevant, between the TFV/LNG ring vs. the TFV-alone ring groups) may be estimated (with 95% confidence intervals) from statistical models, with terms for treatment and center as fixed effects and time of measurement as a repeated measure. As this study is descriptive in nature, p-values and confidence intervals around estimates of treatment differences will not be adjusted for multiple analyses. It is acknowledged that the trial is not necessarily powered to test hypotheses regarding group differences. Inferences based on statistical significance (or lack thereof) will be made cautiously.<br>*Study Populations:* The Enrolled Population consists of all participants who undergo genital sampling at Visit 3; reasons why an enrolled participant was not randomized or did not use a study IVR will be listed. The Randomized Population (RP) (those who were randomized and inserted the study IVR at Visit 4) will be the primary analysis population for baseline characteristics and study disposition. The Treated Population (TP), the subset of RP who provided at least some post-insertion safety, PK, or PD data, will be the primary analysis population for all remaining analysis. Some analysis (e.g., drug concentrations) may be repeated on the Completer Population (the subset of TP considered adherent and who completed the study) to evaluate changes across time within consistent groups. For all analysis, treatment groups are defined on the basis of treatment assigned. A flowchart showing the disposition of all screened participants will be provided.<br>*Interim Analysis:* An interim analysis may be conducted after at least 15 participants (both sites combined) have completed the study in order to evaluate drug-release parameters to help inform product development. The analysis will consist of descriptive statistics and graphical displays of drug concentrations by treatment group and sampling time point.<br>*Evaluation of the Primary Objective:* Only treatment-emergent AEs (those with onset on or after insertion of IVR) will be included in the analysis; other AEs will be listed. AEs will be summarized separately by treatment group based on MedDRA Preferred Term and System/Organ Class. The number of subjects reporting at least one episode of a given AE category (Preferred Term) as well as number of episodes will be summarized and listed. Serious AEs will be identified. All other genital safety endpoints will be summarized separately by treatment group and time of measurement. Shift tables will describe changes from baseline or, when collected, between immediate pre- vs. post IVR insertion. Non-iatrogenic colposcopy findings will be described based on characteristics at the first observation, though characteristics at all observations will be listed. Ulcerations, abrasions, etc. will be based on diagnoses from colposcopists.<br>*Evaluation of Secondary Objectives:* Plasma C_{max}, t_{max} and area under the curve ($AUC_{0\text{-}24h}$) (ln/linear trapezoidal method) from the intensive sampling immediately post insertion will be estimated for

participants in active treatment groups from non-compartmental analysis using Phoenix/WinNonLin v6.1 (Pharsight, Inc., Mountain View CA) or higher. Group estimates for plasma C_{max} and t_{max} will be based on the mean and median, respectively. Time x concentration curves associated with ring insertion, and time x concentration (decay) curves associated with ring removal will be graphed individually by participant as well as pooled by treatment group. Descriptive statistics and graphical displays, as needed, will also be presented by group and sampling time point for all concentrations.

Evaluation of Tertiary/Exploratory Endpoints: Acceptability will be assessed clinically from: (a) early discontinuation for reasons related to study IVR; (b) proportion of women in each IVR group who stop using ring prior to Visit 7; (c) proportion of women in each group who experience spontaneous ring expulsions; and (d) responses to the acceptability questionnaire. Visible changes to returned rings will be described. The evaluation of PD endpoints will be described in the statistical analysis plan (SAP).

3. TABLE OF CONTENTS, LIST OF TABLES, AND LIST OF FIGURES

TABLE OF CONTENTS

1. TITLE PAGE 1
2. SYNOPSIS 4
3. TABLE OF CONTENTS, LIST OF TABLES, AND LIST OF FIGURES 10
4. LIST OF ABBREVIATIONS AND DEFINITIONS OF TERMS 17
5. INTRODUCTION 20
6. TRIAL PURPOSE, OBJECTIVES AND ENDPOINTS 22
6.1. Purpose 22
6.2. Primary Objective 22
6.3. Secondary Objectives 22
6.4. Tertiary Objectives 22
6.5. Exploratory Objectives 22
6.6. Endpoints 22
6.6.1. Primary endpoints: Genital and Systemic Safety 22
6.6.2. Secondary endpoints: Pharmacokinetics of LNG and TFV 23
6.6.3. Tertiary endpoints: Pharmacodynamics of LNG 23
6.6.4. Tertiary endpoints: Acceptability 23
6.6.5. Exploratory endpoints: Pharmacodynamic surrogates of TFV 23
6.6.6. Exploratory endpoints: Pharmacodynamic surrogates of LNG in endometrium 24
6.6.7. Exploratory endpoints: Exploratory markers of genital safety (EVMS only) 24
6.6.8. Exploratory endpoint: Correlation between less-invasive and more-invasive PK measures 24
6.6.9. Exploratory endpoint: Develop objective markers of IVR adherence 24
7. INVESTIGATIONAL PLAN 25
7.1. Overall Study Design 25
7.2. Anticipated Length of Study 30
7.3. Number of Subjects 30
7.4. Treatment Assignment 30

7.5. Criteria for Study Termination 30
8. SELECTION AND WITHDRAWAL OF PARTICIPANTS....31
8.1. Inclusion Criteria 31
8.2. Exclusion Criteria 32
8.3. Participant Withdrawal 33
8.3.1. Withdrawal Criteria 33
8.3.2. Replacement 33
8.3.3. Follow-up....34
9. STUDY PROCEDURES 35
9.1. Visit 1: Screening/Enrollment 35
9.2. Visit 2: Confirmation of Ovulation....36
9.3. Visit 3: Baseline Sample Collection 37
9.4. Visit 4: Randomization, Initiation of IVR Use 38
9.5. Visit 5: 24-hour Sample Collection, IVR Day 2....40
9.6. Visit 6: Ovulation Sample Collection....40
9.7. Visit 7: IVR Removal and Sample Collection....42
9.8. Visit 8: 24 hour Post-IVR Sample Collection 43
9.9. Visit 9: 72-hour Post-IVR Sample Collection....43
9.10. Follow-up Call/Contact 44
9.11. Unscheduled Visits 44
10. TREATMENT OF PARTICIPANTS....46
10.1. Study Drug....46
10.2. Concomitant Medications....46
10.3. Treatment Compliance....47
10.4. Randomization and Blinding 47
11. STUDY DRUG MATERIALS AND MANAGEMENT 49
11.1. Description of Study Drug....49
11.2. Study Drug Packaging and Labeling 49
11.3. Study Drug Storage....49
11.4. Study Drug Preparation 50
11.5. Administration....50
11.6. Study Drug Accountability....50

11.7. Study Drug Handling and Disposal 50
12. ASSESSMENT OF ENDPOINTS 51
12.1. Assessment of Pharmacokinetic and Pharmacodynamics 52
12.1.1. Pharmacokinetics: Blood Sample Collection 52
12.1.2. Pharmacokinetics: Genital Sample Collection 52
12.1.3. Pharmacodynamics: Blood Sample Collection 53
12.1.4. Pharmacodynamics: Genital Sample Collection 53
12.2. Assessment of Safety 54
12.2.1. Medical History 54
12.2.2. Physical and Pelvic Examination...54
12.2.3. Genital Assessments 56
12.2.3.1. Hematology and Blood Chemistry 56
12.2.3.2. Pregnancy Screen...56
13. ADVERSE AND SERIOUS ADVERSE EVENTS...57
13.1. Definition of Adverse Events (AE) 57
13.1.1. Suspected Adverse Reaction...57
13.1.2. Adverse Reaction...57
13.1.3. Serious Adverse Event (SAE) or Serious Suspected Adverse Reaction 57
13.1.4. Unexpected Adverse Event or Unexpected Suspected Adverse Reaction 58
13.2. Relationship to Study Drug 58
13.3. Recording and Grading Adverse Events for Severity...59
13.4. Reporting Serious Adverse Events 60
13.5. Temporary Product Hold/Permanent Discontinuation in Response to Adverse Events 61
13.5.1. Grade 1 or 2 61
13.5.2. Grade 3 and 4..61
14. STATISTICS 62
14.1. Sample Size Justification...62
14.2. Analysis Plan 62
14.3. General Statistical Issues 62
14.4. Analysis Populations 63
14.5. Interim Analysis..63

14.6. Evaluation of Primary Objectives 64
14.7. Evaluation of Secondary Endpoints 65
14.7.1. Pharmacokinetic Endpoints 65
14.8. Evaluation of Tertiary and Exploratory Endpoints 65
15. MANAGEMENT OF INTERCURRENT EVENTS 66
15.1. Loss to Follow-up 66
15.2. Protocol Adherence 66
15.3. Protocol Violations 66
15.4. Modification of Protocol 66
16. DIRECT ACCESS TO SOURCE DATA/DOCUMENTS 67
16.1. Study Monitoring 67
16.2. Audits and Inspections 67
16.3. Institutional Review Board (IRB) 68
17. QUALITY CONTROL AND QUALITY ASSURANCE 69
18. ETHICS 70
18.1. Ethics Review 70
18.2. Ethical Conduct of the Study 70
18.3. Written Informed Consent 70
18.3.1. Procedure for Obtaining Informed Consent 70
18.3.2. Subject Confidentiality 71
19. DATA HANDLING AND RECORDKEEPING 72
19.1. Method of Data Capture 72
19.2. Inspection of Records 72
19.3. Retention of Records 72
20. INVESTIGATOR RESPONSIBILITIES 73
20.1. Prior to Starting Study 73
20.1.1. Signing of Investigator’s Agreement and Amendments 73
20.1.2. Forms and Records 73
20.2. During the Study: Forms and Records 74
20.3. During the Study: Progress Reports 74
21. PUBLICATION POLICY 76
22. LIST OF REFERENCES 77

LIST OF TABLES

Table 1: Emergency Contact Information....3
Table 2: Abbreviations and Specialist Terms....17
Table 3: Study Design and Schedule of Assessments....27
Table 4: Relationship of Study Visits to IVR Day and Cycle Day....30
Table 5: Investigational Product....46
Table 6: Types of Specimens to be Collected, Purpose and Sequence of Collection....51
Table 7: Pharmacokinetics: Blood Samples, Concentrations....52
Table 8: Pharmacokinetics: Genital Sample, Concentrations....52
Table 9: Pharmacodynamics: Blood Sample Collection....53
Table 10: Pharmacodynamics: Genital Samples....53
Table 11: Safety: Physical and Pelvic Examination....54
Table 12: Safety Management Plan for Local Toxicity Observed on Pelvic Exam, including Colposcopy....55
Table 13: Safety: Genital Assessments....56

LIST OF FIGURES

Figure 1: Study Drug Label..49
Figure 2: Grading the Severity of Adverse Events 59

4. LIST OF ABBREVIATIONS AND DEFINITIONS OF TERMS

The following abbreviations and specialist terms are used in this study protocol.

Table 2: Abbreviations and Specialist Terms

**Abbreviation or Specialist Term**	**Explanation**
AE	Adverse event
AIDS	Acquired Immunodeficiency Syndrome
API	Active pharmaceutical ingredient
AUC	Area under the curve
BLQ	Below level of quantification
BMI	Body mass index
BV	Bacterial vaginosis
CAPRISA	Centre for AIDS Programme of Research in South Africa
CBC	Complete blood count
CFR	Code of Federal Regulations
CLIA	Clinical Laboratory Improvement Act
CRF	Case report form
CT	Concomitant therapy or *Chlamydia trachomatis*
CV	Curriculum vitae
CVF	Cervicovaginal fluid
CVL	Cervicovaginal lavage
DR	Dominican Republic
EVMS	Eastern Virginia Medical School
FDA	U.S. Food and Drug Administration
FP	Family planning
FT-IR	Fourier transform infrared spectroscopy (FT-IR)
FWA	Federal-wide assurance
GC	*Neisseria gonorrhea*
GCP	Good Clinical Practice
GMP	Good Manufacturing Practice
HBsAg	Hepatitis B surface antigen
HIV	Human Immunodeficiency Virus
HSV-2	Herpes simplex virus – 2

Abbreviation or Specialist Term	Explanation
ICH	International Conference on Harmonization
IL	Interleukins
IRB	Institutional Review Board
IUD	Intrauterine device
IVR	Intravaginal ring
LDC	Less developed countries
LLOQ	Lower limit of quantification
LNG	Levonorgestrel
MPA	Multiple Project Assurance
NIH	National Institutes of Health
NSAID	Non-steroidal anti-inflammatory drug
OC	Oral contraceptive
P4	Serum progesterone
PBMC	Peripheral blood mononuclear cell
PD	Pharmacodynamics
PI	Principal Investigator
PK	Pharmacokinetics
RA	Receptor antagonist
RH	Reproductive health
RP	Randomized population
RTI	Reverse transcriptase inhibitor
SAE	Serious adverse event
SAP	Statistical analysis plan
SHBG	Steroid hormone binding globulin
SLPI	Secretory leukocyte protease inhibitor
STI	Sexually transmitted infection
TDF	Tenofovir disoproxil fumarate
TFV	Tenofovir
TFV-DP	Tenofovir diphosphate
TNF-α	Tumor necrosis factor alpha
TP	Treated population
UTI	Urinary tract infection

Abbreviation or Specialist Term	Explanation
WHO	World Health Organization

5. INTRODUCTION

Almost half of all pregnancies worldwide, estimated to be over 100 million annually, are unintended .[1,2] Despite the existence of a variety of effective contraceptives, discontinuation and non-use remain high, primarily due to side effects, cost, inconvenient dosing schedules, limited access to prescription products and/or poor acceptance of the method by the male partner, resulting in an unacceptably high rate of unintended or mistimed pregnancies. There is a compelling need for innovative and acceptable contraceptives to fill gaps in the existing method mix available worldwide. Statistics clearly show an unmet need for highly effective contraception, especially in less developed countries (LDCs), where 99% of maternal deaths occur. [1,3] Not surprisingly, these countries, especially those of sub-Saharan Africa and south Asia, are also at the core of the acquired immunodeficiency syndrome (AIDS) epidemic. [4]

Over 33 million people worldwide are infected with human immunodeficiency virus type 1 (HIV-1) and 22.4 million live in sub-Saharan Africa. [3] Women increasingly bear the burden of the HIV-1 pandemic, with more than 60% of new infections occurring in women in sub-Saharan Africa.[3] Most women acquire HIV-1 through heterosexual contact with an infected male partner, as they are unable to negotiate condom use. Poverty, malnutrition, lack of education, and gender inequality fuel both unplanned pregnancies and HIV-1 transmission. There are a significant number of women, especially in LDCs, needing protection against sexually transmitted infections (STIs), in particular HIV-1/AIDS. These women also need long-term, highly effective contraceptive methods to provide optimal birth spacing and family size. Highly effective contraceptives (e.g., sterilization, intrauterine devices, hormonal contraception) typically provide little or no protection against STIs, while barrier methods (e.g., male or female condoms) have unacceptably high contraceptive failure rates with typical use.

A major breakthrough in HIV-1 prevention was recently achieved with the announcement of the Centre for the AIDS Programme of Research in South Africa (CAPRISA) 004 trial results at the 2010 International AIDS Conference. [5] The first randomized, double blind, placebo-controlled trial of 1% TFV vaginal gel found a 39% overall reduction in HIV-1 incidence (Intent-to-Treat analysis) and a 54% reduction among women with high adherence to gel use. [5]

There is an urgent need to expand upon the successes of recent advances in HIV-1 prevention and past novel contraceptive delivery systems to further integrate the priorities of family planning (FP) and reproductive health (RH) especially in highly vulnerable populations in LDCs. CONRAD plans to meet this need by developing a "dual-protection" product, providing both contraceptive and microbicidal activity, that is safe, highly effective, acceptable and low-cost. CONRAD has developed an IVR which releases LNG and TFV for at least 90 days and has completed pre-clinical product development testing of IVRs and of TFV gel. The purpose of this phase 1 trial is to study the safety, PK, PD, and acceptability of the TFV/LNG and TFV-alone IVR in women not at risk of pregnancy.

LNG has an established track record of safety and efficacy and is, arguably, the best progestin to be incorporated in a controlled-release device, due to its low molecular weight, physical stability, and steady micro-dose release rate from an IVR. [6] The contraceptive efficacy of a 20 µg/day LNG IVR has been clearly demonstrated by two clinical studies.[7,8] The first was carried out by the World Health Organization (WHO) in 1005 women and yielded a life table pregnancy rate (intrauterine) with the IVR in situ of 3.6 per 100 women (95% CI 2.2-5.0) and of ectopic pregnancy 0.2% (one woman). [7] This pregnancy rate was felt to compare favorably with a combined oral contraceptive (OC) tested by WHO

and was less than half the pregnancy rate of a progestin-only OC tested by WHO.[9,10] Likewise, the ectopic pregnancy rate was within the range of ectopic pregnancy rates seen with copper intrauterine devices (IUDs) and progestin-only OCs. The second study was carried out in the United Kingdom among 1710 women and yielded a 1-year total pregnancy rate of 5.1% (CI 3.6-6.6%) and a 2-year rate of 6.5% (CI 4.4-8.6%).[8] The corresponding method failure rates were 4.0% and 1.2%. This was felt to be comparable to pregnancy rates seen with progestin-only OCs.

Erythematous lesions were found in some women, mostly at one center. A placebo IVR was redesigned with a smaller cross-sectional diameter (6.0 vs. 9.5 mm) and greater flexibility, requiring only 1.3 vs 6.4 newtons to compress it through 20 mm. A 6-month multi-center randomized study was done to compare this IVR (n=112) with a control group (n=54) not using an IVR.[11] No clinically significant findings were found in any participant. This difference in results could be attributed to changes in the IVR, the absence of LNG, or both. The CONRAD IVR was designed to be similar to NuvaRing®, and is within the range of stiffnesses observed with marketed rings (e.g., slightly stiffer than NuvaRing, slightly less stiff than Estring®).

TFV is a nucleotide reverse transcriptase inhibitor (RTI) orally administered as a bioavailable prodrug, tenofovir disoproxil fumarate (TDF; Viread®). TFV is an important component of today's anti-HIV therapeutic drug armamentarium and, with over 3.5 million patient-years of use, has been demonstrated to be safe and effective, with low risk of developing resistance. Safety, PK and acceptability studies of 1.0% TFV gel have demonstrated its suitability as a microbicide. However, TFV microbicide gel has some potential disadvantages, in terms of dosing regimen. Specifically, participants enrolled in CAPRISA were instructed to insert the first dose of TFV gel vaginally within the 12 hours prior to intercourse, as soon as possible within the 12 hours after intercourse and to use no more than 2 doses in one 24 hour period.[5] We believe that an IVR, which would eliminate the need for pre-coital and post-coital administration, would enhance adherence and assure optimal genital tract tissue levels of drug prior to virus exposure. Preclinical animal studies have shown similar TFV tissue concentrations after TFV 1% vaginal gel and TFV IVR applications.

The goals of this project are to: assess the genital and systemic safety of IVRs delivering LNG and TFV, and TFV alone; assess the PK of LNG in blood and cervical mucus and PK of TFV and TFV-DP in blood, cervicovaginal secretions, and tissue in users of these IVRs; assess PD of LNG in blood and cervical mucus and PD of TFV in cervicovaginal secretions, the endometrium, and cervicovaginal tissue; and assess acceptability of these IVRs.

6. TRIAL PURPOSE, OBJECTIVES AND ENDPOINTS

6.1. Purpose

The purpose of this trial is to assess the safety, PK/PD, and acceptability of the TFV/LNG IVR and TFV-only IVR.

6.2. Primary Objective

- Evaluate genital and systemic safety of the TFV/LNG IVR, TFV-only IVR, and placebo IVR

6.3. Secondary Objectives

- Evaluate PK of TFV in users of the TFV/LNG and TFV-only IVRs
- Evaluate PK of LNG in users of the TFV/LNG IVR

6.4. Tertiary Objectives

- Evaluate PD surrogates of contraceptive efficacy of LNG in blood and cervical mucus in users of the TFV/LNG IVR
- Evaluate acceptability of the TFV/LNG IVR, TFV-only IVR, and the placebo IVR

6.5. Exploratory Objectives

- Evaluate PD surrogates of microbicidal efficacy of TFV in users of the TFV/LNG and TFV-only IVRs
- Evaluate PD endpoints of LNG in the endometrium in users of the TFV/LNG IVR
- Evaluate genital safety using exploratory markers in users of the TFV/LNG IVR, TFV IVR, and placebo IVR
- Assess the correlation of TFV levels between less-invasive swabs and more invasive PK measures
- Develop objective markers of IVR adherence

6.6. Endpoints

6.6.1. Primary endpoints: Genital and Systemic Safety

- Treatment-emergent AEs, as reported by the participant
- Changes in serum chemistries, lipids, and CBC
- Development of cervicovaginal ulcerations, abrasions, edema, and other findings as assessed by naked eye and colposcopic visualization of the cervicovaginal epithelium
- Changes in soluble markers of innate mucosal immunity and inflammatory response in CVL fluid, e.g., IL-1β, IL-6, IL-8, TNF-α, human β defensins 1 and 2, SLPI, human defensin 5, and IL-1 RA

- Changes in HIV-1 target immune cell phenotype (e.g., CD45, CD68, CD4, and CD1a) and HIV-1 activation/proliferation markers (e.g., CD38, CCR5, HLA-DR, Ki67) in cervicovaginal tissue (biopsy)
- Changes in hydrogen peroxide-secreting Lactobacilli concentration and other endogenous vaginal bacteria by semi-quantitative vaginal culture and/or unculturable bacteria by quantitative PCR of the 16S RNA gene
- Changes in Nugent Score

6.6.2. Secondary endpoints: Pharmacokinetics of LNG and TFV

- Concentrations of LNG, TFV, and TFV-DP as follows:
  - TFV concentrations in plasma, cervicovaginal fluid (aspirate and swab), and genital tissue
  - TFV-DP concentrations in PBMCs and genital tissue
  - LNG in blood, vaginal secretions (swabs) and cervical mucus
  - SHBG in blood
- Weight of returned IVRS
- Amount of drug remaining in returned IVRs

6.6.3. Tertiary endpoints: Pharmacodynamics of LNG

- Surrogates of contraceptive efficacy:
  - Cervical mucus assessment
    - Cervical mucus quality (score of >10)
    - Sperm migration on the Simplified Slide test
  - Ovulation by P4
  - Effect on follicular development by serum estradiol concentration

6.6.4. Tertiary endpoints: Acceptability

- Discontinuations
- Expulsions
- Removals
- Visible changes (such as discoloration) documented on photographs of returned IVRs
- Responses to key questions on acceptability questionnaire

6.6.5. Exploratory endpoints: Pharmacodynamic surrogates of TFV

- Anti-HSV-2 and anti-HIV-1 activities in cervicovaginal fluid (CVF)
- TFV anti-HIV efficacy in cervicovaginal tissues (EVMS only):

    - Comparison of cervicovaginal tissue permissiveness to ex vivo infection with HIV-1 BaL between the control cycle and treatment cycle

6.6.6. Exploratory endpoints: Pharmacodynamic surrogates of LNG in endometrium

- Findings on endometrial biopsy (EVMS site only):
    - Histology (e.g., proliferative, secretory, atrophic)
    - Markers of endometrial function
- Endometrial thickness as assessed by transvaginal ultrasound

6.6.7. Exploratory endpoints: Exploratory markers of genital safety (EVMS only)

- Changes in cervicovaginal epithelial histology (thickness and number of cell layers) and epithelial integrity, as measured IHC of epithelial junction proteins in cervicovaginal tissue (biopsy)
- Changes in markers of mucosal alteration and inflammation (e.g., expression of COX-2) in cervicovaginal and endometrial tissue (biopsies)
- Microbial growth on swabs obtained from returned IVRs and microbial levels in returned IVRs

6.6.8. Exploratory endpoint: Correlation between less-invasive and more-invasive PK measures

- Level of association of TFV levels between less-invasive swabs and the more invasive biopsies, and possibly between swabs and aspirates

6.6.9. Exploratory endpoint: Develop objective markers of IVR adherence

- Characterization of returned IVRs for physicochemical properties and potential chemical and/or biological measures of adherence

7. INVESTIGATIONAL PLAN

7.1. Overall Study Design

This will be an outpatient, randomized, partially blinded, placebo-controlled, parallel study. Eligible participants will be randomized in a 2:2:1 ratio (TFV/LNG:TFV-only:placebo) to use 1 of the 3 IVRs for approximately one month.

All women will undergo a screening visit to detect the presence of exclusion factors. Blood will be drawn for P4 at Visit 2, scheduled on Day 21 of the participant's menstrual cycle, to confirm ovulation in the control cycle. Ovulation will be considered confirmed by a P4 level of ≥3.0 ng/ml. Once ovulation is confirmed, participants will undergo baseline sampling at Visit 3.

Participants will initiate use of the IVR in the clinic on day 7 of their next menstrual cycle (Visit 4). Participants will undergo colposcopy and collection of blood and genital samples for safety, PK, and PD before IVR insertion. After observed IVR insertion, assessment of PK in blood will be done at 1, 2, 4, and 8 hours for all participants and in CVF at a randomly selected time point 1, 2, 4, or 8 hours (Visit 4 post-insertion).

Visit 5 will take place 24 hours after insertion and will include assessment of PK in blood, CVF, and tissue. Colposcopy will be performed.

Starting on day 10 of their cycle, participants will use an ovulation predictor kit to determine the day of ovulation, at which point their cervical mucus will be assessed for PK and PD (Visit 6). They will undergo colposcopy and collection of blood and CVF for PK.

Visit 7 will occur 8-10 days after Visit 6. Participants will complete an acceptability questionnaire, undergo colposcopy and collection of blood and genital samples for safety, PK, and PD. The IVR will be removed prior to genital biopsies. Visit 8 will take place 24 hours after IVR removal, and will include assessment of PK in blood and cervicovaginal fluid, and PK in cervical mucus.

Assessment of PK in tissue after IVR removal will occur at a randomly selected time point (24 hours [Visit 8] or 72 hours [Visit 9]).

The site will contact the participant 1-2 weeks after final genital sampling to ask about AEs experienced and medications taken since the last visit. The participant will then be exited from the study, unless she has symptoms that require follow-up. There will be approximately 8 or 9 visits and one scheduled follow-up call/contact over approximately 2½ months.

The TFV-only IVR is visually different from the other two IVRs which are similar and difficult to distinguish from each other. The participants will not be told which IVR they have received and the lab staff will be blinded to the extent possible.

Participants will be given a guidebook in which to record AEs and concomitant medications. All participants will be instructed not to remove the IVR but, if removal becomes necessary for some reason, or if the IVR is expelled, to record the time of expulsion/removal and reinsertion in their guidebook. Menses, bleeding/spotting and whether intercourse took place will also be recorded. Participants will abstain from vaginal intercourse from Visit 1 (48 hours prior to Visit 3 if protected from pregnancy via sterilization of either partner) until the sixth day after the last study visit, and from all other vaginal

activity, anal intercourse, and use of all non-study vaginal products starting 48 hours before Visit 3 until the sixth day after the last study visit.

An interim analysis may be conducted after at least 15 participants (both sites combined) have completed the study in order to evaluate drug-release parameters to help inform product development.

The study design and schedule of assessments is summarized in Table 3.

Table 4 summarizes the relationship of study visits to day of IVR use and participant cycle day. This table assumes that participants have ovulated prior to Visit 6. If ovulation occurs after Visit 6, scheduling of subsequent visits will be adjusted accordingly.

Table 3: Study Design and Schedule of Assessments

		Screening/ Enrollment	Pre-treatment Cycle[1]		IVR in place					After IVR removal		
Visit #		Visit 1	Visit 2	Visit 3	Visit 4[2] (IVR insertion)		Visit 5 (24 h p Visit 4)	Visit 6[3]	Visit 7 (IVR removal)	Visit 8 (24 h p Visit 7)	Visit 9[4] (72 h p Visit 7)	Follow-up call / contact
					Pre-insertion	Post-insertion						
Informed consent		✓										
Screening	Review inclusion/exclusion criteria	✓	✓	✓								
	Demographic info/medical history/interval history	✓	✓	✓								
	Height, weight, and blood pressure	✓										
	Directed physical exam	(✓)	(✓)									
	Urine – pregnancy test.<br>If indicated: dipstick, microscopy and culture	✓	✓									
	Blood – HIV (with counseling), HBsAg, chemistries, fasting lipids, CBC	✓										
	Blood – P4		✓[1]									
	Pelvic – Bimanual, GC, CT, trich, pH, Nugent Score<br>If indicated: Pap and wet prep	✓	(✓)									
Randomize and dispense assigned IVR; weigh IVR					✓							
Directed Physical Exam				(✓)	(✓)		(✓)	(✓)	(✓)	(✓)	(✓)	
Blood	Safety (chemistries, fasting lipids, CBC)								✓			
	PK: TFV, LNG (including SHBG)				✓	✓<br>(1,2,4, and 8 h)	✓	✓	✓	✓		
	PK: TFV-DP								✓			
	PD LNG (P4)								✓			
	PD LNG (Estradiol)				✓			✓[3]	✓			
Urine	Urine pregnancy test			✓	✓			(✓)	✓			
	If indicated: dipstick, microscopy and culture			(✓)	(✓)		(✓)	(✓)	(✓)	(✓)	(✓)	

			Screening/ Enrollment	Pre-treatment Cycle[1]		IVR in place					After IVR removal		
**Visit #**			**Visit 1**	**Visit 2**	**Visit 3**	**Visit 4[2] (IVR insertion)**		**Visit 5 (24 h p Visit 4)**	**Visit 6[3]**	**Visit 7 (IVR removal)**	**Visit 8 (24 h p Visit 7)**	**Visit 9[4] (72 h p Visit 7)**	**Follow-up call / contact**
						**Pre-insertion**	**Post-insertion**						
Pelvic exam and genital samples	Pelvic exam				✓	✓	✓ (1,2,4,or 8 h)[5]	✓	✓	✓	✓	✓	
	If indicated: wet prep & pH				(✓)	(✓)		(✓)	(✓)	(✓)	(✓)		
	Aspirate (PK TFV)						✓ (1,2,4,or 8 h)[5]	✓	✓	✓	✓		
	Swab (PK TFV)[6]						✓ (1,2,4,or 8 h)[5]	✓	✓	✓	✓		
	Swab (PK LNG)					✓			✓				
	Microflora swabs (safety)					✓				✓			
	Swab (PD TFV)					✓				✓			
	Swab for semen test					✓				✓			
	CVL (safety, PD TFV)					✓				✓			
	Colposcopy					✓		✓	✓	✓			
	Cervical mucus (PK LNG)								✓[3]	✓	✓		
	Cervical mucus (PD LNG)								✓[3]				
	Transvaginal Ultrasound (PD LNG)				✓					✓			
	Endometrial biopsy (Expl PD LNG, safety)[7] (EVMS only)				✓					✓			
	Cerv/vag biopsies:	Expl PD TFV (1, EVMS only)			✓					✓			
		Safety (2 EVMS/1 DR)			✓					✓			
		PK TFV, TFV-DP (both clinical sites)[8]						✓ (2)		✓ (2)	✓ (1)[9]	✓ (1)[9]	
Acceptability questionnaire										✓			
Review guidebook, record AEs, CTs, menses, etc.						✓	✓	✓	✓[3]	✓	✓	✓	
Confirm followed instructions re: intercourse and vaginal activity				✓	✓	✓	✓	✓	✓	✓	✓	✓	

	Screening/ Enrollment	Pre-treatment Cycle[1]		IVR in place					After IVR removal		
**Visit #**	**Visit 1**	**Visit 2**	**Visit 3**	**Visit 4[2] (IVR insertion)**		**Visit 5 (24 h p Visit 4)**	**Visit 6[3]**	**Visit 7 (IVR removal)**	**Visit 8 (24 h p Visit 7)**	**Visit 9[4] (72 h p Visit 7)**	**Follow-up call / contact**
				**Pre-insertion**	**Post-insertion**						
Dispense or collect guidebook			✓						(✓)	✓	
Dispense ovulation predictor kit						✓[10]					
Collect IVR, complete IVR processing (photography, etc.)								✓			
Study exit											✓

1 P4 will be drawn on Day 21 (Visit 2). If the result is ≥ 3 ng/ml, the participant will return for Visit 3. If P4 <3 ng/ml at Visit 2, repeat testing will be done on Day 23 (repeat Visit 2). If the repeat testing yields a serum P4 of ≥ 3 ng/mL, the participant will return for Visit 3. If the P4 at repeat Visit 2 is <3. ng/mL, the participant will be discontinued.

2 IVR insertion will be on Day 7 of the menstrual cycle. Initial IVR insertion at Visit 4 will be witnessed by clinic staff.

3 Some procedures from Visit 6 will be repeated if the participant has a positive ovulation predictor kit test following Visit 6. At repeat Visit 6, AEs, CTs and menses information will be reviewed, blood will be drawn to assess LNG PD (estradiol) and cervical mucus will be collected for LNG PK/PD.

4 Visit 9 will only occur for those randomized to this collection time point for PK biopsy.

5 Participants will be randomized to one of the following time points for pelvic exam and genital specimen collection: 1, 2, 4, or 8 hours after IVR insertion

6 At Visit 5 and Visit 7 (pre-removal), 3 swabs for TFV PK will be collected: near the IVR, about 2/3 of the distance from the posterior fornix to the introitus, and on the ectocervix, in an effort to demonstrate correlation between the less invasive assay (swab) and more invasive assay (biopsy). At Visit 4 (post-insertion), Visit 6 and Visit 8, 1 swab will be collected from near the IVR to assess correlation between swabs and aspirates.

7 EVMS only. Endometrial biopsies will be split with one specimen being assayed for morphometric analysis and endometrial dating and the other being analyzed for markers of progestational effects.

8 One or two biopsies will be taken, as indicated by the number in parentheses. If only one is taken, it will be located near the IVR position. If two are taken, one will be near the IVR position and the other about 2/3 of the distance from the posterior fornix to the introitus.

9 Participants will be randomized to one of the following time points for one proximal biopsy: Visit 8 (24 hours post IVR removal) or Visit 9 (72 hours post IVR removal).

10 Participants will start using the ovulation predictor kit on Day 10 of their cycle and will notify the clinic on the day it becomes positive. They will be seen that day or as soon as possible afterward, and will continue testing until the morning of Visit 6. If it does not turn positive by Day 16, they will come in for Visit 6 no later than Day 17and will continue use of the predictor kit following Visit 6.

Table 4: Relationship of Study Visits to IVR Day and Cycle Day

	Screening/ Enrollment	Pre-treatment Cycle		IVR in place				After IVR removal	
**Visit #**	Visit 1	Visit 2	Visit 3	Visit 4[1]	Visit 5	Visit 6[2]	Visit 7[3]	Visit 8	Visit 9
**IVR Day**		~ -14	~ -10	1	2	~8	~16-18	~17-19	~19-21
**Cycle Day**	Any day	21	24	7	8	~14	~22-24	~23-25	~25-27

[1] IVR insertion
[2] Visit 6 to occur as close as possible to day of ovulation, but no later than Day 17. Some procedures from Visit 6 may be repeated if ovulation occurs after Day 17.
[3] IVR removal. Visit 7 should occur 8-10 days after Visit 6 or repeat Visit 6

7.2. Anticipated Length of Study

Recruitment for the study is expected to take approximately 6 to 8 months. Each participant is expected to complete the study in about 3 months. Therefore, the clinical portion of the study is expected to be completed in about 9 to 11 months. Lab assays are expected to take an additional 3 to 4 months. Data analysis is expected to take approximately 3 months and preparation of the final report 2 additional months.

7.3. Number of Subjects

Approximately 100 women may be consented and undergo assessment procedures in order to have at least 50 women complete the study.

7.4. Treatment Assignment

Five treatment codes (A1-E5) will be randomly assigned to treatment group such that 2 codes are randomly associated with each of the two active groups (TFV/LNG and TFV-only) and 1 code is associated with the placebo ring. Participants will be randomized to one of the 5 codes, stratified by center, in a 1:1:1:1:1 ratio. This will effectively randomize women to treatment group in a 2:2:1 ratio (TFV/LNG:TFV-only:placebo).

7.5. Criteria for Study Termination

Recruitment for the study or a treatment arm may be stopped at one or both sites if, in the opinion of CONRAD:

- Review of AEs shows an unexpected, significant or unacceptable risk to the participants enrolled in the study or treatment arm (AEs will be monitored by the Clinical Study Leader and a decision to discontinue the study because of safety will take into account whether the AEs were possibly product-related, were serious, and/or led to participant discontinuation)
- The site has failed to enroll subjects at an acceptable rate
- The protocol requirements have not been adhered to and/or
- Administrative reasons

8. SELECTION AND WITHDRAWAL OF PARTICIPANTS

The volunteers for this study will be recruited using a variety of methods, including through existing databases (as protected health information permits) and through advertisements in local media outlets which have been approved by the local Institutional Review Board (IRB).

Women will be recruited since vaginal rings can only be used by women and the endpoints require genital sampling. Although the National Institutes of Health (NIH) has mandated that children, defined as younger than 21 years old, be included in research trials when appropriate, this study fits one of the "Justifications for Exclusion" as set forth by the NIH. Specifically, "insufficient data are available in adults to judge potential risk in children" and "children should not be the initial group to be involved in research studies." We will recruit children aged 18 to 20 years who can legally provide informed consent. Efforts will be made by the sites to recruit subjects so that the racial and ethnic characteristics of the subject population will reflect the demographics of the study sites. No selection criteria shall be based on race or ethnicity.

8.1. Inclusion Criteria

Volunteers must meet all of the following criteria prior to genital sampling at Visit 3.

- Age 18-45 years, inclusive
- General good health (by volunteer history and per investigator discretion) without any clinically significant systemic disease (including, but not limited to significant liver disease/hepatitis, gastrointestinal disease, kidney disease, thyroid disease, osteoporosis or bone disease, and diabetes)
- Currently having regular menstrual cycles of 26-35 days by participant report
- History of Pap smears and follow-up consistent with standard medical practice as outlined in the study manual or willing to undergo a Pap smear
- Protected from pregnancy by one of the following:
    - Sterilization of either partner. *Note: Women protected from pregnancy by sterilization of either partner must abstain from vaginal intercourse from 48 hours prior to Visit 3 until the sixth day after the last study visit.*
    - Willing to abstain from vaginal intercourse from Visit 1 until the sixth day after the last study visit.
- Willing to abstain from any other vaginal activity and the use of vaginal product other than the study product including tampons, spermicides, lubricants, and douches starting 48 hours before Visit 3 until the sixth day after the last study visit
- Vaginal and cervical anatomy that, in the opinion of the investigator, lends itself to easy colposcopy and genital tract sample collection
- Negative urine pregnancy test
- P4 $\geq$3 ng/ml

- Willing to give voluntary consent, sign an informed consent form and comply with study procedures as required by the protocol

8.2. Exclusion Criteria

Volunteers must meet none of the following criteria prior to genital sampling at Visit 3.

- BMI ≥ 30 kg/m^2
- History of hysterectomy
- Currently pregnant or within two calendar months from the last pregnancy outcome. *Note: If recently pregnant must have had at least two spontaneous menses since pregnancy outcome.*
- Use of any hormonal contraceptive method in the last 3 months (oral, transdermal, transvaginal, implant, or hormonal intrauterine contraceptive device)
- Injection of Depo-Provera in the last 10 months
- Use of copper IUD after Visit 1
- Currently breastfeeding or having breastfed an infant in the last two months, or planning to breastfeed during the course of the study
- History of sensitivity/allergy to any component of:
    - TFV 1% gel
    - Topical anesthetic, or allergy to both silver nitrate and Monsel's solution
- Contraindication to LNG
- In the last six months, diagnosed with or treated for any STI or pelvic inflammatory disease. *Note: Women with a history of genital herpes or condylomata who have been asymptomatic for at least six months may be considered for eligibility.*
- Nugent score greater than or equal to 7 or symptomatic bacterial vaginosis (BV) as defined by Amsel's criteria
- Positive test for *Trichomonas vaginalis*, *Neisseria gonorrhea* (GC), *Chlamydia trachomatis* (CT), HIV, or Hepatitis B surface antigen (HBsAg)
- Known bleeding disorder that could lead to prolonged or continuous bleeding with biopsy
- Chronic or acute vulvar or vaginal symptoms (pain, irritation, spotting, etc.)
- Known current drug or alcohol abuse which could impact study compliance
- Grade 2 or higher laboratory abnormality, per the August 2009 update of the Division of AIDS, National Institute of Allergy and Infectious Disease (DAIDS) Table for Grading the Severity of Adverse Events, or clinically significant laboratory abnormality as determined by the clinician
- Systemic use in the last two weeks or anticipated use during the study of any of the following: corticosteroids, antibiotics, anticoagulants or other drugs known to prolong bleeding and/or clotting, antifungals, CYP3A4 inducers or inhibitors (e.g., St. John's Wort or erythromycin), antivirals (e.g., acyclovir or valacyclovir) or antiretrovirals (e.g., Viread, Atripla®, Emtriva®,

Complera®). *Note: Participants should avoid non-steroidal anti-inflammatory drugs (NSAIDs) except for treatment of dysmenorrhea during menses. Participants may use Tylenol® on an as-needed but not daily basis during the study.*[1]

- Participation in any other investigational trial (device, drug, or vaginal trial) within the last 30 days or planned participation in any other investigational trial during the study
- History of gynecological procedures (including genital piercing) on the external genitalia, vagina or cervix within the last 14 days
- Abnormal finding on laboratory or physical examination or a social or medical condition which, in the opinion of the investigator, would make participation in the study unsafe or would complicate interpretation of data

8.3. Participant Withdrawal

8.3.1. Withdrawal Criteria

Participants who sign the informed consent and agree to participate in the study but do not meet eligibility criteria will not undergo initial genital sampling procedures (Visit 3) and will not continue in the study. No case report forms (CRFs) will be completed for participants who do not undergo initial genital sampling.

Once a participant undergoes initial genital sampling procedures, she may be withdrawn from the study for the following reasons:

- Failure to follow protocol requirements that is judged severe enough by the investigator to significantly affect study outcomes
- Pregnancy or desire to become pregnant
- Use of exogenous hormones including systemic corticosteroid therapy
- Medical reasons, including diagnosis of an STI or symptomatic BV
- Personal reasons (participant request)
- Discontinuation of treatment arm, her site, or of entire study (Section 7.5)

8.3.2. Replacement

At least fifty women will complete the study. Participants who are randomized but do not complete all genital sampling will contribute to the analysis as described in Section 14 but additional volunteers may be enrolled until the target number of completers is achieved. Participants who initiate ring use but discontinue prior to completion may not re-enroll.

[1] Drugs such as Tylenol that can affect liver function and drugs that affect renal function may affect metabolism and should not be used on a daily basis during the study.

8.3.3. Follow-up

If the participant discontinues from the study after Visit 3 but prior to completing all genital sampling, the site may ask her to return for study procedures, if she is willing and if appropriate. She will be asked about medications taken and AEs including genital symptoms since the last visit. If she undergoes biopsy procedures, she will be contacted by telephone approximately one to two weeks after the biopsy. She will be exited from the study during her last contact with the site.

9. STUDY PROCEDURES

Prospective participants may be pre-screened by telephone: the study will be explained, the inclusion and exclusion criteria reviewed, volunteers' questions answered, and Visit 1 scheduled. Each woman will be seen in approximately 8 or 9 visits (depending on randomization) and have one scheduled follow-up call/contact.

9.1. Visit 1: Screening/Enrollment

Visit 1 should be scheduled at a time when the volunteer does not expect to be menstruating. Volunteers will be consented at Visit 1 and undergo procedures to confirm they are eligible to continue in the study.

- The study and informed consent form will be reviewed and all volunteer questions will be answered. If the volunteer wishes to participate and meets preliminary study criteria, she will be asked to review and sign an informed consent form. The Principal Investigator (PI) or designee will sign the form and provide a copy to the participant. Permission will be requested as part of the informed consent process for storage of the biologic samples obtained during the study for results confirmation and/or possible future testing will be explained, as allowed by the site.
- An interview will be conducted to obtain medical history and demographic information.
- Height, weight, and blood pressure will be measured.
- Directed physical exam will be performed if history is significant for medical condition.
- A urine specimen will be obtained for a urine pregnancy test to confirm that the participant is not pregnant and to perform a dipstick urinalysis if symptomatic for infection (specimen will be clean-catch if the participant is symptomatic, and urine microscopy and culture will be performed as clinically indicated).
- An HIV test will be performed, along with appropriate counseling and/or referral.
- Blood samples will be collected for CBC, serum chemistries, fasting lipids, and HBsAg testing.
- A pelvic examination (including bimanual examination) will be performed with findings managed as outlined in Table 12: *Safety Management Plan for Local Toxicity* and below:
    - Sample for wet mount will be taken during the pelvic exam if the participant is symptomatic for vulvovaginal candidiasis or BV
    - Sample for *Trichomonas vaginalis* will be taken
    - Gram stain and pH will be performed at the site to assess for BV
    - Specimens for GC and CT will be collected
    - A Pap smear will be performed, as consistent with current practice guidelines
- Symptomatic urinary tract infection (UTI) and yeast infections will be treated, preferably with oral medication. If symptomatic trichomoniasis or BV is diagnosed, the participant will be treated and will not be allowed to continue in the study.

- The participant will be instructed to abstain from vaginal intercourse starting after Visit 1 through the sixth day after the last study visit. (Women who are sterilized or who have a sterilized partner must abstain from vaginal intercourse starting 48 hours before Visit 3 through the sixth day after the last study visit.)

There should be sufficient time between Visit 1 and Visit 2 to allow any needed lab results to become available. If any tests reveal that the participant does not meet inclusion/exclusion criteria, the participant will not continue to Visit 2. If more than eight weeks pass between Visit 1 and Visit 2, then Visit 1 procedures must be repeated, as described in the study manual.

9.2. Visit 2: Confirmation of Ovulation

Visit 2 will be scheduled for day 21 (+/- 1 day) of the participant's menstrual cycle to confirm ovulation.

- An interval medical history will be collected.
- Eligibility criteria will be reviewed. If any lab tests reveal that the participant is no longer eligible, the visit should not proceed and the participant will not continue in the study.
- The study staff will ask the participant if she followed the instructions regarding vaginal intercourse. If she did not, she will be reminded of protocol requirements.
- A directed physical exam, dipstick urinalysis (and possibly microscopy/cultures) and/or wet mount/pH will be performed, if indicated. If a symptomatic yeast or UTI infection is diagnosed, the participant will be treated and Visit 2 will be rescheduled for the next menstrual cycle. If symptomatic trichomoniasis or BV is diagnosed, the participant will be treated and will not be allowed to continue in the study.
- A urine specimen will be obtained for a urine pregnancy test.
- Blood will be drawn for P4 level.
- The participant will be instructed to abstain from all other vaginal activity starting 48 hours before Visit 3 until the sixth day after the last study visit. This includes:
  - Anal sex, receiving oral sex, digital vaginal stimulation, and use of sex toys;
  - Use of any vaginal product other than the study product including tampons, spermicides, lubricants, and douches (for intermenstrual spotting or bleeding, unscented menstrual pads but not tampons may be used); and
  - Any other vaginal activity.
- The participant will be reminded to abstain from all vaginal intercourse through the sixth day after the last study visit. (Women who are sterilized or who have a sterilized partner must abstain from vaginal intercourse starting 48 hours before Visit 3 through the sixth day after the last study visit.)

If ovulation is not confirmed at Visit 2 by a P4 of $\geq$3 ng/ml, Visit 2 will be repeated in the same menstrual cycle; repeat Visit 2 will be scheduled on day 23 (+/- 1 day) of the participant's menstrual cycle and a urine pregnancy test will not be required. If ovulation is not confirmed at repeat Visit 2, the participant is not eligible to continue in the study.

If ovulation is confirmed at Visit 2 or repeat Visit 2 by a P4 of ≥3 ng/ml, Visit 3 will be scheduled on or before day 24 (but not later than day 26) of the participant's menstrual cycle. If results are available and ovulation is confirmed, and the participant confirms she has not engaged in any vaginal activity or used any vaginal products in the prior 48 hours, Visit 3 may occur on the same day as Visit 2.

9.3. Visit 3: Baseline Sample Collection

Once it has been confirmed that the participant meets all of the inclusion criteria and none of the exclusion criteria, baseline blood and genital tract samples will be taken at Visit 3. The visit should be scheduled on or before day 24 (but not later than day 26) of the same menstrual cycle in which ovulation was confirmed.

- An interval medical history will be collected.
- The study staff will ask the participant if she followed the instructions regarding vaginal products and vaginal activity. If she did not, she will be reminded of protocol requirements. Visit 2 and 3 may be rescheduled for her next menstrual cycle at the investigator's discretion.
- A directed physical exam, dipstick urinalysis (and possibly microscopy/cultures) and/or wet mount/pH will be performed, if indicated. If a symptomatic yeast or UTI infection is diagnosed, the participant will be treated and Visits 2 and 3 will be rescheduled for the next menstrual cycle. If symptomatic trichomoniasis or BV is diagnosed, the participant will be treated and discontinued.
- A urine specimen will be obtained for a urine pregnancy test.

If the participant meets all eligibility criteria and agrees to participate, she will continue in the study.

- A pelvic exam will be performed and the following procedures will be completed in the order listed. Findings will be managed as outlined in Table 12: *Safety Management Plan for Local Toxicity.*
    - Transvaginal ultrasound to assess endometrial thickness.
    - Collection and processing of one endometrial biopsy (EVMS site only) for LNG PD as outlined in the study manual.
    - Collection and processing of cervicovaginal biopsies for safety (one at Profamilia and two at EVMS) and TFV PD (one biopsy at EVMS) as outlined in the study manual.
    - Applicable post-biopsy instructions will be discussed with the participant.
- A participant guidebook (which will include instructions regarding post-biopsy care, vaginal restrictions, and visit information) will be given to the participant to record any medications she takes or symptoms she experiences for the duration of the study, sexual and vaginal activity, removal/expulsion and reinsertion of study product, as well as menses and intermenstrual spotting. She will be instructed to bring the guidebook to every visit.
- The participant will be instructed to contact the site at any time during the study if she experiences:
    - Fever

- Moderate to severe urogenital symptoms (e.g., genital burning, irritation, stinging, pressure, rash, itching, discharge, odor, urgency, dysuria, or hematuria)
- Pelvic/lower abdominal pain
- Moderate to heavy intermenstrual bleeding (more than she would have during a normal period) or, during the week following a biopsy, any bleeding more than spotting

She will be asked to come in for an evaluation, if indicated.

- The participant will be reminded of the restrictions on vaginal activity (including intercourse) and product use as described at Visit 2.

Visit 4 will take place on day 7 (+/- 1 day) of one of the first two menstrual cycles after Visit 3, ideally in the first menstrual cycle.

9.4. Visit 4: Randomization, Initiation of IVR Use

Visit 4 will be carried out to randomize eligible participants to treatment group and sample collection timepoints, initiate use of the IVR, and carry out endpoint assessments post-insertion of the IVR. The visit will be scheduled on day 7 (+/- 1 day) of the participant's menstrual cycle.

- The guidebook, including reported AEs, concomitant therapies (CTs) and the menses diary, will be reviewed.
- The study staff will ask the participant if she followed the instructions regarding use of vaginal products and vaginal activity. If she did not, she will be reminded of protocol requirements and may be rescheduled for her next menstrual cycle on cycle day 7, at the investigator's discretion.
- A directed physical exam, dipstick urinalysis (and possibly microscopy/cultures) and/or wet mount/pH will be performed, if indicated. If a symptomatic yeast or UTI infection is diagnosed, the participant will be treated and Visit 4 will be rescheduled for the next menstrual cycle, on cycle day 7. If symptomatic trichomoniasis or BV is diagnosed, the participant will be treated and discontinued.
- A urine specimen will be obtained for a urine pregnancy test.
- Blood will be drawn for assessment of baseline TFV PK, LNG/SHBG PK and LNG PD (estradiol) levels.
- A pelvic exam and the following procedures will be completed in the order listed. Findings will be managed as outlined in Table 12: *Safety Management Plan for Local Toxicity*.
    - The biopsy sites from previous sampling will be assessed. Subsequent procedures will be done at the investigator's discretion.
    - Preparation of vaginal swabs to test for microflora and semen biomarkers as outlined in the study manual. If the semen biomarker is positive, the participant will be reminded of protocol requirements and Visit 4 will be rescheduled for the next menstrual cycle, on cycle day 7.
    - Preparation of vaginal swab for baseline LNG PK and TFV PD.
    - Collection of CVL for safety and TFV PD.

    - Naked eye/colposcopic examination. All colposcopic exams in this study will be done according to the WHO/CONRAD Manual for the Standardization of Colposcopy for the Evaluation of Vaginal Products, Update 2004, to the extent possible.

- Study staff will open the next sequentially numbered, sealed randomization envelope to obtain the participant's treatment code A1-E5. The envelope will also provide assignment of genital sample collection time point at Visit 4 (1, 2, 4, or 8 hour post IVR insertion) and TFV PK biopsy collection time point post IVR removal (24 or 72 hours). It is considered a protocol violation to open envelopes out of sequence or prior to randomization.
- The following procedures will be completed in the order listed.
    - The IVR identified with the participant's treatment code will be retrieved. The IVR will be weighed by clinic staff prior to IVR insertion.
    - The participant will insert the IVR into her vagina, under the supervision of clinic staff.
    - Blood will be drawn for TFV PK at 1, 2, 4, and 8 hours after IVR insertion.
    - Other study procedures will be carried out at the participant's assigned sample collection time point (1, 2, 4, or 8 hours) after IVR insertion.
        - A pelvic exam will be performed and the following procedures will be completed in the order listed. Findings will be managed as outlined in Table 12: *Safety Management Plan for Local Toxicity*.
        - Collection of vaginal aspirate for TFV PK.
        - Preparation of vaginal swab for TFV PK.
- Participants will be reminded:
    - Not to remove the IVR but, if removal becomes necessary for some reason, or if the IVR is expelled, to record the time of expulsion/removal and reinsertion in their guidebook. If, for some reason, reinsertion is not possible, the participant should put the IVR into a plastic zip-lock type bag and bring it to the clinic at her next visit.
    - To contact the site as soon as possible during IVR use if she notices an obvious visual defect in the IVR (e.g., a failure of the weld between drug-containing segments, made apparent by the IVR no longer being a complete circle).
    - Of the restrictions on vaginal activity (including intercourse) and product use as described at Visit 2.
    - About use of the guidebook and to bring the guidebook to the next visit.
    - To contact the site about symptoms as described in Visit 3.

If any symptoms or signs attributable to the use of the IVR develop between IVR insertion and the genital sampling time point, AEs should be documented as outlined in Section 13, and the participant can continue at the investigator's discretion.

9.5. Visit 5: 24-hour Sample Collection, IVR Day 2

Visit 5 will take place to allow for sample collection to take place approximately 24 hours after initial IVR insertion.

- The guidebook, including reported AEs, CTs and the menses diary, will be reviewed.
- The study staff will ask the participant if she followed the instructions regarding vaginal intercourse, vaginal products and vaginal activity. If she did not, she will be reminded of protocol requirements and the visit will continue.
- A directed physical exam, dipstick urinalysis (and possibly microscopy/cultures) and/or wet mount/pH will be performed, if indicated. If a symptomatic yeast or UTI infection is diagnosed, the participant will be treated with the shortest treatment consistent with the site's standard of care and the visit will continue. If symptomatic trichomoniasis or BV is diagnosed, the participant will be treated and discontinued.
- A blood sample for TFV and LNG/SHBG PK will be taken.
- A pelvic exam will be performed and the following procedures will be completed in the order listed. Findings will be managed as outlined in Table 12: *Safety Management Plan for Local Toxicity*.
    - The biopsy sites from previous sampling will be assessed. Subsequent procedures will be done at the investigator's discretion.
    - Collection of vaginal aspirate for TFV PK.
    - Preparation of vaginal swabs for TFV PK as outlined in the study manual.
    - Naked eye/colposcopic examination.
    - Collection and processing of up to two biopsies for TFV PK as outlined in the study manual.
    - Post-biopsy instructions will be discussed with the participant.
- An ovulation predictor kit will be given to the participant. Starting on day 10 of the participant's menstrual cycle, she will use the kit to determine the day of ovulation. The participant will be instructed to notify the clinic on the day it becomes positive. The participant will be seen that day or as soon as possible afterward. The participant will be instructed to continue testing following the first positive result, until the morning of Visit 6. If the ovulation predictor kit does not turn positive by day 16 of the participant's menstrual cycle, Visit 6 will occur no later than day 17 of the participant's cycle and the participant will continue use of the predictor kit.
- The participant will be given reminders as described in Visit 4.

9.6. Visit 6: Ovulation Sample Collection

Visit 6 should occur as close as possible to the day of ovulation, based on the ovulation predictor kit, or as soon as possible afterward. If the ovulation predictor kit does not turn positive by Day 16 of the participant's menstrual cycle, Visit 6 will occur no later than day 17 of the participant's cycle.

- The guidebook, including reported AEs, CTs and the menses diary, will be reviewed.

- The study staff will ask the participant if she followed the instructions regarding vaginal products and vaginal activity. If she did not, she will be reminded of protocol requirements and the visit will continue.
- A directed physical exam, pregnancy test, dipstick urinalysis (and possibly microscopy/cultures) and/or wet mount/pH will be performed, if indicated. If a symptomatic yeast or UTI infection is diagnosed, the participant will be treated with the shortest treatment consistent with the site's standard of care and the visit will continue. If pregnancy, symptomatic trichomoniasis or BV is diagnosed, the participant will be treated, as necessary, and discontinued.
- Blood will be drawn for TFV PK and LNG/SHBG PK/PD.
- A pelvic exam will be performed and the following procedures will be completed in the order listed. Findings will be managed as outlined in Table 12: *Safety Management Plan for Local Toxicity*.
    - The biopsy sites from previous sampling will be assessed. Subsequent procedures will be done at the investigator's discretion.
    - Collection of vaginal aspirate for TFV PK.
    - Preparation of vaginal swab for TFV PK.
    - Preparation of vaginal swab for LNG PK.
    - Naked eye/colposcopic examination.
    - Collection of cervical mucus for LNG PK and PD.
- Participants who did not have a positive ovulation predictor kit result prior to Visit 6 will be instructed to continue testing with the kit following Visit 6. The participant will be asked to contact the site as soon as possible if/when the kit turns positive.
    - If the ovulation predictor kit turns positive following Visit 6, some procedures from Visit 6 will be repeated. Repeat Visit 6 should be scheduled as soon as possible following the positive result. The participant will be instructed to continue testing until the morning of repeat Visit 6.
    - At repeat Visit 6, the following procedures will occur:
        - The guidebook, including reported AEs, concomitant therapies (CTs) and the menses diary, will be reviewed.
        - Blood will be drawn for LNG PD (estradiol).
        - Collection of cervical mucus for LNG PK and PD.
- The participant will be given reminders as described in Visit 4.
- Visit 7 will be scheduled to occur 8-10 days after Visit 6 (or repeat Visit 6).

9.7. Visit 7: IVR Removal and Sample Collection

Visit 7 should occur 8-10 days after Visit 6 (or repeat Visit 6) to allow for the maximum number of days of IVR use in this menstrual cycle while ensuring that participants are seen for final genital sampling before their next menses begins.

- The guidebook, including reported AEs, CTs and the menses diary, will be reviewed.
- The study staff will ask the participant if she followed the instructions regarding vaginal products and vaginal activity. If she did not, she will be reminded of protocol requirements and the visit will continue.
- The participant will complete an acceptability questionnaire.
- A urine specimen will be obtained for a urine pregnancy test.
- A directed physical exam, dipstick urinalysis (and possibly microscopy/cultures) and/or wet mount/pH will be performed, if indicated. If a symptomatic infection is diagnosed, study procedures will continue per investigator discretion and the participant will be treated after completion of the visit procedures.
- Blood will be drawn for TFV and TFV-DP PK, LNG/SHBG PK/PD, CBC, serum chemistry, fasting lipids, and P4.
- A pelvic exam will be performed and the following procedures will be completed in the order listed. Findings will be managed as outlined in Table 12: *Safety Management Plan for Local Toxicity*.
    - The biopsy sites from previous sampling will be assessed. Subsequent procedures will be done at the investigator's discretion.
    - Collection of vaginal aspirate for TFV PK.
    - Preparation of vaginal swabs for TFV PK, TFV PD, to test for microflora, and semen biomarkers as outlined in the study manual.
    - Collection of CVL for safety and TFV PD.
    - Naked eye/colposcopic examination.
    - Collection of cervical mucus for LNG PK.
    - Transvaginal ultrasound to assess endometrial thickness.
    - The IVR will be removed.
    - Collection and processing of one endometrial biopsy (EVMS only) for LNG PD as outlined in the study manual.
    - Collection and processing of up to three biopsies (up to five at EVMS) for safety and TFV and TFV-DP PK, as outlined in the study manual.
    - Appropriate post-biopsy instructions will be discussed with the participant.
- Post-removal ring procedures will be immediately carried out as outlined in the study manual.
- The participant will be given applicable reminders as described in Visit 4.

- Visit 8 will be scheduled to allow for sample collection 24 hours after IVR removal.

9.8. Visit 8: 24 hour Post-IVR Sample Collection

Visit 8 will be scheduled to allow for sample collection to take place 24 hours after IVR removal.

- The guidebook, including reported AEs, CTs and menses diary, will be reviewed. For participants randomized to have final genital sampling at Visit 8, the guidebook will be collected and the participant will be given new guidebook pages to record any CTs or AEs and a copy of the post-biopsy instructions. *(Note: The guidebook pages dispensed at Visit 8 will be reviewed during the follow-up telephone call/visit but will not be collected.)*
- The study staff will ask the participant if she followed the instructions regarding vaginal products and vaginal activity. If she did not, she will be reminded of protocol requirements and the visit will continue.
- A directed physical exam, dipstick urinalysis (and possibly microscopy/cultures) and/or wet mount/pH will be performed, if indicated. If a symptomatic infection is diagnosed, study procedures will continue per investigator discretion and the participant will be treated after completion of the visit procedures.
- Blood will be drawn for TFV and LNG/SHBG PK.
- A pelvic exam will be performed and the following procedures will be completed in the order listed. Findings will be managed as outlined in Table 12: *Safety Management Plan for Local Toxicity*.
    - The biopsy sites from previous sampling will be assessed. Subsequent procedures will be done at the investigator's discretion.
    - Collection of vaginal aspirate for TFV PK.
    - Preparation of a vaginal swab for TFV PK.
    - Collection of cervical mucus for LNG PK.
    - For participants randomized to the 24 hour post IVR removal biopsy, collection and processing of one biopsy for TFV PK, as outlined in the study manual.
- The participant will be reminded of the restrictions on vaginal activity (including intercourse) and product use as described at Visit 2.
- The participant will be reminded about contacting the site about symptoms as in Visit 3.
- If randomized to the 72-hour post-IVR sample collection time point, Visit 9 will be scheduled to allow for sample collection 72 hours after IVR removal.
- If Visit 8 is the participant's last study visit, a follow-up contact will be scheduled for about one to two weeks after Visit 8.

9.9. Visit 9: 72-hour Post-IVR Sample Collection

Visit 9 will be scheduled to allow for sample collection to take place 72 hours after IVR removal. Visit 9 will only occur for those randomized to this collection time point.

- The guidebook, including reported AEs, CTs and menses diary, will be reviewed and collected. The participant will be given new guidebook pages to record any CTTs or AEs and a copy of the post-biopsy instructions. *Note: The guidebook pages dispensed at Visit 9 will be reviewed during the follow-up telephone call/visit but will not be collected).*
- The study staff will ask the participant if she followed the instructions regarding vaginal products and vaginal activity. If she did not, she will be reminded of protocol requirements and the visit will continue.
- A directed physical exam, dipstick urinalysis (and possibly microscopy/cultures) and/or wet mount/pH will be performed, if indicated. If a symptomatic infection is diagnosed, study procedures will continue per investigator discretion and the participant will be treated after completion of the visit procedures.
- A pelvic exam will be performed. Findings will be managed as outlined in Table 12: *Safety Management Plan for Local Toxicity*.
    - The biopsy sites from previous sampling will be assessed. Subsequent procedures will be done at the investigator's discretion.
    - Collection and processing of one biopsy for TFV and TFV-DP PK, as outlined in the study manual.
- The participant will be reminded of the restrictions on vaginal activity (including intercourse) and product use as described at Visit 2.
- The participant will be reminded about contacting the site about symptoms as in Visit 3.
- A follow-up contact will be scheduled for about one to two weeks after Visit 9.

9.10. Follow-up Call/Contact

The site will contact the participant about one to two weeks after the completion of study visits. She will be asked about AEs experienced and medications taken since the last visit. The participant will then be exited from the study, unless she has symptoms that require follow-up.

9.11. Unscheduled Visits

Unscheduled visits may be performed at the participant's request or as deemed necessary by the investigator at any time during the study. The participant will be instructed to contact the site at any time during the study if she experiences fever, moderate to severe urogenital symptoms (e.g., genital burning, irritation, stinging, pressure, rash, itching, discharge, odor, urgency, dysuria, or hematuria), or if she experiences pelvic/lower abdominal pain, moderate to heavy menstrual bleeding (more than she would have during a normal period), or any bleeding (more than spotting) during the week following a biopsy. The participant will be asked to come in for an evaluation, as indicated. She should also contact the site if she loses her IVR or otherwise needs additional supplies.

Unscheduled visits that require examination or interview due to symptoms will be recorded on CRFs.

When an unscheduled visit occurs in response to an AE experienced by a study participant, study staff will assess the reported event clinically and provide or refer the participant to appropriate medical care, as

necessary. All AEs will be evaluated and follow-up of any observed abnormalities will proceed according to Section 13, *Assessment of Safety*.

10. TREATMENT OF PARTICIPANTS

10.1. Study Drug

Table 5: Investigational Product

	Investigational Product		Reference Product
**Product Name:**	Tenofovir	Tenofovir and Levonorgestrel	Placebo
**Unit Dose**	8-10 mg/day TFV	8-10 mg/day TFV and<br>20 µg/day LNG	NA
**Dosage Form:**	Intravaginal ring		
**Route of Administration**	Vaginal		
**Physical Description**	Ring 55.0 mm in diameter, consisting of single segment of polyurethane tubing with an outer diameter of 5.5 mm and filled with white TFV-containing paste	Ring 55.0 mm in diameter, consisting of two segments of polyurethane tubing with an outer diameter of 5.5 mm: a longer segment containing white TFV paste and a shorter one (20 mm) with a white LNG core	Ring with an appearance similar to that of the TFV/LNG IVR
**Manufacturer**	DPT Laboratories (San Antonio, TX USA)		

10.2. Concomitant Medications

Systemic use during the study of any of the following is not permitted: corticosteroids, antibiotics, antifungals, anticoagulants or other drugs known to prolong bleeding and/or clotting, antivirals (e.g., acyclovir or valacylovir) or antiretrovirals (e.g., Viread). *NOTE: Participants should avoid NSAIDs except for treatment of dysmenorrhea during menses. Participants may use Tylenol on an as-needed but not daily basis during the study.*[2]

[2] Drugs such as Tylenol that can affect liver function and drugs that affect renal function may affect metabolism and should not be used on a daily basis during the study.

10.3. Treatment Compliance

The IVR will be inserted by the participant in the clinic at Visit 4 and removed in the clinic at Visit 7. The date and time of any interim removals/expulsions and reinsertions will be recorded on a diary card in the participant guidebook. Treatment compliance will be evaluated by a combination of the following: self-report in guidebook diaries, visible changes documented on photographs on the returned IVRs and IVR weight post-removal at the site.

10.4. Randomization and Blinding

Participants will be randomized to treatment group in a 2:2:1 ratio (TFV/LNG; TFV-only; placebo). At the same time participants will also be randomized to sampling time point for Visit 4 aspirate and PK swabs (1, 2, 4, or 8 hours) and to final biopsy collection (24 hours [Visit 8] or 72 hours [Visit 9] after ring removal) in approximately equal ratios within treatment group. All randomizations will stratify on center. Randomizations will be conducted at FHI 360 by a qualified statistician not otherwise involved in the study, using a validated randomization program. The permuted blocks method with randomly selected block size will be used to generate sequences.

Among 5 possible treatment codes A1-E5, each of the active treatment groups (TFV/LNG and TFV-alone) will be randomly assigned to two blinded treatment codes (e.g., ‘A1’, ‘D4’) and the placebo group will be randomly assigned to one code using a validated closed-deck randomization procedure. The packager will indicate these treatment codes on the appropriate foil pouches in accordance with random assignments provided by FHI 360.

Participants will be randomized to treatment code (A1-E5) in a 1:1:1:1:1 ratio. Since it is not possible to predict the exact number of participants that will be randomized at each site in order to achieve 50 completers overall, more than 50 participants will need to be randomized in order to achieve this goal. Since the exact number of randomizations cannot be pre-defined, some deviation from the planned allocation 2:2:1, as well as some imbalance in sampling time points among completers, might occur.

Assignments will be communicated to sites via special opaque, sealed, tamper-evident envelopes, with center name and Randomization Number on the outside and treatment code and sampling time points on the inside. After a participant is cleared for randomization, the coordinator will open the next envelope in numeric sequence and provide the participant with a foil pouch identified by the assigned treatment code. Linkage between participant ID number, randomization number, and pouch given to the participant will be documented on CRFs. Opening an envelope out of sequence or prior to confirmation of the participant’s eligibility for randomization is a protocol violation.

Paper and electronic copies of randomization sequences will be stored in a secure location not accessible by unauthorized staff (including study staff) until scheduled unblinding which will take place following data base lock.

Unblinding envelopes will be prepared and sent to sites in the event of a medical emergency for which proper care requires knowledge of the participant’s treatment assignment. Special sealed, opaque, tamper-evident envelopes, containing center name and Randomization Number on the outside and true treatment assignment on the inside, will be prepared by the randomization statistician. These envelopes must remain sealed until there is need for emergency unblinding. Site monitors will routinely inspect these envelopes to ensure that they remain sealed.

The TFV-only IVR is visually different from the other two IVRs which are similar and difficult to distinguish from each other. Site staff may be able to determine which codes are assigned to the TFV-only IVR. The participants will not be told which IVR they have received and the lab staff, analysts, and sponsor will be blinded.

As described in Section 14.5, an unblinded interim analysis of drug-concentration data from at least 15 completed participants will be made available to the Director of CONRAD's Product Development Department or other authorized individual, to help inform product development decisions. Confidentiality and blinding of these data will be maintained at FHI 360 and CONRAD by procedures described in Section 14.5.

11. STUDY DRUG MATERIALS AND MANAGEMENT

11.1. Description of Study Drug

The study drugs are supplied by CONRAD as IVRs packaged in individual foil pouches. Each ring has a long white to off-white segment and a short transparent/translucent segment. The ring releases active pharmaceutical ingredients (APIs) of approximately 8-10 mg TFV per day alone or in combination with 20 µg LNG per day, or neither. The rings are formulated with the following excipients: polyurethane, glycerin, water, and modified starch (placebo only).

The API TFV is supplied by Gilead Sciences, Inc. The API LNG is supplied by Industriale Chimica s.r.l. The formulation is manufactured for CONRAD under Good Manufacturing Practice (GMP) at DPT Laboratories (San Antonio, TX USA).

11.2. Study Drug Packaging and Labeling

The site will manage the distribution of the study product according to the protocol. Each site will be provided sufficient study product for its designated number of participants. Supplies will be labeled, packaged, and distributed by The Coghlan Group (Bastrop, TX, USA).

The IVR will be packaged in an individual sealed foil pouch. Each pouch will include the following label:

Figure 1: Study Drug Label

CONRAD A13-128

Sponsor: CONRAD, Arlington, VA, USA, 703-524-4744

Manufactured by: DPT Laboratories, San Antonio, TX, USA
Packaged by: The Coghlan Group, Bastrop, TX, USA

Contents: 1 intravaginal ring containing tenofovir, or the combination of tenofovir and levonorgestrel, or neither drug, and inactive ingredients.

Treatment code: (pre-printed) PTID: __________

Date dispensed: ________________ (dd / mon / yr)

Date returned: ________________ (dd / mon / yr)

For vaginal use only. Keep out of reach of children.
Store at room temperature.
Caution: New Drug – Limited by Federal (United States) law to investigational use

11.3. Study Drug Storage

Study product should be stored in a secure area at room temperature (15° - 30°C) (59° - 86°F).

11.4. Study Drug Preparation

The study drug is ready to use and requires no preparation.

11.5. Administration

Each participant will be randomized to IVR type. The assigned IVR will be provided to the participant at Visit 4. The participant will insert the IVR in the clinic by pushing it in with a finger as deep as it will comfortably go. At Visit 7, the IVR will be removed by the study clinician in the clinic and it will be tested as detailed in the study manual.

11.6. Study Drug Accountability

Ring dispensation will be recorded in the Drug Accountability Log at the site. When the ring is removed at Visit 7, the return of the ring will be recorded in the same log.

11.7. Study Drug Handling and Disposal

All study product remaining at the end of the study will be recorded by the site and reconciled by the site monitor. After reconciliation, the Sponsor will provide instruction regarding disposal or return of product.

12. ASSESSMENT OF ENDPOINTS

Table 6: Types of Specimens to be Collected, Purpose and Sequence of Collection

Type of specimen		Objective							
		**Primary: Safety**	**Secondary: PK**			**Tertiary**	**Exploratory**		
			**TFV**	**TFV-DP**	**LNG**	**PD of LNG – systemic and in cervical mucus**	**PD of TFV**	**PD of LNG in endometrium**	**Exploratory markers of genital safety**
Blood		• Chemistries<br>• Lipids<br>• CBC	✓ in plasma	✓ in PBMCs (at Visit 7 pre-removal only)	• ✓<br>• SHBG	• Progesterone<br>• Estradiol			
CVF	aspirate		✓						
	swab		✓		✓				
Vaginal swab		• Semi-quantitative culture<br>• Quantitative PCR<br>• pH<br>• Nugent score<br>• Semen biomarker (to assist in interpreting immune markers)					• Anti-HSV		
CVL		• Soluble markers of innate mucosal immunity and inflammatory response					• Anti-HIV		
Cervical mucus					✓	• Quality<br>• Sperm migration (simplified slide test per Lewis et al)			
Transvaginal Ultrasound								• Endometrial thickness	
Endometrial biopsy (EVMS only)								(EVMS only. ½ in formalin and ½ in RNA Later)<br>• Histology (e.g., proliferative, secretory, atrophic)<br>• Markers of endometrial function	
Cervicovaginal biopsy		• Immune cell phenotype and activation status	One biopsy can be analyzed for both TFV and TFV-DP PK.<br><br>Depending on the visit, one or two biopsies will be taken, as described in study manual.				EVMS only<br><br>• HIV infectivity in explants		EVMS only<br><br>• Histology / epithelial integrity<br>• Markers of mucosal alteration

12.1. Assessment of Pharmacokinetic and Pharmacodynamics

12.1.1. Pharmacokinetics: Blood Sample Collection

Blood samples will be drawn to assess PK of TFV and LNG as detailed below.

Table 7: Pharmacokinetics: Blood Samples, Concentrations

	Visit 3	Visit 4		Visit 5	Visit 6	Visit 7	Visit 8
		Pre-insertion Baseline	Post-insertion (1,2,4 & 8h)				
Plasma: TFV		✓	✓	✓	✓	✓	✓
Plasma: LNG		✓	✓	✓	✓	✓	✓
PBMCs: TFV-DP						✓	
SHBG: LNG		✓	✓	✓	✓	✓	✓

12.1.2. Pharmacokinetics: Genital Sample Collection

Genital samples (fluid and tissue) will be taken to assess PK as detailed below.

Table 8: Pharmacokinetics: Genital Sample, Concentrations

	Visit 3	Visit 4		Visit 5	Visit 6	Visit 7	Visit 8	Visit 9
		Pre-insertion Baseline	Post-insertion (at assigned time)					
CVF (Aspirate): TFV			✓	✓	✓	✓	✓	
CVF (Swab): TFV			✓	✓	✓	✓	✓	
CVF (Swab): LNG		✓			✓			
Cervical Mucus: LNG					✓[1]	✓	✓	
Genital Tissue: TFV and TFV-DP				✓		✓	✓[2]	✓[2]

[1] Cervical Mucus assessed at Visit 6 and, if needed, repeat Visit 6.

[2] Participants will be randomized to genital tissue collection at either Visit 8 or Visit 9.

12.1.3. Pharmacodynamics: Blood Sample Collection

Blood samples will be drawn to assess PD as detailed below.

Table 9: Pharmacodynamics: Blood Sample Collection

	Visit 2	Visit 3	Visit 4		Visit 5	Visit 6	Visit 7	Visit 8
			Pre-insertion Baseline	Post-insertion (at assigned time)				
P4	✓						✓	
Estradiol Concentration			✓			✓[1]	✓	

[1] Estradiol concentration assessed at Visit 6 and, if needed, repeat Visit 6.

12.1.4. Pharmacodynamics: Genital Sample Collection

Genital samples (fluid and tissue) will be taken to assess PD as detailed below.

Table 10: Pharmacodynamics: Genital Samples

	Visit 3	Visit 4		Visit 5	Visit 6	Visit 7	Visit 8
		Pre-insertion Baseline	Post-insertion (at assigned time)				
CVL, Anti-HIV activity: TFV		✓				✓	
Swab, Anti-HSV activity: TFV		✓				✓	
Cervical mucus: quality: LNG					✓[1]		
Cervical mucus: Sperm Migration Test: LNG					✓[1]		✓
Genital Tissue, Anti-HIV activity[2]: TFV	✓					✓	
Genital Tissue (Endometrium): LNG[2]	✓					✓	
Transvaginal ultrasound, Endometrial thickness: LNG	✓					✓	

[1] Cervical Mucus assessed at Visit 6 and, if needed, repeat Visit 6.

[2] This objective will be at EVMS only.

12.2. Assessment of Safety

12.2.1. Medical History

As part of AE data collection, AEs will be assessed at each study visit; participant report of symptoms will be documented and report of symptoms may result in follow-up laboratory assessments.

12.2.2. Physical and Pelvic Examination

A directed physical exam will be performed at each study visit if indicated, with changes on physical exam documented.

Pelvic and colposcopic exams will be performed to assess safety. Findings will be managed as outlined in Table 12.

Table 11: Safety: Physical and Pelvic Examination

	Visit 3	Visit 4		Visit 5	Visit 6	Visit 7	Visit 8	Visit 9
		Pre-insertion Baseline	Post-insertion (at assigned time)					
Directed Physical Exam	(✓)	(✓)		(✓)	(✓)	(✓)	(✓)	(✓)
Pelvic Exam	✓	✓	✓	✓	✓	✓	✓	✓
Colposcopy		✓		✓	✓	✓		

Table 12: Safety Management Plan for Local Toxicity Observed on Pelvic Exam, including Colposcopy

Finding	Management
Vaginal bleeding	The source of the bleeding should be evaluated. Bleeding from the cervical os should be evaluated per standard of care. If the bleeding is from a previous biopsy site or a deep epithelial disruption, hemostasis should be attempted via direct pressure. Silver nitrate or Monsel's solution may also be applied. If adequate hemostasis is not obtained with these measures, electrocautery or suturing may be used. Samples may be collected per investigator discretion.<br>Product use may continue at investigator discretion.
Epithelial findings:<br>• Erythema, edema, petechiae, ecchymoses (epithelium intact by definition) – includes cervicitis<br>• Peeling (superficial epithelial disruption, by definition)<br>• Ulcers, abrasions, lacerations with superficial or deep epithelial disruption	**If an infection is suspected (e.g., due to erythema, vesicles, concomitant discharge, etc.):**<br>A wet prep with KOH and pH, NAAT for GC/CT, and other tests may be done as indicated.<br>• If candidiasis is diagnosed at **Visit 3**, the participant may be treated and Visit 2 and 3 rescheduled.<br>• If candidiasis is diagnosed at **Visit 4**, the participant may be treated and Visit 4 rescheduled on cycle day 7 of the next cycle.<br>• If candidiasis is diagnosed at **Visit 5-8**, the participant may be treated and the visit will continue.<br>• If trichomoniasis, BV, or herpes simplex is diagnosed at **Visit 3-6**, the participant should be treated and discontinued.<br>• If trichomoniasis, BV, or herpes simplex is diagnosed at **Visit 7-8**, the participant should be treated and the visit will continue.<br>**If the finding is bleeding** (which makes it deep epithelial disruption by definition), it should be managed as described under "Vaginal bleeding," above.<br><br>**For all findings**: If the area is small (less than approximately 3mm at the widest point), samples may be collected or the visit may be rescheduled, per investigator discretion. If the finding is >3 mm, consideration should be given to stopping product use and seeing the participant in unscheduled visits until the finding is resolved or resolving.
Vaginal discharge	A wet prep with KOH and pH <u>should</u> be done, and NAAT for GC/CT should be strong considered. If an infection is diagnosed, it should be managed as described under "Epithelial findings" above.

12.2.3. Genital Assessments

Genital samples (fluid and tissue) will be taken to assess safety as detailed below. Sample analysis will be performed at a central lab.

Table 13: Safety: Genital Assessments

	Visit 3	Visit 4		Visit 5	Visit 6	Visit 7	Visit 8
		Pre-insertion Baseline	Post-insertion (at assigned time)				
CVL: soluble markers of mucosal immunity		✓				✓	
Genital Tissue: histology /immune cell activation	✓					✓	
Genital Tissue: markers of mucosal alteration/ epithelial integrity (EVMS only)	✓					✓	
Swabs: Microflora [1]		✓				✓	
Microbial growth on swabs obtained from returned IVRs and microbial levels in returned IVRs						✓	

[1] Includes: Semi-quantitative culture; Quantitative PCR (Copan Swab); Nugent Score (Gram Stain)

12.2.3.1. Hematology and Blood Chemistry

CBC, lipids (fasting) and serum chemistry will be measured at Visit 1 (the baseline analysis for the study) and at Visit 7 prior to IVR removal. Changes will be assessed for safety.

12.2.3.2. Pregnancy Screen

Pregnancy tests are required at Visits 1, 2, 3, 4 and 7 on all participants. Note, however, participants must be protected from pregnancy as described in Section 8.1 in order to be eligible for study participation.

13. ADVERSE AND SERIOUS ADVERSE EVENTS

13.1. Definition of Adverse Events (AE)

An AE is any untoward medical occurrence associated with the use of an investigational product in humans, whether or not considered product related. An AE can therefore be any unfavorable and unintended sign (including an abnormal laboratory finding), symptom, or disease temporally associated with the use of an investigational product, whether or not considered related to the investigational product. Pre-existing events that increase in frequency or severity in nature during or as a consequence of use of an investigational product in human clinical trials will also be considered as AEs. Any medical condition or clinically significant laboratory abnormality with an onset date before the first date of study product administration is considered to be pre-existing, and should be documented in the source document for the participant.

All AEs starting with Visit 3 before treatment, during treatment, or by the time of the follow-up call one to two weeks after the last study visit, whether or not they are related to the study, must be recorded on forms provided by CONRAD.

According to 21 CFR 312.32 “IND Safety Reports” the following definitions of terms apply to AEs occurring in clinical studies involving drugs.

13.1.1. Suspected Adverse Reaction

A suspected adverse reaction is “any AE for which there is a reasonable possibility that the study product caused the event.” An AE is considered to be a suspected adverse reaction only if there is evidence to suggest a causal relationship between the study product and the AE. Examples include:

- A single occurrence of an event that is uncommon and known to be strongly associated with product exposure (Examples, not necessarily applicable to this study include angioedema, hepatic injury, Stevens-Johnson Syndrome)
- One or more occurrences of an event that is not commonly associated with product exposure, but is otherwise uncommon in the population exposed to the study product (e.g., tendon rupture)
- An aggregate analysis of specific events observed in a clinical trial (such as known consequences of the underlying disease or condition under investigation or other events that commonly occur in the study population independent of product exposure)

13.1.2. Adverse Reaction

An adverse reaction is any AE caused by the study product. Adverse reactions are a *subset* of suspected adverse reactions.

13.1.3. Serious Adverse Event (SAE) or Serious Suspected Adverse Reaction

An AE or suspected adverse reaction is considered “serious” if, in the view of either the investigator or the sponsor, it:

- Results in death;

- Is immediately life-threatening;
- Requires in-patient hospitalization or prolongation of existing hospitalization;
- Results in persistent or significant disability or incapacity;
- Results in a congenital abnormality or birth defect; or
- Is an important medical event that may jeopardize the patient or may require medical or surgical intervention to prevent one of the outcomes listed above.

All SAEs starting with Visit 3, before treatment, during treatment, or by the time of the follow-up call/contact one to two weeks after the last study visit, whether or not they are related to the study, must be recorded on forms provided by CONRAD.

13.1.4. Unexpected Adverse Event or Unexpected Suspected Adverse Reaction

An AE or suspected adverse reaction is considered "unexpected" if it is not listed in the investigator brochure or is not listed at the specificity or severity that has been observed; or, if an investigator brochure is not required or available, is not consistent with the risk information described in the general investigational plan or elsewhere in the current application, as amended. For example (not necessarily applicable to this study), under this definition, hepatic necrosis would be unexpected (by virtue of greater severity) if the investigator brochure referred only to elevated hepatic enzymes or hepatitis. Similarly, cerebral thromboembolism and cerebral vasculitis would be unexpected (by virtue of greater specificity) if the investigator brochure listed only cerebral vascular accidents. "Unexpected," as used in this definition, also refers to AEs or suspected adverse reactions that are mentioned in the investigator brochure as occurring with a class of drugs or as anticipated from the pharmacological properties of the drug, but are not specifically mentioned as occurring with the particular drug under investigation.

13.2. Relationship to Study Drug

An Investigator who is qualified in medicine must make the determination of relationship to the investigational product for each AE. The Investigator should decide whether, in his or her medical judgment, there is a reasonable possibility that the event may have been caused by the investigational product. For each AE, the assessment of relatedness should be made using the following scale:

- Unrelated: Onset of the AE had no reasonable temporal relationship to administration of the study product <u>or</u> a causal relationship to administration of the study product is biologically implausible <u>or</u> the event is attributed to an alternative etiology.
- Possibly Related: Onset of the AE has a reasonable temporal relationship to study product administration <u>and</u> a causal relationship is not biologically implausible.
- Probably Related: Onset of the AE has a strong temporal relationship to administration of the study product that cannot be explained by the participant's clinical state <u>and</u> a causal relationship is not biologically implausible.
- Definitely Related: Onset of the AE shows a distinct temporal relationship to administration of the study product that cannot be explained by the participant's clinical state or other factors <u>or</u> the AE occurs on rechallenge <u>or</u> the AE is a known reaction to the product or chemical group or can be predicted by the product's pharmacology.

If the relationship between the AE/SAE and the investigational product is determined to be “possible,” “probable,” or “definite” the event will be considered to be related to the investigational product for the purposes of expedited regulatory reporting.

13.3. Recording and Grading Adverse Events for Severity

Adverse events spontaneously reported by the participant and/or in response to an open question from the study personnel or revealed by observation will be recorded during the study at the investigational site. The AE term should be reported in standard medical terminology when possible. For each AE, the investigator will evaluate and report the date of onset, date of resolution, intensity, causality, action taken, serious outcome (if applicable), and whether or not it caused the patient to discontinue the study. Each AE will be graded for severity using the August 2009 update of the DAIDS Table for Grading the Severity of Adverse Events which can be found at http://rsc.tech-res.com/safetyandpharmacovigilance/.

Figure 2: Grading the Severity of Adverse Events

Adult and Pediatric AEs	http://rsc.tech-res.com/Document/safetyandpharmacovigilance/ Table_for_Grading_Severity_of_Adult_Pediatric_Adverse_Events.pdf
Female Genital AEs	http://rsc.tech-res.com/Document/safetyandpharmacovigilance/ Addendum_1_Female_Genital_Grading_Table_v1_Nov_2007.pdf

For clinical AEs NOT identified in the DAIDS AE Grading Tables, the following scale (as listed in the DAIDS Table for Grading the Severity of Adult and Pediatric AEs) should be used to grade severity:

- Mild: Symptoms causing no or minimal interference with usual social and functional activities
- Moderate: Symptoms causing greater than minimal interference with usual social and functional activities
- Severe: Symptoms causing inability to perform usual social and functional activities
- Potentially Life-Threatening: Symptoms causing inability to perform basic self-care functions OR medical or operative intervention indicated to prevent permanent impairment, persistent disability, or death

It is important to distinguish between serious and severe AEs. Severity is a measure of intensity whereas seriousness is defined by the criteria under Section 13.1.3. An AE of severe intensity may not necessarily be considered serious.

Colposcopic findings should be reported on colposcopic finding CRFs and should not be reported as AEs on AE CRFs. Colposcopic findings are defined as severe when they involve deep disruption of the epithelium. An area of the epithelium with bleeding should be considered deeply disrupted. Severe colposcopic findings possibly, probably, or definitely related to product use must be immediately reported to CONRAD within 24 hours of discovery. In addition, a completed colposcopic finding CRF must be faxed or emailed to CONRAD as soon as possible.

Should a pregnancy occur, it must be reported as soon as possible to CONRAD and recorded on CONRAD’s pregnancy CRF. The participant will be discontinued from the study and appropriate exit procedures will be followed (Section 8.3.3). Note that pregnancy in itself is not regarded as an AE unless there is a suspicion that an investigational product may have interfered with the effectiveness of a contraceptive medication. If the participant has been exposed to study product, the course of the

pregnancy should be followed until it has an outcome (spontaneous miscarriage, elective termination, normal birth or congenital abnormality). If a pregnancy is diagnosed, study product to which the participant has been assigned may be unblinded. If the participant seeks care outside the site, every effort should be made to obtain her consent for the site to receive a copy of her medical records related to the pregnancy, its outcome and the health of the neonate, if applicable.

Reports of congenital abnormalities/birth defects are SAEs. Spontaneous miscarriages should be reported and handled as AEs. Elective abortions without complications should not be reported as AEs.

13.4. Reporting Serious Adverse Events

All SAEs whether or not considered a suspected drug reaction will be recorded starting with Visit 3, before treatment, during treatment, or by the time of the follow-up call/contact one to two weeks after the last study visit.

Any SAE including those listed in the protocol or investigator brochure must be reported to CONRAD within 24 hours of discovery. If there is any question whether the event meets the criteria for “serious” it should be reported anyway. In addition, a completed SAE Form must be faxed or emailed to CONRAD as soon as possible. The Investigator must complete, sign and date the SAE pages, verifying the accuracy of the information recorded on the SAE pages with the corresponding source documents.

Additional follow-up information, if required or available, should all be faxed to CONRAD within one business day of receipt and this should be completed on a follow-up SAE form and placed with the original SAE information and kept with the appropriate section of the CRF and/or study file.

CONRAD will notify the U.S. Food and Drug Administration (FDA) and all participating investigators (i.e., all investigators to whom CONRAD is providing the relevant drug under its IND(s) or under any investigator's IND) in an IND safety report of potentially serious risks, from clinical trials or any other source, as soon as possible, but in no case later than 15 calendar days after CONRAD determines that the information qualifies for reporting under CFR 312.32(c)(1) which includes serious and unexpected suspected adverse reactions, findings from other studies, findings from animal or in-vitro testing, and increased rates of occurrences of serious suspected adverse reactions. In each IND safety report, CONRAD will identify all IND safety reports previously submitted to FDA concerning a similar suspected adverse reaction, and will analyze the significance of the suspected adverse reaction in light of previous, similar reports or any other relevant information.

CONRAD will notify the FDA of any unexpected fatal or life-threatening suspected adverse reactions as soon as possible, but in no case later than 7 calendar days after CONRAD initially receives the information.

CONRAD will promptly investigate and follow up on all safety information it receives, with the cooperation of the investigator. Follow-up information relevant to an IND safety report will be submitted as soon as the information is available and will be identified as such, i.e., “Follow-up IND Safety Report.”

The investigator is responsible for complying with IRB requirements for AE reporting and supplying CONRAD with copies of such correspondence

The blind may be broken for serious or unexpected AEs if knowledge of the treatment received is necessary for interpreting the event, may be essential for the medical management of the participant, or may provide critical safety information about a drug that could have implications for the ongoing conduct

of the trial (e.g., monitoring, informed consent). In general, if the blind is broken and the participant was receiving placebo, the event should not be reported in an IND safety report because there is not a reasonable possibility that the drug caused the AE. If the blind is broken and the subject was receiving the study drug, the suspected adverse reaction must be reported in an IND safety report.

13.5. Temporary Product Hold/Permanent Discontinuation in Response to Adverse Events

All AEs are defined by the Division of AIDS Table for Grading the Severity of Adult and Pediatric Adverse Events, Version 1.0, December 2004 (Clarification dated August 2009), and Addendum 1, Female Genital Table for Use in Microbicide Studies and Addendum 3, Rectal Grading Table for Use in Microbicide Studies (Clarification dated May 2012).

13.5.1. Grade 1 or 2

In general, a participant who develops a Grade 1 or 2 AE regardless of relationship to study product that is not specifically addressed below may continue product use. If the PI opts to temporarily hold study product, CONRAD must be notified.

Follow-up testing for Grade 2 laboratory test results should be performed at scheduled study visits, at a minimum, until resolution or stabilization has been documented. More frequent testing may be performed at any time if required to properly monitor and/or manage participant safety, at the discretion of the PI/designee.

13.5.2. Grade 3 and 4

A participant who develops a Grade 3 or a Grade 4 AE that is either urogenital or product-related should have the product temporarily withheld, in consultation with CONRAD. If a participant develops a Grade 3 or Grade 4 AE that is not urogenital or product-related, the PI/designee must contact CONRAD prior to temporarily withholding product. In either case, the PI/designee must continue the temporary product hold until a recommendation is obtained from CONRAD. If, in consultation with CONRAD, product use is resumed and the same AE recurs at the same grade level at any time during the study, study product must then be permanently discontinued.

14. STATISTICS

14.1. Sample Size Justification

Sample size for this Phase I study is based on feasibility considerations (e.g., cost and time) and not on statistical criteria.

14.2. Analysis Plan

An expanded SAP will be written and approved before scheduled unblinding following data base lock. Any decisions regarding data handling, exclusions from analysis, etc. made subsequent to the approval of this protocol will be documented in the SAP or final study report. Decisions, if any, made by unblinded personnel will be noted.

14.3. General Statistical Issues

Evaluation of Study Objectives: Study objectives will be evaluated by clinical review of descriptive summaries and graphical displays. To supplement clinical judgments, comparisons on selected continuous endpoints between each active group vs. placebo may be estimated from statistical models, with terms for treatment and center as fixed effects and time of measurement as a repeated measure. Comparisons may also be made between the TFV/LNG-ring group vs. the TFV-alone ring group. Estimates and 95% confidence intervals will be provided. It is acknowledged that the trial is not necessarily powered to test hypotheses regarding group differences. Further details on modeling strategy and specifications will be described in the SAP.

Some of the endpoints may require new assay methods in order to obtain meaningful measurements. It is possible that assays for some of the planned endpoints may not be run or that the results may not be statistically analyzed if lab personnel or the CONRAD Study Clinician feel that the measurements are not reliable. These decisions will be made and documented prior to unblinding.

Descriptive Statistics: Categorical variables will be summarized by frequencies and percentages. Continuous variables will be summarized by means, standard deviations, medians, interquartile ranges, minima and maxima. Some PK measurements (e.g., serum concentrations) may fall below the analytic limit of detection and therefore be imputed, if possible, or censored. Summary statistics that include censored observations will be evaluated from medians and other percentiles of the distribution, as censored data can contribute to percentiles of the distributions but not to means and standard deviations.

Imputation: Concentrations that are detectable but below the lower limit of quantification (LLOQ) will be set to one-half the value LLOQ if LLOQ is defined; concentrations below the limit of detection will be set to 0, if limit of detection is defined. Otherwise, no imputation is anticipated. If needed, rules for imputing missing values will be created and will be documented in the analysis plan or statistical report.

Control of Type I Error: This Phase I study is descriptive in nature. P-values and confidence intervals around estimates of treatment differences will not be adjusted for multiple analyses. These statistics are provided to guide clinical judgment; inferences based on statistical significance (or lack thereof) should be made cautiously.

Controlling for Center and Evaluating Center Differences: Baseline socio-demographic statistics, study disposition (completion; reasons for early discontinuation), and descriptive statistics for a few selected key endpoints (to be identified in the SAP) will be computed separately by center to allow review of center differences. Statistical tests will stratify on center when possible.

Definition of Baseline: Baseline is defined as the non-missing measurement obtained closest to but prior to randomization and first insertion of ring at Visit 4.

Subject-Specific Listings: Tables and graphs will be accompanied by subject-specific listings of data as needed.

Classification of Sampling Time Point: Sampling time point will be based on actual time (i.e., allocation errors are ignored). Sampling times within windows if defined in the study manual or SAP will be considered to have occurred at the assigned time. Modifications (e.g., creation of new time points) may be made based on blinded review of actual sampling times and documented in the SAP or statistical report. Samples that fall out of these classifications will not be included in summary statistics based on sampling time point but will be included in other analyses.

14.4. Analysis Populations

A flowchart showing the number of participants screened, enrolled, randomized, completed, contributing to each analysis population, etc. will be reported in accordance with CONSORT guidelines.

The Enrolled Population consists of all participants who undergo genital sampling at Visit 3 whether or not they are randomized or use a study IVR. Reasons why an enrolled participant was not randomized or did not use a study IVR will be listed.

The RP consists of all participants who were randomized and inserted the study IVR at Visit 4. Treatment groups are defined on the basis of treatment assigned. (Allocation errors are ignored.) This will be the primary analysis population for baseline characteristics and study disposition.

The TP is the subset of RP who provided at least some post-insertion safety, PK, or PD data. Treatment groups are defined by treatment received. The TP will be the primary analysis population for all remaining objectives unless TP and RP are identical, in which case the analysis population will be identified as the RP.

The Completer Population is the subset of TP who are considered adherent based on the definition in Section 15.2, and completed the study i.e. provided all key data as defined in the SAP. Some descriptive statistics and graphical displays e.g., concentration data may be repeated on the Completer Population to evaluate data within consistent groups. This will be addressed further in the SAP.

Additional populations or refinements to these definitions, if needed due to unanticipated circumstances, will be described in the SAP or statistical report.

14.5. Interim Analysis

An interim analysis may be conducted after at least 15 participants (both sites combined) have completed the study. The interim analysis is intended to evaluate drug-release parameters to help inform the direction of product development and will consist of descriptive statistics and graphical displays of drug concentrations by treatment group and sampling time point or other descriptive analysis as described in the SAP. While the lead statistician will design the analysis, the analysis will be carried out and verified

by FHI 360 statisticians not otherwise involved in the study (other than, perhaps, as the unblinded randomization statistician). Results for individual participants will not be identified by participant number. Grouped results will not be identified by type of IVR but by “Group A,” “Group B,” etc. It is acknowledged that the nature of the results (e.g., non-zero values) can unblind reviewers to the identity of these groups. Results will be sent via password-protected secure file server to the Director of CONRAD’s Product Development Department or other authorized individual. Recipients of the interim statistical report at CONRAD will maintain appropriate security and confidentiality, and will share the results with other CONRAD staff only on a need-to-know basis. Communication of results with CONRAD staff directly involved in this study will be documented (e.g., who, when, level of detail) and implications, if any, for interpretation of results will be discussed in the clinical study report.

14.6. Evaluation of Primary Objectives

Genital and Systemic Safety

Only treatment-emergent AEs, defined as an AE with onset on or after insertion of IVR, will be included in the analysis; AEs reported on CRFs with onset prior to insertion will be listed. AEs will be summarized separately by treatment group based on MedDRA Preferred Term and System/Organ Class. The number of subjects reporting at least one episode of a given AE category (Preferred Term) as well as number of episodes will be summarized and listed. SAEs, if any, will be identified.

Other than AEs, genital safety endpoints will be summarized separately by treatment group and time of measurement.

Treatment effects on serum chemistries, lipids, CBC, pH, and Nugent score will be evaluated clinically from shift tables reflecting change from baseline based on categories to be developed by the CONRAD Study Clinician and described in the SAP (e.g. Normal→ Abnormal/High [e.g., serum chemistries, lipids, or CBC; Normal→ Intermediate → BV [Nugent score]). Shift tables will be created for each follow-up visit at which measurements were obtained. In addition, changes in pelvic examination results immediately before vs. immediately after IVR insertion at Visit 4 will be listed.

Changes in soluble markers of innate mucosal immunity and inflammatory response in CVL, HIV-1 target immune cell phenotypes and activation/proliferation markers, and cervicovaginal epithelial histology will be evaluated clinically from descriptive statistics by treatment group and time point and supplementary graphical displays (e.g., box-plots). Statistical models comparing treatment groups on these endpoints may be addressed in the SAP.

Separate box-plots for immune markers measured in the presence of semen (as determined by the semen biomarkers) vs. those from the same treatment group and sampling time point without the presence of semen will be created, if necessary, to assist with interpretation of these markers.

Non-iatrogenic colposcopy findings will be described based on characteristics at the first observation, though characteristics at all observations will be listed. Development of ulcerations, abrasions, edema, and other findings will be based on diagnoses made by site colposcopists.

14.7. Evaluation of Secondary Endpoints

14.7.1. Pharmacokinetic Endpoints

After confirming that LNG and TFV levels are as expected (e.g., below level of quantification [BLQ]) for all observations from the placebo group (i.e., no previously undetected allocation errors), this group will be excluded from further PK analysis.

Plasma C_{max}, t_{max} and $AUC_{0\text{-}24h}$ (ln/linear trapezoidal method) from the intensive sampling immediately post insertion will be estimated for each participant from non-compartmental analysis using Phoenix/WinNonLin v6.1 (Pharsight, Inc., Mountain View CA) or higher. Treatment estimates for plasma C_{max} and t_{max} will be based on the group mean and median, respectively. Descriptive statistics of pharmacokinetic parameters will be provided by treatment group and time of measurement. Time x concentration curves associated with ring insertion will be graphed individually by participant, as well as pooled by treatment group.

To evaluate the effect, if any, of product combination, $\log_{10}$-transformed TFV and TFV-DP concentrations may be compared between women receiving the TFV-alone IVR vs. the TFV/LNG IVR. TFV concentrations will be compared using linear mixed models or another appropriate modeling strategy, with treatment group and center as fixed effects and time (visit) as a repeated measure Details regarding modeling method and specification will be provided in the SAP.

Descriptive statistics and graphical displays, as needed, will also be presented by treatment group and visit for trough plasma concentrations and concentrations from other matrices (e.g., vaginal aspirate, genital tissue).

14.8. Evaluation of Tertiary and Exploratory Endpoints

Acceptability will be assessed clinically from (a) early discontinuation for reasons related to study IVR; (b) proportion of women in each IVR group who stop using ring prior to Visit 7; (c) proportion of women in each group who experience spontaneous ring expulsions; and (d) responses to items on the acceptability questionnaire. Key acceptability items will be identified in the SAP. Visible changes to returned rings will be described.

The evaluation of PD and exploratory endpoints will be described in the SAP.

15. MANAGEMENT OF INTERCURRENT EVENTS

15.1. Loss to Follow-up

If a participant fails to appear for a scheduled visit, at least three attempts to contact her should be made over the subsequent 30 days. These attempts should be documented in the participant's study file. The final attempt must be a certified letter to the participant with return-receipt requested. A copy of this letter or documentation of an outreach attempt should be in her file. After these three attempts, no further efforts need be made to find her, but her file should remain open until study closeout.

If the participant does not contact the clinic before the study is closed, the Final Disposition form will be completed at the time of study close-out. The form should indicate that the participant was lost to follow-up. The lost to follow-up designation cannot be made for any participant until the closing date of the study.

15.2. Protocol Adherence

Participants will be considered compliant with the study regimen if they insert the IVR at Visit 4, and keep it in place through Visit 7 with no more than two removals lasting 2 hours or less between Visits 4 and 7. Compliance can also be qualitatively evaluated from PK concentrations; therefore, the site should carefully record the date and time of each collection of PK blood and genital sampling.

15.3. Protocol Violations

If a protocol violation is required to protect the life or physical well-being of a participant in an emergency, it may be carried out without prior approval from CONRAD or the IRB. The investigator must, however, report the violation to CONRAD and the IRB as soon as possible, no later than within 5 working days after the emergency occurred. CONRAD will in turn notify the FDA within 5 working days after receipt of the report.

Other violations from the protocol may not be carried out without prior approval from the IRB if the change involves the rights, safety, or welfare of participants and without prior approval of CONRAD if the change involves the validity of the data, the study's scientific soundness or the rights, safety, or welfare of participants.

If an inadvertent protocol violation has occurred, CONRAD should be notified immediately to determine what steps should be taken.

All protocol violations should be listed in the Protocol Violation Log provided by CONRAD.

15.4. Modification of Protocol

No modification of this protocol may be made without the approval of CONRAD.

16. DIRECT ACCESS TO SOURCE DATA/DOCUMENTS

16.1. Study Monitoring

CONRAD will be responsible for developing the monitoring plan and monitoring the study. A Site Visit Log will be maintained at the site in which all site monitoring visits will be recorded.

Before an investigational site can enter a patient into the study, a representative of CONRAD may visit the investigational study site to:

- Determine the adequacy of the facilities; and/or
- Discuss with the investigator(s) and other personnel their responsibilities with regard to protocol adherence, and the responsibilities of CONRAD or its representatives. This will be documented in a Clinical Study Agreement between CONRAD and the investigator.

During the study, a monitor from CONRAD or representative will have regular contacts with the investigational site, for the following:

- Provide information and support to the investigator(s)
- Confirm that facilities remain acceptable
- Confirm that the investigational team is adhering to the protocol, that data are being accurately recorded in the CRFs, and that investigational product accountability checks are being performed
- Confirm all volunteers have been properly consented
- Perform source data verification (This includes a comparison of the data in the CRFs with the patient's medical records at the hospital or practice, and other records relevant to the study. This will require direct access to all original records for each patient (e.g., clinic charts).)
- Record and report any protocol deviations not previously sent to CONRAD
- Confirm AEs and SAEs have been properly documented on CRFs and confirm any SAEs have been forwarded to CONRAD and those SAEs that met criteria for reporting have been forwarded to the IRB

The monitor will be available between visits if the investigator(s) or other staff needs information or advice.

16.2. Audits and Inspections

Authorized representatives of CONRAD, a regulatory authority, an Independent Ethics Committee or an IRB may visit the site to perform audits or inspections, including source data verification. The purpose of a CONRAD audit or inspection is to systematically and independently examine all study-related activities and documents to determine whether these activities were conducted, and data were recorded, analyzed, and accurately reported according to the protocol, Good Clinical Practice (GCP) guidelines of the International Conference on Harmonization (ICH), and any applicable regulatory requirements.

The investigator may be subject to a field audit by FDA inspectors. This audit could occur while the study is in progress, several years after the study is completed, or when the data are under review by the FDA as part of the new drug approval process. All of the participants' records and other study

documentation must be filed and accessible on short notice (3-5 days) during the study and subsequent retention period. The investigator should contact CONRAD immediately if contacted by a regulatory agency about an inspection.

16.3. Institutional Review Board (IRB)

The PI must obtain IRB approval for the investigation. Initial IRB approval, and all materials approved by the IRB for this study including the patient consent form and recruitment materials must be maintained by the Investigator and made available for inspection.

17. QUALITY CONTROL AND QUALITY ASSURANCE

To ensure compliance with GCP and all applicable regulatory requirements, CONRAD may conduct a quality assurance audit. Please see Section 16.2 for more details regarding the audit process.

18. ETHICS

18.1. Ethics Review

The final study protocol, including the final version of the Informed Consent Form, must be approved or given a favorable opinion in writing by an IRB or IEC as appropriate. The investigator must submit written approval to CONRAD before he or she can enroll any patient/subject into the study. The study must be conducted in accordance with all conditions of approval by the IRB.

The PI is responsible for informing the IRB or IEC of any amendment to the protocol in accordance with local requirements. In addition, the IRB or IEC must approve all advertising used to recruit patients for the study. The protocol must be re-approved by the IRB or IEC upon receipt of amendments and annually, as local regulations require.

The PI is also responsible for providing the IRB with reports of any reportable serious adverse drug reactions from any other study conducted with the investigational product. CONRAD will provide this information to the PI.

Progress reports and notifications of serious adverse drug reactions will be provided to the IRB or IEC according to local regulations and guidelines.

18.2. Ethical Conduct of the Study

The study will be performed in accordance with ethical principles that have their origin in the Declaration of Helsinki and are consistent with ICH GCP, and applicable regulatory requirements. In addition, the PI will follow U.S. Department of Health and Human Services regulations regarding the Health Information Portability and Accountability Act (HIPAA 45, CFR 164). The PI will ensure that appropriate health care or referral is provided for the participants throughout the study.

18.3. Written Informed Consent

18.3.1. Procedure for Obtaining Informed Consent

No volunteer may be admitted into this study until the PI (or designee) has obtained her legally effective informed consent. The PI (or designee) shall seek such consent only under circumstances that provide the prospective participant with sufficient opportunity to consider whether or not to participate in the study. Informed consent must be obtained without coercion, undue influence, or misrepresentation of the potential benefits or risks that might be associated with participation in the study.

Informed consent encompasses all oral and written information given to the volunteer about the study and the study materials. This includes the consent form signed by the participant, the instructions for use of study materials that are provided to the participant, recruitment advertising, and any other information provided to the participant. All such information that is given to the participant will be in a language that is understandable to her. The information will not include any language in which the participant is made to waive any of her rights or which releases or appears to release the PI, the PI’s institution, or CONRAD from liability for negligence.

Informed consent will be documented by the use of a written consent form that is signed by the participant and the PI (or designee). A copy of the signed consent form will be given to each participant. The original signed consent form for each participant will be kept at the site. The consent form must include each of the basic and additional elements of informed consent described in 21 CFR Part 50.25 and must describe each of the risks or discomforts to the participant that have been identified by CONRAD as reasonably foreseeable. CONRAD will provide a sample consent form that meets these requirements. If the PI revises the sample consent form or develops a new one, the new or revised consent form should be submitted to CONRAD for review before it is submitted to the local IRB.

18.3.2. Subject Confidentiality

The confidentiality of all subjects consented into this clinical study will be protected to the fullest extent possible. Subjects' clinic records may be audited by CONRAD staff, FDA personnel, or other individuals authorized in writing by CONRAD to audit the study. However, study subjects will not be identified by name on any CRF, or on any other documentation sent to CONRAD, FHI 360, or other organizations involved in this study, and will not be reported by name in any report or publication resulting from data collected in this study.

19. DATA HANDLING AND RECORDKEEPING

19.1. Method of Data Capture

Clinical data will initially be recorded on source documents at the clinical site. The source documents, including signed informed consent forms, laboratory reports, and participant records, should be maintained at the site, and should be available for review during monitoring visits.

Information from the source documents will then be entered onto CRFs, as outlined in the study manual and the data management plan. CRFs will not be completed for participants who discontinue before Visit 3 genital sampling.

Note that for the Acceptability Questionnaire (completed at Visits 7 pre-removal and/or unscheduled visits), participants may write their responses directly on to the CRF.

Data from central laboratories (including data for explants challenge, inflammatory markers, and PK) will be entered into electronic data sets, provided or approved by FHI 360 as appropriate.

A detailed data management plan will be written before data are collected for this study.

19.2. Inspection of Records

CONRAD will be allowed to conduct site visits to the investigation facilities for the purpose of monitoring any aspect of the study. The PI agrees to allow the monitor to inspect the drug storage area, study drug stocks, drug accountability records, subject charts and study source documents, and other records relative to study conduct.

19.3. Retention of Records

The signed original informed consent documents for each participant and originals of all study documentation (e.g., drug inventory forms, participant clinic records, original laboratory reports, guidebooks) will be retained by the PI for a minimum of 2 years after FDA approval or withdrawal of a New Drug Application (NDA). If an NDA is not submitted within 5 years of the last follow-up visit, the center may request permission in writing from CONRAD to destroy the records. No records may be destroyed without written permission from CONRAD.

20. INVESTIGATOR RESPONSIBILITIES

20.1. Prior to Starting Study

20.1.1. Signing of Investigator's Agreement and Amendments

Prior to study start the PI is responsible for signing and dating the Investigator's Agreement for this study protocol. The signed and dated original must be submitted to CONRAD, and a copy must be maintained by the PI at the site with the study files.

All protocol amendments must be signed and dated by the PI. The signed and dated original must be submitted to CONRAD, and a copy must be maintained by the PI at the site. Amendments must be approved by CONRAD and the IRB before implementation.

20.1.2. Forms and Records

Prior to study initiation, the following forms and records will be provided to CONRAD, and a copy maintained in site files:

- Statement of the Investigator (FDA Form 1572)
- Curriculum vitae (CV) for staff listed on FDA Form 1572:
  - The PI will provide CONRAD with a CV for him/herself showing the education, training, and experience that qualifies him/her as an expert in the area of clinical investigation specific to the product under investigation and his/her affiliation with the site at which the study is being conducted.
  - CVs will also be provided for study staff listed on the 1572 showing the education, training, and experience that qualifies them for their role in the study, and their affiliation with the study site.
- Financial Disclosure Statement (completed by each staff member listed on FDA Form 1572). Note that this form will also need to be provided for each staff person at the end of the study and one year following completion of the study, as possible.
- IRB information consisting of:
  - Name, address, and chairperson of the IRB
  - Multiple Project Assurance (MPA) or Federal-Wide Assurance (FWA) number
  - List of IRB members (names may be withheld in accordance with IRB policy) with title, occupation and affiliation for each member
  - Copy of IRB approval letter for protocol, consent form, advertisement, and other written materials provided to participants
  - Copy of IRB-approved consent form, advertisements, and other written materials provided to participants, as applicable
- Laboratory information, which may include:

- Name of laboratories to be used to process study specimens
- CV of laboratory director(s)
- Current license(s) and/or laboratory certification (such as the Clinical Laboratory Improvement Act [CLIA] certification), with expiration date
- Copy of normal values for tests done by each laboratory for this study

20.2. During the Study: Forms and Records

The following forms and records will be maintained at the study site:

- Investigator's Brochure(s)
- Subject Status Log(s)
- Subject Identification Code List
- Signature and Delegation of Responsibility Log
- Site Visit Log
- Study Supplies Inventory Logs and packing slips
- Protocol Violation Log
- Source documents
- Signed and dated informed consent forms
- CRFs
- IRB documents:
    - Submission and approval letters for protocol and any protocol amendments
    - Submission and approval letters for original and any revised consent forms and any other written material provided to participants
    - Annual submission and approval letters
    - Other IRB correspondence
- Updates on CVs and laboratory information
- General correspondence

Copies of all correspondence between the site and its IRB should be sent to CONRAD.

The PI is responsible for obtaining any updates to these documents, including certification renewals, and sending them to CONRAD in a timely fashion.

20.3. During the Study: Progress Reports

During the study, a monthly report will be sent to CONRAD and will include:

- Participant status
- Protocol violations

- AEs related to study product use or study procedures
- Genital AEs (whether or not related to gel use or study procedures)

Annual progress reports and a final report will be submitted by the site to the IRB or IEC according to local regulations and guidelines.

Throughout the course of the study, the PI will prepare and submit to CONRAD whatever reports are required and detailed in the Subcontract Agreement as applicable.

21. PUBLICATION POLICY

All information concerning the study supplied by CONRAD to the PI and not previously published is considered confidential.

No data collected in this study will be presented or published without prior approval from CONRAD.

22. LIST OF REFERENCES

1. Kott A. Rates of Unintended Pregnancy Remain High In Developing Regions. *International Perspectives on Sexual and Reproductive Health.* 2011;37(1).
2. Singh EJ. Effect of oral contraceptives and IUD's on the copper in human cervical mucus. *Obstet Gynecol.* 1975;45(3):328-330.
3. Singh S. Abortion Worldwide: A Decade of Uneven Progress. In: Wulf D, Hussain R, Bankole A, Sedgh G, eds: Guttmacher Institute; 2009:1-64.
4. UNAIDS. AIDS Epidemic Update, December 2009. 2009:99.
5. Abdool Karim Q, Abdool Karim SS, Frohlich JA, et al. Effectiveness and safety of tenofovir gel, an antiretroviral microbicide, for the prevention of HIV infection in women. *Science.* 2010;329(5996):1168-1174.
6. Burton FG, Skiens WE, Duncan GW. Low-level, progestogen-releasing vaginal contraceptive devices. *Contraception.* 1979;19(5):507-516.
7. Koetsawang S, Ji G, Krishna U, et al. Microdose intravaginal levonorgestrel contraception: a multicentre clinical trial. I. Contraceptive efficacy and side effects. World Health Organization. Task Force on Long-Acting Systemic Agents for Fertility Regulation. *Contraception.* 1990;41(2):105-124.
8. Sahota J, Barnes PM, Mansfield E, Bradley JL, Kirkman RJ. Initial UK experience of the levonorgestrel-releasing contraceptive intravaginal ring. *Adv Contracept.* 1999;15(4):313-324.
9. A randomized, double-blind study of six combined oral contraceptives. *Contraception.* 1982;25(3):231-241.
10. Sheth A, Jain U, Sharma S, et al. A randomized, double-blind study of two combined and two progestogen-only oral contraceptives. *Contraception.* 1982;25(3):243-252.
11. Weisberg E, Fraser IS, Baker J, et al. A randomized comparison of the effects on vaginal and cervical epithelium of a placebo vaginal ring with non-use of a ring. *Contraception.* 2000;62(2):83-89.
