# Supplementary material for: Randomized, placebo controlled phase I trial of safety, pharmacokinetics, pharmacodynamics and acceptability of tenofovir and tenofovir plus levonorgestrel vaginal rings in women
Source: PLoS One. 2018 Jun 28;13(6):e0199778. doi: 10.1371/journal.pone.0199778 (PMC6023238; doi:10.1371/journal.pone.0199778)
Supplement: S1 Data — (ZIP) [file pone.0199778.s006.zip › Demographic Data/Demog.pdf]

Table 14.1.1 Demographics
Randomized Population

	Profamilia DR (853)				EVMS (908)				Overall			
	**TFV+ LNG n (%)**	**TFV Alone n (%)**	**Placebo n (%)**	**Total n (%)**	**TFV+ LNG n (%)**	**TFV Alone n (%)**	**Placebo n (%)**	**Total n (%)**	**TFV+ LNG n (%)**	**TFV Alone n (%)**	**Placebo n (%)**	**Total n (%)**
**Age (years)**												
Mean (SD)	34.9 (4.64)	33.0 (5.14)	34.6 (4.28)	34.1 (4.70)	37.3 (5.24)	32.9 (7.67)	38.0 (5.10)	35.6 (6.53)	36.0 (4.94)	33.0 (6.30)	36.3 (4.79)	34.8 (5.63)
Median	34.0	33.0	35.0	33.0	38.0	33.5	38.0	38.0	35.0	33.0	37.5	34.0
Range	(28.0 to 43.0)	(25.0 to 43.0)	(29.0 to 40.0)	(25.0 to 43.0)	(29.0 to 44.0)	(23.0 to 45.0)	(30.0 to 44.0)	(23.0 to 45.0)	(28.0 to 44.0)	(23.0 to 45.0)	(29.0 to 44.0)	(23.0 to 45.0)
Total	11	11	5	27	9	10	5	24	20	21	10	51
**Ethnicity**												
Hispanic/Latina	11 (100)	11 (100)	5 (100)	27 (100)	1 (11.1)	1 (10.0)	1 (20.0)	3 (12.5)	12 (60.0)	12 (57.1)	6 (60.0)	30 (58.8)
Not Hispanic/Latina	0 (0.0)	0 (0.0)	0 (0.0)	0 (0.0)	8 (88.9)	9 (90.0)	4 (80.0)	21 (87.5)	8 (40.0)	9 (42.9)	4 (40.0)	21 (41.2)
Total	11	11	5	27	9	10	5	24	20	21	10	51
**Race**												
American Indian/Alaska Native	0 (0.0)	0 (0.0)	0 (0.0)	0 (0.0)	0 (0.0)	0 (0.0)	0 (0.0)	0 (0.0)	0 (0.0)	0 (0.0)	0 (0.0)	0 (0.0)
Asian	0 (0.0)	0 (0.0)	0 (0.0)	0 (0.0)	0 (0.0)	0 (0.0)	0 (0.0)	0 (0.0)	0 (0.0)	0 (0.0)	0 (0.0)	0 (0.0)
Native Hawaiian/Pacific Islander	0 (0.0)	0 (0.0)	0 (0.0)	0 (0.0)	0 (0.0)	0 (0.0)	0 (0.0)	0 (0.0)	0 (0.0)	0 (0.0)	0 (0.0)	0 (0.0)
Black or African American	0 (0.0)	0 (0.0)	0 (0.0)	0 (0.0)	4 (44.4)	1 (10.0)	2 (40.0)	7 (29.2)	4 (20.0)	1 (4.8)	2 (20.0)	7 (13.7)
White	0 (0.0)	0 (0.0)	0 (0.0)	0 (0.0)	4 (44.4)	7 (70.0)	2 (40.0)	13 (54.2)	4 (20.0)	7 (33.3)	2 (20.0)	13 (25.5)
More than one race	11 (100)	11 (100)	5 (100)	27 (100)	0 (0.0)	1 (10.0)	0 (0.0)	1 (4.2)	11 (55.0)	12 (57.1)	5 (50.0)	28 (54.9)
Other	0 (0.0)	0 (0.0)	0 (0.0)	0 (0.0)	1 (11.1)	1 (10.0)	1 (20.0)	3 (12.5)	1 (5.0)	1 (4.8)	1 (10.0)	3 (5.9)
Total	11	11	5	27	9	10	5	24	20	21	10	51

Table 14.1.1 Demographics
Randomized Population

	Profamilia DR (853)				EVMS (908)				Overall			
	**TFV+ LNG n (%)**	**TFV Alone n (%)**	**Placebo n (%)**	**Total n (%)**	**TFV+ LNG n (%)**	**TFV Alone n (%)**	**Placebo n (%)**	**Total n (%)**	**TFV+ LNG n (%)**	**TFV Alone n (%)**	**Placebo n (%)**	**Total n (%)**
**Education (years)**												
Mean (SD)	11.2 (4.00)	10.6 (3.04)	12.2 (1.64)	11.1 (3.23)	14.0 (1.41)	15.0 (1.15)	12.8 (1.10)	14.2 (1.46)	12.5 (3.36)	12.7 (3.20)	12.5 (1.35)	12.6 (2.95)
Median	12.0	9.0	12.0	12.0	14.0	14.5	12.0	14.0	13.5	14.0	12.0	13.0
Range	(3.0 to 17.0)	(7.0 to 16.0)	(11.0 to 15.0)	(3.0 to 17.0)	(12.0 to 16.0)	(14.0 to 17.0)	(12.0 to 14.0)	(12.0 to 17.0)	(3.0 to 17.0)	(7.0 to 17.0)	(11.0 to 15.0)	(3.0 to 17.0)
Total	11	11	5	27	9	10	5	24	20	21	10	51
**Partner Status**												
Living with partner	9 (81.8)	9 (81.8)	2 (40.0)	20 (74.1)	3 (33.3)	6 (60.0)	2 (40.0)	11 (45.8)	12 (60.0)	15 (71.4)	4 (40.0)	31 (60.8)
Not living with partner	1 (9.1)	0 (0.0)	3 (60.0)	4 (14.8)	3 (33.3)	1 (10.0)	1 (20.0)	5 (20.8)	4 (20.0)	1 (4.8)	4 (40.0)	9 (17.6)
No partner	1 (9.1)	2 (18.2)	0 (0.0)	3 (11.1)	3 (33.3)	3 (30.0)	2 (40.0)	8 (33.3)	4 (20.0)	5 (23.8)	2 (20.0)	11 (21.6)
Total	11	11	5	27	9	10	5	24	20	21	10	51
