# Supplementary material for: Randomized, placebo controlled phase I trial of safety, pharmacokinetics, pharmacodynamics and acceptability of tenofovir and tenofovir plus levonorgestrel vaginal rings in women
Source: PLoS One. 2018 Jun 28;13(6):e0199778. doi: 10.1371/journal.pone.0199778 (PMC6023238; doi:10.1371/journal.pone.0199778)
Supplement: S1 Data — (ZIP) [file pone.0199778.s006.zip › Demographic Data/Medhist.pdf]

Table 14.1.2 Medical History and Baseline Medical Assessments
Randomized Population

	Profamilia DR (853)				EVMS (908)				Overall			
	TFV+ LNG n (%)	TFV Alone n (%)	Placebo n (%)	Total n (%)	TFV+ LNG n (%)	TFV Alone n (%)	Placebo n (%)	Total n (%)	TFV+ LNG n (%)	TFV Alone n (%)	Placebo n (%)	Total n (%)
**Pregnancy Protection**												
Sexually abstinent	0 (0.0)	0 (0.0)	0 (0.0)	0 (0.0)	5 (55.6)	9 (90.0)	5 (100)	19 (79.2)	5 (25.0)	9 (42.9)	5 (50.0)	19 (37.3)
Sterilization[1]	11 (100)	11 (100)	5 (100)	27 (100)	4 (44.4)	1 (10.0)	0 (0.0)	5 (20.8)	15 (75.0)	12 (57.1)	5 (50.0)	32 (62.7)
Other	0 (0.0)	0 (0.0)	0 (0.0)	0 (0.0)	0 (0.0)	0 (0.0)	0 (0.0)	0 (0.0)	0 (0.0)	0 (0.0)	0 (0.0)	0 (0.0)
Total	11	11	5	27	9	10	5	24	20	21	10	51
**Ever pregnant**												
No	0 (0.0)	0 (0.0)	0 (0.0)	0 (0.0)	0 (0.0)	1 (10.0)	1 (20.0)	2 (8.3)	0 (0.0)	1 (4.8)	1 (10.0)	2 (3.9)
Yes	11 (100)	11 (100)	5 (100)	27 (100)	9 (100)	9 (90.0)	4 (80.0)	22 (91.7)	20 (100)	20 (95.2)	9 (90.0)	49 (96.1)
Total	11	11	5	27	9	10	5	24	20	21	10	51
**Vaginal deliveries[2]**												
Mean (SD)	2.0 (1.18)	2.5 (1.57)	2.6 (0.55)	2.3 (1.27)	1.7 (1.66)	1.2 (1.09)	1.8 (1.26)	1.5 (1.34)	1.9 (1.39)	1.9 (1.48)	2.2 (0.97)	1.9 (1.34)
Median (IQ Range)	2.0 (1.0 to 3.0)	2.0 (2.0 to 4.0)	3.0 (2.0 to 3.0)	2.0 (2.0 to 3.0)	1.0 (0.0 to 3.0)	1.0 (0.0 to 2.0)	2.0 (1.0 to 2.5)	1.5 (0.0 to 2.0)	2.0 (0.5 to 3.0)	2.0 (0.5 to 3.0)	2.0 (2.0 to 3.0)	2.0 (1.0 to 3.0)
Range	(0.0 to 3.0)	(0.0 to 5.0)	(2.0 to 3.0)	(0.0 to 5.0)	(0.0 to 4.0)	(0.0 to 3.0)	(0.0 to 3.0)	(0.0 to 4.0)	(0.0 to 4.0)	(0.0 to 5.0)	(0.0 to 3.0)	(0.0 to 5.0)
Total	11	11	5	27	9	9	4	22	20	20	9	49
**Ceasarean deliveries[2]**												
Mean (SD)	0.9 (1.22)	0.7 (1.01)	0.2 (0.45)	0.7 (1.03)	0.9 (1.05)	0.4 (0.88)	1.0 (0.82)	0.7 (0.94)	0.9 (1.12)	0.6 (0.94)	0.6 (0.73)	0.7 (0.98)
Median (IQ Range)	0.0 (0.0 to 2.0)	0.0 (0.0 to 1.0)	0.0 (0.0 to 0.0)	0.0 (0.0 to 1.0)	0.0 (0.0 to 2.0)	0.0 (0.0 to 0.0)	1.0 (0.5 to 1.5)	0.0 (0.0 to 2.0)	0.0 (0.0 to 2.0)	0.0 (0.0 to 1.0)	0.0 (0.0 to 1.0)	0.0 (0.0 to 1.0)
Range	(0.0 to 3.0)	(0.0 to 3.0)	(0.0 to 1.0)	(0.0 to 3.0)	(0.0 to 2.0)	(0.0 to 2.0)	(0.0 to 2.0)	(0.0 to 2.0)	(0.0 to 3.0)	(0.0 to 3.0)	(0.0 to 2.0)	(0.0 to 3.0)
Total	11	11	5	27	9	9	4	22	20	20	9	49

[1]Of either partner (heterosexual relationship)
[2]Of those ever pregnant.
[3]Other text specifications are provided in LIST_HEALTHPROB.
[4]Updates to medical history text specifications are provided in LIST_NEWMEDHIST.

Table 14.1.2 Medical History and Baseline Medical Assessments
Randomized Population

	Profamilia DR (853)				EVMS (908)				Overall			
	TFV+ LNG n (%)	TFV Alone n (%)	Placebo n (%)	Total n (%)	TFV+ LNG n (%)	TFV Alone n (%)	Placebo n (%)	Total n (%)	TFV+ LNG n (%)	TFV Alone n (%)	Placebo n (%)	Total n (%)
**Weight (lbs)**												
Mean (SD)	134.1 (16.80)	136.8 (19.75)	142.6 (19.07)	136.8 (18.00)	148.8 (23.84)	149.9 (18.89)	166.4 (25.89)	152.9 (22.45)	140.7 (21.07)	143.0 (20.01)	154.5 (24.84)	144.4 (21.60)
Median (IQ Range)	134.0 (124.0 to 152.0)	134.0 (120.0 to 146.0)	140.0 (126.0 to 154.0)	134.0 (124.0 to 150.0)	144.0 (129.0 to 170.0)	147.5 (143.0 to 168.0)	180.0 (142.0 to 184.0)	147.5 (135.0 to 172.5)	134.0 (128.0 to 157.0)	143.0 (129.0 to 151.0)	148.0 (135.0 to 180.0)	140.0 (128.0 to 161.0)
Range	(108.0 to 163.0)	(114.0 to 186.0)	(124.0 to 169.0)	(108.0 to 186.0)	(115.0 to 180.0)	(120.0 to 174.0)	(135.0 to 191.0)	(115.0 to 191.0)	(108.0 to 180.0)	(114.0 to 186.0)	(124.0 to 191.0)	(108.0 to 191.0)
Total	11	11	5	27	9	10	5	24	20	21	10	51
**Height (in)**												
Mean (SD)	62.2 (2.75)	62.8 (3.31)	64.0 (3.54)	62.8 (3.08)	63.9 (2.47)	63.5 (1.65)	66.4 (1.52)	64.3 (2.21)	63.0 (2.70)	63.1 (2.61)	65.2 (2.86)	63.5 (2.78)
Median (IQ Range)	62.0 (60.0 to 64.0)	62.0 (60.0 to 65.0)	63.0 (62.0 to 64.0)	62.0 (61.0 to 64.0)	64.0 (63.0 to 66.0)	64.0 (62.0 to 64.0)	67.0 (66.0 to 67.0)	64.0 (63.0 to 66.0)	63.0 (61.5 to 64.5)	64.0 (62.0 to 64.0)	65.0 (63.0 to 67.0)	64.0 (62.0 to 65.0)
Range	(58.0 to 68.0)	(58.0 to 70.0)	(61.0 to 70.0)	(58.0 to 70.0)	(59.0 to 67.0)	(61.0 to 66.0)	(64.0 to 68.0)	(59.0 to 68.0)	(58.0 to 68.0)	(58.0 to 70.0)	(61.0 to 70.0)	(58.0 to 70.0)
Total	11	11	5	27	9	10	5	24	20	21	10	51
**Systolic blood pressure (mmHg)**												
Mean (SD)	115.7 (9.52)	109.2 (11.41)	115.0 (3.61)	112.9 (9.85)	111.2 (8.50)	114.9 (16.11)	127.0 (14.30)	116.0 (14.07)	113.7 (9.13)	111.9 (13.80)	121.0 (11.69)	114.4 (12.00)
Median (IQ Range)	113.0 (109.0 to 126.0)	108.0 (101.0 to 116.0)	115.0 (114.0 to 116.0)	112.0 (107.0 to 120.0)	110.0 (108.0 to 115.0)	116.0 (110.0 to 124.0)	123.0 (121.0 to 136.0)	115.0 (108.5 to 123.5)	111.5 (108.0 to 122.5)	112.0 (101.0 to 122.0)	118.0 (114.0 to 123.0)	114.0 (108.0 to 123.0)
Range	(102.0 to 129.0)	(95.0 to 131.0)	(110.0 to 120.0)	(95.0 to 131.0)	(99.0 to 124.0)	(90.0 to 141.0)	(109.0 to 146.0)	(90.0 to 146.0)	(99.0 to 129.0)	(90.0 to 141.0)	(109.0 to 146.0)	(90.0 to 146.0)
Total	11	11	5	27	9	10	5	24	20	21	10	51

[1]Of either partner (heterosexual relationship)
[2]Of those ever pregnant.
[3]Other text specifications are provided in LIST_HEALTHPROB.
[4]Updates to medical history text specifications are provided in LIST_NEWMEDHIST.

Table 14.1.2 Medical History and Baseline Medical Assessments
Randomized Population

	Profamilia DR (853)				EVMS (908)				Overall			
	TFV+ LNG n (%)	TFV Alone n (%)	Placebo n (%)	Total n (%)	TFV+ LNG n (%)	TFV Alone n (%)	Placebo n (%)	Total n (%)	TFV+ LNG n (%)	TFV Alone n (%)	Placebo n (%)	Total n (%)
**Diastolic blood pressure (mmHg)**												
Mean (SD)	79.7 (7.06)	71.5 (9.74)	73.6 (4.56)	75.2 (8.60)	69.2 (7.55)	76.1 (7.99)	75.4 (4.28)	73.4 (7.67)	75.0 (8.89)	73.7 (9.05)	74.5 (4.28)	74.4 (8.15)
Median (IQ Range)	81.0 (75.0 to 86.0)	69.0 (63.0 to 79.0)	73.0 (71.0 to 74.0)	75.0 (69.0 to 84.0)	69.0 (64.0 to 71.0)	74.5 (70.0 to 84.0)	77.0 (76.0 to 77.0)	70.5 (68.0 to 79.5)	75.0 (68.5 to 82.5)	70.0 (67.0 to 80.0)	75.0 (71.0 to 77.0)	74.0 (68.0 to 81.0)
Range	(68.0 to 89.0)	(60.0 to 89.0)	(69.0 to 81.0)	(60.0 to 89.0)	(58.0 to 81.0)	(67.0 to 89.0)	(68.0 to 79.0)	(58.0 to 89.0)	(58.0 to 89.0)	(60.0 to 89.0)	(68.0 to 81.0)	(58.0 to 89.0)
Total	11	11	5	27	9	10	5	24	20	21	10	51
**Pelvic exam**												
**Vulva**												
Normal	10 (90.9)	11 (100)	5 (100)	26 (96.3)	9 (100)	10 (100)	5 (100)	24 (100)	19 (95.0)	21 (100)	10 (100)	50 (98.0)
Abnormal	1 (9.1)	0 (0.0)	0 (0.0)	1 (3.7)	0 (0.0)	0 (0.0)	0 (0.0)	0 (0.0)	1 (5.0)	0 (0.0)	0 (0.0)	1 (2.0)
Not done	0 (0.0)	0 (0.0)	0 (0.0)	0 (0.0)	0 (0.0)	0 (0.0)	0 (0.0)	0 (0.0)	0 (0.0)	0 (0.0)	0 (0.0)	0 (0.0)
Total	11	11	5	27	9	10	5	24	20	21	10	51
**Vagina**												
Normal	11 (100)	11 (100)	5 (100)	27 (100)	9 (100)	10 (100)	5 (100)	24 (100)	20 (100)	21 (100)	10 (100)	51 (100)
Abnormal	0 (0.0)	0 (0.0)	0 (0.0)	0 (0.0)	0 (0.0)	0 (0.0)	0 (0.0)	0 (0.0)	0 (0.0)	0 (0.0)	0 (0.0)	0 (0.0)
Not done	0 (0.0)	0 (0.0)	0 (0.0)	0 (0.0)	0 (0.0)	0 (0.0)	0 (0.0)	0 (0.0)	0 (0.0)	0 (0.0)	0 (0.0)	0 (0.0)
Total	11	11	5	27	9	10	5	24	20	21	10	51
**Cervix**												
Normal	11 (100)	11 (100)	5 (100)	27 (100)	9 (100)	10 (100)	5 (100)	24 (100)	20 (100)	21 (100)	10 (100)	51 (100)
Abnormal	0 (0.0)	0 (0.0)	0 (0.0)	0 (0.0)	0 (0.0)	0 (0.0)	0 (0.0)	0 (0.0)	0 (0.0)	0 (0.0)	0 (0.0)	0 (0.0)
Not done	0 (0.0)	0 (0.0)	0 (0.0)	0 (0.0)	0 (0.0)	0 (0.0)	0 (0.0)	0 (0.0)	0 (0.0)	0 (0.0)	0 (0.0)	0 (0.0)
Total	11	11	5	27	9	10	5	24	20	21	10	51

[1]Of either partner (heterosexual relationship)
[2]Of those ever pregnant.
[3]Other text specifications are provided in LIST_HEALTHPROB.
[4]Updates to medical history text specifications are provided in LIST_NEWMEDHIST.

Table 14.1.2 Medical History and Baseline Medical Assessments
Randomized Population

	Profamilia DR (853)				EVMS (908)				Overall			
	TFV+ LNG n (%)	TFV Alone n (%)	Placebo n (%)	Total n (%)	TFV+ LNG n (%)	TFV Alone n (%)	Placebo n (%)	Total n (%)	TFV+ LNG n (%)	TFV Alone n (%)	Placebo n (%)	Total n (%)
**Uterus**												
Normal	11 (100)	11 (100)	5 (100)	27 (100)	9 (100)	10 (100)	5 (100)	24 (100)	20 (100)	21 (100)	10 (100)	51 (100)
Abnormal	0 (0.0)	0 (0.0)	0 (0.0)	0 (0.0)	0 (0.0)	0 (0.0)	0 (0.0)	0 (0.0)	0 (0.0)	0 (0.0)	0 (0.0)	0 (0.0)
Not done	0 (0.0)	0 (0.0)	0 (0.0)	0 (0.0)	0 (0.0)	0 (0.0)	0 (0.0)	0 (0.0)	0 (0.0)	0 (0.0)	0 (0.0)	0 (0.0)
Total	11	11	5	27	9	10	5	24	20	21	10	51
**Adnexa**												
Normal	11 (100)	11 (100)	5 (100)	27 (100)	9 (100)	10 (100)	5 (100)	24 (100)	20 (100)	21 (100)	10 (100)	51 (100)
Abnormal	0 (0.0)	0 (0.0)	0 (0.0)	0 (0.0)	0 (0.0)	0 (0.0)	0 (0.0)	0 (0.0)	0 (0.0)	0 (0.0)	0 (0.0)	0 (0.0)
Not done	0 (0.0)	0 (0.0)	0 (0.0)	0 (0.0)	0 (0.0)	0 (0.0)	0 (0.0)	0 (0.0)	0 (0.0)	0 (0.0)	0 (0.0)	0 (0.0)
Total	11	11	5	27	9	10	5	24	20	21	10	51
**Non-exclusionary health problems or systemic disease**												
**Cardiac**												
No	9 (81.8)	11 (100)	5 (100)	25 (92.6)	8 (88.9)	10 (100)	5 (100)	23 (95.8)	17 (85.0)	21 (100)	10 (100)	48 (94.1)
Yes	2 (18.2)	0 (0.0)	0 (0.0)	2 (7.4)	1 (11.1)	0 (0.0)	0 (0.0)	1 (4.2)	3 (15.0)	0 (0.0)	0 (0.0)	3 (5.9)
Total	11	11	5	27	9	10	5	24	20	21	10	51
**Endocrine**												
No	11 (100)	11 (100)	5 (100)	27 (100)	8 (88.9)	8 (80.0)	4 (80.0)	20 (83.3)	19 (95.0)	19 (90.5)	9 (90.0)	47 (92.2)
Yes	0 (0.0)	0 (0.0)	0 (0.0)	0 (0.0)	1 (11.1)	2 (20.0)	1 (20.0)	4 (16.7)	1 (5.0)	2 (9.5)	1 (10.0)	4 (7.8)
Total	11	11	5	27	9	10	5	24	20	21	10	51

[1]Of either partner (heterosexual relationship)
[2]Of those ever pregnant.
[3]Other text specifications are provided in LIST_HEALTHPROB.
[4]Updates to medical history text specifications are provided in LIST_NEWMEDHIST.

Table 14.1.2 Medical History and Baseline Medical Assessments
Randomized Population

	Profamilia DR (853)				EVMS (908)				Overall			
	TFV+ LNG n (%)	TFV Alone n (%)	Placebo n (%)	Total n (%)	TFV+ LNG n (%)	TFV Alone n (%)	Placebo n (%)	Total n (%)	TFV+ LNG n (%)	TFV Alone n (%)	Placebo n (%)	Total n (%)
**Gastrointestinal**												
No	9 (81.8)	9 (81.8)	5 (100)	23 (85.2)	5 (55.6)	7 (70.0)	3 (60.0)	15 (62.5)	14 (70.0)	16 (76.2)	8 (80.0)	38 (74.5)
Yes	2 (18.2)	2 (18.2)	0 (0.0)	4 (14.8)	4 (44.4)	3 (30.0)	2 (40.0)	9 (37.5)	6 (30.0)	5 (23.8)	2 (20.0)	13 (25.5)
Total	11	11	5	27	9	10	5	24	20	21	10	51
**Urinary tract**												
No	11 (100)	11 (100)	5 (100)	27 (100)	7 (77.8)	9 (90.0)	4 (80.0)	20 (83.3)	18 (90.0)	20 (95.2)	9 (90.0)	47 (92.2)
Yes	0 (0.0)	0 (0.0)	0 (0.0)	0 (0.0)	2 (22.2)	1 (10.0)	1 (20.0)	4 (16.7)	2 (10.0)	1 (4.8)	1 (10.0)	4 (7.8)
Total	11	11	5	27	9	10	5	24	20	21	10	51
**Liver**												
No	11 (100)	11 (100)	5 (100)	27 (100)	9 (100)	10 (100)	5 (100)	24 (100)	20 (100)	21 (100)	10 (100)	51 (100)
Yes	0 (0.0)	0 (0.0)	0 (0.0)	0 (0.0)	0 (0.0)	0 (0.0)	0 (0.0)	0 (0.0)	0 (0.0)	0 (0.0)	0 (0.0)	0 (0.0)
Total	11	11	5	27	9	10	5	24	20	21	10	51
**Respiratory**												
No	10 (90.9)	9 (81.8)	4 (80.0)	23 (85.2)	7 (77.8)	6 (60.0)	5 (100)	18 (75.0)	17 (85.0)	15 (71.4)	9 (90.0)	41 (80.4)
Yes	1 (9.1)	2 (18.2)	1 (20.0)	4 (14.8)	2 (22.2)	4 (40.0)	0 (0.0)	6 (25.0)	3 (15.0)	6 (28.6)	1 (10.0)	10 (19.6)
Total	11	11	5	27	9	10	5	24	20	21	10	51
**Reproductive system**												
No	6 (54.5)	8 (72.7)	3 (60.0)	17 (63.0)	6 (66.7)	2 (20.0)	1 (20.0)	9 (37.5)	12 (60.0)	10 (47.6)	4 (40.0)	26 (51.0)
Yes	5 (45.5)	3 (27.3)	2 (40.0)	10 (37.0)	3 (33.3)	8 (80.0)	4 (80.0)	15 (62.5)	8 (40.0)	11 (52.4)	6 (60.0)	25 (49.0)
Total	11	11	5	27	9	10	5	24	20	21	10	51

[1]Of either partner (heterosexual relationship)
[2]Of those ever pregnant.
[3]Other text specifications are provided in LIST_HEALTHPROB.
[4]Updates to medical history text specifications are provided in LIST_NEWMEDHIST.

Table 14.1.2 Medical History and Baseline Medical Assessments
Randomized Population

	Profamilia DR (853)				EVMS (908)				Overall			
	TFV+ LNG n (%)	TFV Alone n (%)	Placebo n (%)	Total n (%)	TFV+ LNG n (%)	TFV Alone n (%)	Placebo n (%)	Total n (%)	TFV+ LNG n (%)	TFV Alone n (%)	Placebo n (%)	Total n (%)
**Other[3]**												
No	3 (27.3)	5 (45.5)	2 (40.0)	10 (37.0)	3 (33.3)	1 (10.0)	3 (60.0)	7 (29.2)	6 (30.0)	6 (28.6)	5 (50.0)	17 (33.3)
Yes	8 (72.7)	6 (54.5)	3 (60.0)	17 (63.0)	6 (66.7)	9 (90.0)	2 (40.0)	17 (70.8)	14 (70.0)	15 (71.4)	5 (50.0)	34 (66.7)
Total	11	11	5	27	9	10	5	24	20	21	10	51
**Any updates to medical history since last visit**												
**Visit 2[4]**												
No	10 (90.9)	10 (90.9)	5 (100)	25 (92.6)	8 (88.9)	9 (90.0)	5 (100)	22 (91.7)	18 (90.0)	19 (90.5)	10 (100)	47 (92.2)
Yes	1 (9.1)	1 (9.1)	0 (0.0)	2 (7.4)	1 (11.1)	1 (10.0)	0 (0.0)	2 (8.3)	2 (10.0)	2 (9.5)	0 (0.0)	4 (7.8)
Total	11	11	5	27	9	10	5	24	20	21	10	51
**Visit 3[4]**												
No	9 (81.8)	10 (90.9)	4 (80.0)	23 (85.2)	9 (100)	10 (100)	5 (100)	24 (100)	18 (90.0)	20 (95.2)	9 (90.0)	47 (92.2)
Yes	2 (18.2)	1 (9.1)	1 (20.0)	4 (14.8)	0 (0.0)	0 (0.0)	0 (0.0)	0 (0.0)	2 (10.0)	1 (4.8)	1 (10.0)	4 (7.8)
Total	11	11	5	27	9	10	5	24	20	21	10	51

[1]Of either partner (heterosexual relationship)
[2]Of those ever pregnant.
[3]Other text specifications are provided in LIST_HEALTHPROB.
[4]Updates to medical history text specifications are provided in LIST_NEWMEDHIST.
