# Supplementary material for: Randomized, placebo controlled phase I trial of safety, pharmacokinetics, pharmacodynamics and acceptability of tenofovir and tenofovir plus levonorgestrel vaginal rings in women
Source: PLoS One. 2018 Jun 28;13(6):e0199778. doi: 10.1371/journal.pone.0199778 (PMC6023238; doi:10.1371/journal.pone.0199778)
Supplement: S1 Data — (ZIP) [file pone.0199778.s006.zip › Demographic Data/poptree_fig.pdf]

Table 14.1.6.2 Population Tree Flowchart

Enrolled[1]:

Profamilia DR (853)	EVMS (908)	Overall
n (%)	n (%)	n (%)
30 (100.0)	24 (100.0)	54 (100.0)

↓

Randomized[2,3]:

Profamilia DR (853)				EVMS (908)				Overall			
TFV+ LNG	TFV Alone	Placebo	Total	TFV+ LNG	TFV Alone	Placebo	Total	TFV+ LNG	TFV Alone	Placebo	Total
n (%)	n (%)	n (%)	n (%)	n (%)	n (%)	n (%)	n (%)	n (%)	n (%)	n (%)	n (%)
11 (40.7)	11 (40.7)	5 (18.5)	27 (90.0)	9 (37.5)	10 (41.7)	5 (20.8)	24 (100.0)	20 (39.2)	21 (41.2)	10 (19.6)	51 (94.4)

↓

Completers[4]:

Profamilia DR (853)				EVMS (908)				Overall			
TFV+ LNG	TFV Alone	Placebo	Total	TFV+ LNG	TFV Alone	Placebo	Total	TFV+ LNG	TFV Alone	Placebo	Total
n (%)	n (%)	n (%)	n (%)	n (%)	n (%)	n (%)	n (%)	n (%)	n (%)	n (%)	n (%)
11 (42.3)	10 (38.5)	5 (19.2)	26 (86.7)	9 (37.5)	10 (41.7)	5 (20.8)	24 (100.0)	20 (40.0)	20 (40.0)	10 (20.0)	50 (92.6)

The denominator for Total columns is total enrolled for given site or overall. Denominators for treatment-group percentages are the total within the treatment group for the given site or overall.

[1]All participants who undergo genital sampling at Visit 3, whether or not they are randomized or insert a study IVR.

[2]A subset of the Enrolled Population, excluding participants who were either not randomized or did not successfully insert a study IVR at Visit 4.

[3]The Treated Population (excludes participants who fail to provide any valid post-insertion safety, PK, or PD data) is equal to the Randomized Population.

[4]A subset of the Treated Population, and consists of all participants who completed the intended duration of IVR use while adherent to the regimen.
