# Supplementary material for: Randomized, placebo controlled phase I trial of safety, pharmacokinetics, pharmacodynamics and acceptability of tenofovir and tenofovir plus levonorgestrel vaginal rings in women
Source: PLoS One. 2018 Jun 28;13(6):e0199778. doi: 10.1371/journal.pone.0199778 (PMC6023238; doi:10.1371/journal.pone.0199778)
Supplement: S2 Data — (ZIP) [file pone.0199778.s007.zip › PK Data/PC1_TFV.pdf]

Table 14.4.1.1.1.1 Secondary Objective: Descriptive Statistics: Tenofovir Concentrations, by Compartment, Time Point, and Anatomical Location
Completer Population
A. Tenofovir

	Treatment Group		
	**TFV+LNG IVR (N= 20)**	**TFV Alone IVR (N= 20)**	**Overall (N= 40)**
**Compartment, Time Point, and Anatomical Location**			
**Tenofovir**			
**Plasma (ng/ml)**			
**Pre Insertion**			
Mean (SD)	0.2 (0.00)	0.2 (0.00)	0.2 (0.00)
Median (Interquartile Range)	0.2 (0.2 to 0.2)	0.2 (0.2 to 0.2)	0.2 (0.2 to 0.2)
Range (Min to Max)	(0.2 to 0.2)	(0.2 to 0.2)	(0.2 to 0.2)
Total	20	20	40
**1 Hour Post Insertion**			
Mean (SD)	0.2 (0.00)	0.2 (0.00)	0.2 (0.00)
Median (Interquartile Range)	0.2 (0.2 to 0.2)	0.2 (0.2 to 0.2)	0.2 (0.2 to 0.2)
Range (Min to Max)	(0.2 to 0.2)	(0.2 to 0.2)	(0.2 to 0.2)
Total	20	20	40
**2 Hours Post Insertion**			
Mean (SD)	0.2 (0.00)	0.2 (0.00)	0.2 (0.00)
Median (Interquartile Range)	0.2 (0.2 to 0.2)	0.2 (0.2 to 0.2)	0.2 (0.2 to 0.2)
Range (Min to Max)	(0.2 to 0.2)	(0.2 to 0.2)	(0.2 to 0.2)
Total	20	20	40

Note a: Blood draws at Visit 4 (Insertion Visit) are to be obtained at each of 5 time points (pre-insertion, 1, 2, 4, and 8h) for each participant. Visit 4 aspirate and swab samples are obtained post-insertion at only one time point per participant, based on random assignment. Post-IVR removal biopsies were done at either Visit 8 (24h) or Visit 9 (72h) per random assignment. At visits 5 and 7, each participant was to contribute 3 swabs each, taken near the IVR, at the ectocervix, or at introitus.
Note b: Where possible, measurements below the level of quantification (BLQ) are imputed as 0.5*LLOQ (lower limit of quantification)=.155, when LLOQ was defined. With the available data, this was possible only for tenofovir measurements from blood samples.
[1] Some samples were compromised during preparation and are excluded from all analysis.

Table 14.4.1.1.1.1 Secondary Objective: Descriptive Statistics: Tenofovir Concentrations, by Compartment, Time Point, and Anatomical Location
Completer Population
A. Tenofovir

	**Treatment Group**		
	**TFV+LNG IVR (N= 20)**	**TFV Alone IVR (N= 20)**	**Overall (N= 40)**
**4 Hours Post Insertion**			
Mean (SD)	0.2 (0.00)	0.2 (0.08)	0.2 (0.06)
Median (Interquartile Range)	0.2 (0.2 to 0.2)	0.2 (0.2 to 0.2)	0.2 (0.2 to 0.2)
Range (Min to Max)	(0.2 to 0.2)	(0.2 to 0.5)	(0.2 to 0.5)
Total	20	20	40
**8 Hours Post Insertion**			
Mean (SD)	0.4 (0.97)	0.2 (0.04)	0.3 (0.69)
Median (Interquartile Range)	0.2 (0.2 to 0.2)	0.2 (0.2 to 0.2)	0.2 (0.2 to 0.2)
Range (Min to Max)	(0.2 to 4.5)	(0.2 to 0.3)	(0.2 to 4.5)
Total	20	20	40
**Visit 5 : 24 Hour Post Insertion**			
Mean (SD)	0.5 (0.34)	0.7 (0.41)	0.6 (0.39)
Median (Interquartile Range)	0.4 (0.2 to 0.7)	0.6 (0.5 to 0.9)	0.5 (0.3 to 0.8)
Range (Min to Max)	(0.2 to 1.4)	(0.2 to 1.6)	(0.2 to 1.6)
Total	20	20	40

Note a: Blood draws at Visit 4 (Insertion Visit) are to be obtained at each of 5 time points (pre-insertion, 1, 2, 4, and 8h) for each participant. Visit 4 aspirate and swab samples are obtained post-insertion at only one time point per participant, based on random assignment. Post-IVR removal biopsies were done at either Visit 8 (24h) or Visit 9 (72h) per random assignment. At visits 5 and 7, each participant was to contribute 3 swabs each, taken near the IVR, at the ectocervix, or at introitus.
Note b: Where possible, measurements below the level of quantification (BLQ) are imputed as 0.5*LLOQ (lower limit of quantification)=.155, when LLOQ was defined. With the available data, this was possible only for tenofovir measurements from blood samples.
[1] Some samples were compromised during preparation and are excluded from all analysis.

Table 14.4.1.1.1.1 Secondary Objective: Descriptive Statistics: Tenofovir Concentrations, by Compartment, Time Point, and Anatomical Location
Completer Population
A. Tenofovir

	**Treatment Group**		
	**TFV+LNG IVR (N= 20)**	**TFV Alone IVR (N= 20)**	**Overall (N= 40)**
**Visit 6: Ovulation**			
Mean (SD)	2.6 (2.31)	1.3 (0.73)	2.0 (1.81)
Median (Interquartile Range)	2.1 (1.0 to 3.3)	1.2 (0.8 to 1.7)	1.3 (0.9 to 2.9)
Range (Min to Max)	(0.2 to 8.9)	(0.2 to 3.1)	(0.2 to 8.9)
Total	20	20	40
**Visit 7: Pre Removal**			
Mean (SD)	3.4 (4.08)	2.7 (1.34)	3.1 (3.02)
Median (Interquartile Range)	2.0 (1.5 to 3.3)	2.4 (1.9 to 3.3)	2.3 (1.7 to 3.3)
Range (Min to Max)	(0.2 to 17.1)	(0.7 to 6.0)	(0.2 to 17.1)
Total	20	20	40
**Visit 8: 24 Hours Post Removal**			
Mean (SD)	0.3 (0.19)	0.3 (0.14)	0.3 (0.17)
Median (Interquartile Range)	0.2 (0.2 to 0.5)	0.2 (0.2 to 0.4)	0.2 (0.2 to 0.4)
Range (Min to Max)	(0.2 to 0.6)	(0.2 to 0.5)	(0.2 to 0.6)
Total	20	20	40

Note a: Blood draws at Visit 4 (Insertion Visit) are to be obtained at each of 5 time points (pre-insertion, 1, 2, 4, and 8h) for each participant. Visit 4 aspirate and swab samples are obtained post-insertion at only one time point per participant, based on random assignment. Post-IVR removal biopsies were done at either Visit 8 (24h) or Visit 9 (72h) per random assignment. At visits 5 and 7, each participant was to contribute 3 swabs each, taken near the IVR, at the ectocervix, or at introitus.
Note b: Where possible, measurements below the level of quantification (BLQ) are imputed as 0.5*LLOQ (lower limit of quantification)=.155, when LLOQ was defined. With the available data, this was possible only for tenofovir measurements from blood samples.
[1] Some samples were compromised during preparation and are excluded from all analysis.

Table 14.4.1.1.1.1 Secondary Objective: Descriptive Statistics: Tenofovir Concentrations, by Compartment, Time Point, and Anatomical Location
Completer Population
A. Tenofovir

	**Treatment Group**		
	**TFV+LNG IVR (N= 20)**	**TFV Alone IVR (N= 20)**	**Overall (N= 40)**
**Aspirate (ng/ml)**			
**1 Hour Post Insertion**			
Mean (SD)	862.6 (753.37)	1868.8 (1676.92)	1365.7 (1335.40)
Median (Interquartile Range)	671.0 (544.0 to 1006.0)	1819.0 (263.0 to 3100.0)	838.5 (263.0 to 2057.0)
Range (Min to Max)	(35.0 to 2057.0)	(202.0 to 3960.0)	(35.0 to 3960.0)
Total	5	5	10
**2 Hours Post Insertion**			
Mean (SD)	2916.3 (3915.62)	7355.0 (12597.57)	5452.7 (9491.53)
Median (Interquartile Range)	888.0 (431.0 to 7430.0)	1446.5 (568.0 to 14142.0)	888.0 (431.0 to 7430.0)
Range (Min to Max)	(431.0 to 7430.0)	(308.0 to 26219.0)	(308.0 to 26219.0)
Total	3	4	7
**4 Hours Post Insertion**			
Mean (SD)	3719.3 (3281.01)	4362.6 (4557.25)	4076.7 (3812.62)
Median (Interquartile Range)	2956.5 (1172.0 to 6266.5)	3680.0 (153.0 to 7231.0)	3680.0 (933.0 to 7231.0)
Range (Min to Max)	(933.0 to 8031.0)	(149.0 to 10600.0)	(149.0 to 10600.0)
Total	4	5	9

Note a: Blood draws at Visit 4 (Insertion Visit) are to be obtained at each of 5 time points (pre-insertion, 1, 2, 4, and 8h) for each participant. Visit 4 aspirate and swab samples are obtained post-insertion at only one time point per participant, based on random assignment. Post-IVR removal biopsies were done at either Visit 8 (24h) or Visit 9 (72h) per random assignment. At visits 5 and 7, each participant was to contribute 3 swabs each, taken near the IVR, at the ectocervix, or at introitus.
Note b: Where possible, measurements below the level of quantification (BLQ) are imputed as 0.5*LLOQ (lower limit of quantification)=.155, when LLOQ was defined. With the available data, this was possible only for tenofovir measurements from blood samples.
[1] Some samples were compromised during preparation and are excluded from all analysis.

Table 14.4.1.1.1.1 Secondary Objective: Descriptive Statistics: Tenofovir Concentrations, by Compartment, Time Point, and Anatomical Location
Completer Population
A. Tenofovir

	Treatment Group		
	**TFV+LNG IVR (N= 20)**	**TFV Alone IVR (N= 20)**	**Overall (N= 40)**
**8 Hours Post Insertion**			
Mean (SD)	28020.3 (16688.44)	34990.5 (21563.30)	30808.4 (17963.07)
Median (Interquartile Range)	25921.5 (17752.0 to 45544.0)	33597.0 (16450.0 to 53531.0)	25921.5 (16800.0 to 47880.0)
Range (Min to Max)	(5103.0 to 47880.0)	(16100.0 to 56668.0)	(5103.0 to 56668.0)
Total	6	4	10
**Visit 5 : 24 Hour Post Insertion**			
Mean (SD)	509472.4 (332357.50)	412702.5 (293263.27)	459846.8 (312612.66)
Median (Interquartile Range)	477000.0 (233374.0 to 796472.0)	411294.5 (169500.0 to 619904.0)	442589.0 (188000.0 to 717000.0)
Range (Min to Max)	(2800.0 to 1093000.0)	(4207.0 to 1045000.0)	(2800.0 to 1093000.0)
Total	19	20	39
**Visit 6: Ovulation**			
Mean (SD)	2159144.8 (1931441.34)	2198040.4 (2401883.70)	2178592.6 (2151361.15)
Median (Interquartile Range)	1876500.0 (477361.0 to 3025297.0)	1766171.0 (432000.0 to 2780813.0)	1766171.0 (450997.5 to 2956928.5)
Range (Min to Max)	(947.0 to 6907526.0)	(10100.0 to 9989980.0)	(947.0 to 9989980.0)
Total	20	20	40
**Visit 7: Pre Removal**			
Mean (SD)	3257034.0 (5163754.69)	2923738.4 (2729921.02)	3090386.2 (4077486.90)
Median (Interquartile Range)	1690000.0 (450000.0 to 3135000.0)	2580622.0 (596000.0 to 4672662.0)	2145015.0 (596000.0 to 3665600.0)
Range (Min to Max)	(66141.0 to 21156000.0)	(80623.0 to 10577419.0)	(66141.0 to 21156000.0)
Total	19	19	38

Note a: Blood draws at Visit 4 (Insertion Visit) are to be obtained at each of 5 time points (pre-insertion, 1, 2, 4, and 8h) for each participant. Visit 4 aspirate and swab samples are obtained post-insertion at only one time point per participant, based on random assignment. Post-IVR removal biopsies were done at either Visit 8 (24h) or Visit 9 (72h) per random assignment. At visits 5 and 7, each participant was to contribute 3 swabs each, taken near the IVR, at the ectocervix, or at introitus.
Note b: Where possible, measurements below the level of quantification (BLQ) are imputed as 0.5*LLOQ (lower limit of quantification)=.155, when LLOQ was defined. With the available data, this was possible only for tenofovir measurements from blood samples.
[1] Some samples were compromised during preparation and are excluded from all analysis.

Table 14.4.1.1.1.1 Secondary Objective: Descriptive Statistics: Tenofovir Concentrations, by Compartment, Time Point, and Anatomical Location
Completer Population
A. Tenofovir

	Treatment Group		
	**TFV+LNG IVR (N= 20)**	**TFV Alone IVR (N= 20)**	**Overall (N= 40)**
**Visit 8: 24 Hours Post Removal**			
Mean (SD)	149469.5 (285137.22)	150917.6 (168214.45)	150090.1 (238926.82)
Median (Interquartile Range)	14244.0 (4947.0 to 106856.0)	98000.0 (6700.0 to 278067.0)	54600.0 (6053.0 to 189320.0)
Range (Min to Max)	(928.0 to 1138877.0)	(244.0 to 604537.0)	(244.0 to 1138877.0)
Total	20	15	35
**Swab (Near IVR)(ng/mg)**			
**1 Hour Post Insertion**			
Mean (SD)	2.6 (2.97)	10.7 (9.54)	6.7 (7.96)
Median (Interquartile Range)	2.0 (0.2 to 2.9)	8.1 (2.2 to 20.4)	2.5 (1.5 to 8.5)
Range (Min to Max)	(0.2 to 8.3)	(1.3 to 24.3)	(0.2 to 24.3)
Total	6	6	12
**2 Hours Post Insertion**			
Mean (SD)	2.2 (1.64)	2.8 (2.50)	2.6 (2.11)
Median (Interquartile Range)	3.0 (0.4 to 3.3)	2.2 (0.8 to 3.7)	2.6 (0.7 to 3.5)
Range (Min to Max)	(0.4 to 3.3)	(0.7 to 6.7)	(0.4 to 6.7)
Total	3	5	8

Note a: Blood draws at Visit 4 (Insertion Visit) are to be obtained at each of 5 time points (pre-insertion, 1, 2, 4, and 8h) for each participant. Visit 4 aspirate and swab samples are obtained post-insertion at only one time point per participant, based on random assignment. Post-IVR removal biopsies were done at either Visit 8 (24h) or Visit 9 (72h) per random assignment. At visits 5 and 7, each participant was to contribute 3 swabs each, taken near the IVR, at the ectocervix, or at introitus.
Note b: Where possible, measurements below the level of quantification (BLQ) are imputed as 0.5*LLOQ (lower limit of quantification)=.155, when LLOQ was defined. With the available data, this was possible only for tenofovir measurements from blood samples.
[1] Some samples were compromised during preparation and are excluded from all analysis.

Table 14.4.1.1.1.1 Secondary Objective: Descriptive Statistics: Tenofovir Concentrations, by Compartment, Time Point, and Anatomical Location
Completer Population
A. Tenofovir

	Treatment Group		
	**TFV+LNG IVR (N= 20)**	**TFV Alone IVR (N= 20)**	**Overall (N= 40)**
**4 Hours Post Insertion**			
Mean (SD)	11.2 (13.70)	3.9 (5.70)	7.5 (10.62)
Median (Interquartile Range)	6.2 (3.9 to 7.8)	1.4 (0.9 to 2.3)	3.3 (1.4 to 7.8)
Range (Min to Max)	(2.7 to 35.4)	(0.7 to 14.0)	(0.7 to 35.4)
Total	5	5	10
**8 Hours Post Insertion**			
Mean (SD)	29.7 (9.51)	43.9 (13.59)	35.4 (12.86)
Median (Interquartile Range)	28.4 (23.3 to 40.2)	39.6 (34.2 to 53.6)	34.2 (24.7 to 40.6)
Range (Min to Max)	(17.4 to 40.6)	(33.2 to 63.0)	(17.4 to 63.0)
Total	6	4	10
**Visit 5 : 24 Hour Post Insertion**			
Mean (SD)	544.1 (223.24)	507.8 (236.53)	526.0 (227.76)
Median (Interquartile Range)	515.1 (409.1 to 648.6)	480.9 (380.7 to 598.8)	503.5 (398.4 to 638.4)
Range (Min to Max)	(112.0 to 1168.8)	(112.2 to 1138.5)	(112.0 to 1168.8)
Total	20	20	40
**Visit 6: Ovulation**			
Mean (SD)	1849.7 (1150.07)	1697.6 (1283.21)	1773.7 (1205.20)
Median (Interquartile Range)	1647.1 (1008.3 to 2471.5)	1366.7 (738.8 to 2013.3)	1480.6 (803.8 to 2337.1)
Range (Min to Max)	(10.4 to 4455.5)	(407.4 to 4758.1)	(10.4 to 4758.1)
Total	20	20	40

Note a: Blood draws at Visit 4 (Insertion Visit) are to be obtained at each of 5 time points (pre-insertion, 1, 2, 4, and 8h) for each participant. Visit 4 aspirate and swab samples are obtained post-insertion at only one time point per participant, based on random assignment. Post-IVR removal biopsies were done at either Visit 8 (24h) or Visit 9 (72h) per random assignment. At visits 5 and 7, each participant was to contribute 3 swabs each, taken near the IVR, at the ectocervix, or at introitus.
Note b: Where possible, measurements below the level of quantification (BLQ) are imputed as 0.5*LLOQ (lower limit of quantification)=.155, when LLOQ was defined. With the available data, this was possible only for tenofovir measurements from blood samples.
[1] Some samples were compromised during preparation and are excluded from all analysis.

Table 14.4.1.1.1.1 Secondary Objective: Descriptive Statistics: Tenofovir Concentrations, by Compartment, Time Point, and Anatomical Location
Completer Population
A. Tenofovir

	Treatment Group		
	**TFV+LNG IVR (N= 20)**	**TFV Alone IVR (N= 20)**	**Overall (N= 40)**
**Visit 7: Pre Removal**			
Mean (SD)	2867.9 (2436.09)	4228.5 (3569.57)	3548.2 (3094.10)
Median (Interquartile Range)	1907.9 (1471.4 to 3335.8)	3411.3 (3034.8 to 4677.5)	3034.8 (1794.9 to 4502.1)
Range (Min to Max)	(616.7 to 10801.8)	(864.6 to 18289.5)	(616.7 to 18289.5)
Total	20	20	40
**Visit 8: 24 Hours Post Removal**			
Mean (SD)	121.4 (225.68)	134.8 (204.70)	128.0 (212.98)
Median (Interquartile Range)	27.0 (3.4 to 94.8)	17.9 (3.8 to 196.8)	25.0 (3.5 to 137.7)
Range (Min to Max)	(0.1 to 747.9)	(0.7 to 774.2)	(0.1 to 774.2)
Total	20	19	39
**Swab (ECTOCERVIX)(ng/mg)**			
**Visit 5 : 24 Hour Post Insertion**			
Mean (SD)	357.0 (200.41)	304.0 (167.06)	330.5 (184.08)
Median (Interquartile Range)	261.7 (219.8 to 457.8)	301.1 (196.7 to 393.4)	296.1 (219.8 to 437.8)
Range (Min to Max)	(102.5 to 831.8)	(34.4 to 628.5)	(34.4 to 831.8)
Total	20	20	40

Note a: Blood draws at Visit 4 (Insertion Visit) are to be obtained at each of 5 time points (pre-insertion, 1, 2, 4, and 8h) for each participant. Visit 4 aspirate and swab samples are obtained post-insertion at only one time point per participant, based on random assignment. Post-IVR removal biopsies were done at either Visit 8 (24h) or Visit 9 (72h) per random assignment. At visits 5 and 7, each participant was to contribute 3 swabs each, taken near the IVR, at the ectocervix, or at introitus.
Note b: Where possible, measurements below the level of quantification (BLQ) are imputed as 0.5*LLOQ (lower limit of quantification)=.155, when LLOQ was defined. With the available data, this was possible only for tenofovir measurements from blood samples.
[1] Some samples were compromised during preparation and are excluded from all analysis.

Table 14.4.1.1.1.1 Secondary Objective: Descriptive Statistics: Tenofovir Concentrations, by Compartment, Time Point, and Anatomical Location
Completer Population
A. Tenofovir

	Treatment Group		
	**TFV+LNG IVR (N= 20)**	**TFV Alone IVR (N= 20)**	**Overall (N= 40)**
**Visit 7: Pre Removal**			
Mean (SD)	1764.2 (1458.50)	3123.6 (2730.68)	2443.9 (2267.80)
Median (Interquartile Range)	1629.9 (536.6 to 2154.6)	2172.4 (1714.2 to 3350.2)	1961.9 (1173.8 to 2801.6)
Range (Min to Max)	(240.8 to 5833.3)	(361.5 to 11585.4)	(240.8 to 11585.4)
Total	20	20	40
**Swab (Introitus)(ng/mg)**			
**Visit 5 : 24 Hour Post Insertion**			
Mean (SD)	330.6 (249.56)	290.7 (118.96)	310.6 (194.02)
Median (Interquartile Range)	253.6 (170.9 to 402.4)	278.6 (201.5 to 379.1)	261.5 (180.1 to 384.0)
Range (Min to Max)	(108.9 to 1177.2)	(128.8 to 530.3)	(108.9 to 1177.2)
Total	20	20	40

Note a: Blood draws at Visit 4 (Insertion Visit) are to be obtained at each of 5 time points (pre-insertion, 1, 2, 4, and 8h) for each participant. Visit 4 aspirate and swab samples are obtained post-insertion at only one time point per participant, based on random assignment. Post-IVR removal biopsies were done at either Visit 8 (24h) or Visit 9 (72h) per random assignment. At visits 5 and 7, each participant was to contribute 3 swabs each, taken near the IVR, at the ectocervix, or at introitus.
Note b: Where possible, measurements below the level of quantification (BLQ) are imputed as 0.5*LLOQ (lower limit of quantification)=.155, when LLOQ was defined. With the available data, this was possible only for tenofovir measurements from blood samples.
[1] Some samples were compromised during preparation and are excluded from all analysis.

Table 14.4.1.1.1.1 Secondary Objective: Descriptive Statistics: Tenofovir Concentrations, by Compartment, Time Point, and Anatomical Location
Completer Population
A. Tenofovir

	Treatment Group		
	**TFV+LNG IVR (N= 20)**	**TFV Alone IVR (N= 20)**	**Overall (N= 40)**
**Visit 7: Pre Removal**			
Mean (SD)	2123.3 (1884.93)	10985.5 (33447.75)	6787.6 (24417.94)
Median (Interquartile Range)	1328.1 (798.1 to 2650.0)	1418.1 (1202.5 to 3366.9)	1418.1 (853.5 to 3291.7)
Range (Min to Max)	(156.9 to 6150.0)	(318.0 to 149737.0)	(156.9 to 149737.0)
Total	18	20	38
**Tissue (ng/mg)**			
**BXV1 (Near IVR) (ng/mg)**[1]			
**Visit 5: 24 Hours Post Insertion**			
Mean (SD)	95.7 (221.28)	18.9 (14.83)	58.5 (161.58)
Median (Interquartile Range)	15.2 (6.2 to 38.3)	18.0 (7.3 to 25.7)	17.1 (6.4 to 27.7)
Range (Min to Max)	(2.0 to 822.5)	(2.4 to 59.2)	(2.0 to 822.5)
Total	16	15	31
**Visit 7: Pre Removal**			
Mean (SD)	133.5 (190.31)	121.1 (113.70)	127.7 (156.75)
Median (Interquartile Range)	54.1 (32.4 to 146.9)	64.2 (42.4 to 216.1)	57.0 (32.4 to 187.7)
Range (Min to Max)	(9.3 to 759.8)	(23.7 to 402.4)	(9.3 to 759.8)
Total	17	15	32

Note a: Blood draws at Visit 4 (Insertion Visit) are to be obtained at each of 5 time points (pre-insertion, 1, 2, 4, and 8h) for each participant. Visit 4 aspirate and swab samples are obtained post-insertion at only one time point per participant, based on random assignment. Post-IVR removal biopsies were done at either Visit 8 (24h) or Visit 9 (72h) per random assignment. At visits 5 and 7, each participant was to contribute 3 swabs each, taken near the IVR, at the ectocervix, or at introitus.
Note b: Where possible, measurements below the level of quantification (BLQ) are imputed as 0.5*LLOQ (lower limit of quantification)=.155, when LLOQ was defined. With the available data, this was possible only for tenofovir measurements from blood samples.
[1] Some samples were compromised during preparation and are excluded from all analysis.

Table 14.4.1.1.1.1 Secondary Objective: Descriptive Statistics: Tenofovir Concentrations, by Compartment, Time Point, and Anatomical Location
Completer Population
A. Tenofovir

	Treatment Group		
	**TFV+LNG IVR (N= 20)**	**TFV Alone IVR (N= 20)**	**Overall (N= 40)**
**Visit 8: 24 Hours Post Removal**			
Mean (SD)	9.2 (3.46)	22.4 (16.79)	16.2 (13.87)
Median (Interquartile Range)	8.5 (7.1 to 11.7)	14.0 (9.3 to 31.5)	11.2 (8.4 to 15.1)
Range (Min to Max)	(4.2 to 15.1)	(8.1 to 58.1)	(4.2 to 58.1)
Total	8	9	17
**Visit 9: 72 Hours Post Removal**			
Mean (SD)	6.8 (6.47)	6.6 (5.56)	6.7 (5.94)
Median (Interquartile Range)	3.3 (1.2 to 9.5)	5.3 (3.6 to 5.9)	4.5 (2.1 to 9.5)
Range (Min to Max)	(0.6 to 17.3)	(2.1 to 16.2)	(0.6 to 17.3)
Total	9	5	14
**BXV2 (Introitus)(ng/mg)**			
**Visit 5: 24 Hours Post Insertion**			
Mean (SD)	19.0 (15.79)	23.6 (29.14)	21.3 (23.07)
Median (Interquartile Range)	14.7 (7.3 to 31.2)	10.4 (8.7 to 21.7)	11.7 (8.4 to 31.2)
Range (Min to Max)	(3.1 to 58.8)	(1.7 to 103.4)	(1.7 to 103.4)
Total	20	19	39

Note a: Blood draws at Visit 4 (Insertion Visit) are to be obtained at each of 5 time points (pre-insertion, 1, 2, 4, and 8h) for each participant. Visit 4 aspirate and swab samples are obtained post-insertion at only one time point per participant, based on random assignment. Post-IVR removal biopsies were done at either Visit 8 (24h) or Visit 9 (72h) per random assignment. At visits 5 and 7, each participant was to contribute 3 swabs each, taken near the IVR, at the ectocervix, or at introitus.
Note b: Where possible, measurements below the level of quantification (BLQ) are imputed as 0.5*LLOQ (lower limit of quantification)=.155, when LLOQ was defined. With the available data, this was possible only for tenofovir measurements from blood samples.
[1] Some samples were compromised during preparation and are excluded from all analysis.

Table 14.4.1.1.1.1 Secondary Objective: Descriptive Statistics: Tenofovir Concentrations, by Compartment, Time Point, and Anatomical Location
Completer Population
A. Tenofovir

	**Treatment Group**		
	**TFV+LNG IVR (N= 20)**	**TFV Alone IVR (N= 20)**	**Overall (N= 40)**
**Visit 7: Pre Removal**			
Mean (SD)	83.6 (118.42)	89.4 (72.60)	86.4 (97.56)
Median (Interquartile Range)	35.3 (23.7 to 77.8)	71.4 (30.5 to 120.7)	46.7 (25.7 to 110.4)
Range (Min to Max)	(8.2 to 509.5)	(15.5 to 305.2)	(8.2 to 509.5)
Total	20	19	39
**Amount Extracted from Returned Ring (g)**			
**Visit 7**			
Mean (SD)	1.2 (0.12)	1.3 (0.11)	1.2 (0.13)
Median (Interquartile Range)	1.2 (1.1 to 1.3)	1.3 (1.2 to 1.4)	1.2 (1.2 to 1.3)
Range (Min to Max)	(0.9 to 1.4)	(1.2 to 1.5)	(0.9 to 1.5)
Total	20	20	40

Note a: Blood draws at Visit 4 (Insertion Visit) are to be obtained at each of 5 time points (pre-insertion, 1, 2, 4, and 8h) for each participant. Visit 4 aspirate and swab samples are obtained post-insertion at only one time point per participant, based on random assignment. Post-IVR removal biopsies were done at either Visit 8 (24h) or Visit 9 (72h) per random assignment. At visits 5 and 7, each participant was to contribute 3 swabs each, taken near the IVR, at the ectocervix, or at introitus.
Note b: Where possible, measurements below the level of quantification (BLQ) are imputed as 0.5*LLOQ (lower limit of quantification)=.155, when LLOQ was defined. With the available data, this was possible only for tenofovir measurements from blood samples.
[1] Some samples were compromised during preparation and are excluded from all analysis.
