# Supplementary material for: Randomized, placebo controlled phase I trial of safety, pharmacokinetics, pharmacodynamics and acceptability of tenofovir and tenofovir plus levonorgestrel vaginal rings in women
Source: PLoS One. 2018 Jun 28;13(6):e0199778. doi: 10.1371/journal.pone.0199778 (PMC6023238; doi:10.1371/journal.pone.0199778)
Supplement: S2 Data — (ZIP) [file pone.0199778.s007.zip › PK Data/PC2A_TFV.pdf]

Table 14.4.1.1.2. Secondary Objective: Pharmacokinetic Parameters: Tenofovir Concentrations in Plasma
Summary Descriptive Statistics
Completer Population

	Treatment Group		
	**TFV+LNG IVR (N= 20)**	**TFV Alone IVR (N= 20)**	**Overall (N= 40)**
**Pharmacokinetic Parameter:**			
**Concentration 24 Hours Post Insertion (ng/mL)**			
Mean (SD)	0.5 (0.34)	0.7 (0.41)	0.6 (0.39)
Median (Interquartile Range)	0.4 (0.2 to 0.7)	0.6 (0.5 to 0.9)	0.5 (0.3 to 0.8)
Range (Min to Max)	(0.2 to 1.4)	(0.2 to 1.6)	(0.2 to 1.6)
Total	20	20	40
**Concentration Day 15 Post Insertion (ng/mL)**			
Mean (SD)	3.3 (4.56)	1.7 (1.36)	2.5 (3.34)
Median (Interquartile Range)	1.8 (0.4 to 3.0)	1.7 (0.4 to 2.3)	1.8 (0.4 to 2.8)
Range (Min to Max)	(0.2 to 15.5)	(0.2 to 4.7)	(0.2 to 15.5)
Total	13	14	27
**Maximum Concentration (Cmax) (ng/mL)**			
Mean (SD)	4.0 (3.99)	2.8 (1.25)	3.4 (2.98)
Median (Interquartile Range)	3.1 (1.5 to 4.6)	2.5 (2.1 to 3.3)	2.9 (1.9 to 3.8)
Range (Min to Max)	(0.2 to 17.1)	(0.7 to 6.0)	(0.2 to 17.1)
Total	20	20	40

Tenofovir concentrations below the limit of quantification (BLQ) are imputed as .1550, which is 0.5 x lower limit of quantification. Roughly two-thirds (66%) of TFV concentrations were BLQ.
AUCs are calculated using the linear trapesoidal rule / linear imputation up to Cmax and natural log trapesoidal rule / log imputation after Cmax.
Day 15 concentrations reflect the values used in AUC calculations. Day 15 concentrations could not necessarily be imputed for all participants.

Table 14.4.1.1.2. Secondary Objective: Pharmacokinetic Parameters: Tenofovir Concentrations in Plasma
Summary Descriptive Statistics
Completer Population

	Treatment Group		
	**TFV+LNG IVR (N= 20)**	**TFV Alone IVR (N= 20)**	**Overall (N= 40)**
**Time of Maximum Concentration (Tmax)(Days)**			
Mean (SD)	10.4 (5.22)	13.4 (3.65)	11.9 (4.67)
Median (Interquartile Range)	12.0 (6.0 to 14.0)	14.0 (12.0 to 15.5)	13.0 (9.0 to 15.0)
Range (Min to Max)	(0.3 to 18.0)	(4.0 to 18.0)	(0.3 to 18.0)
Total	19	20	39
**Area Under Curve (AUC): 0-24 Hours (ng*day/mL)**			
Mean (SD)	0.3 (0.25)	0.4 (0.15)	0.3 (0.21)
Median (Interquartile Range)	0.2 (0.2 to 0.3)	0.3 (0.3 to 0.4)	0.3 (0.2 to 0.4)
Range (Min to Max)	(0.2 to 1.3)	(0.2 to 0.7)	(0.2 to 1.3)
Total	20	20	40
**Area Under Curve (AUC): 0-15 Days (ng*day/mL)**			
Mean (SD)	38.4 (32.52)	23.0 (6.73)	30.4 (23.92)
Median (Interquartile Range)	33.5 (20.0 to 43.0)	19.7 (17.5 to 28.8)	26.8 (17.5 to 33.6)
Range (Min to Max)	(2.3 to 128.8)	(14.0 to 36.4)	(2.3 to 128.8)
Total	13	14	27

Tenofovir concentrations below the limit of quantification (BLQ) are imputed as .1550, which is 0.5 x lower limit of quantification. Roughly two-thirds (66%) of TFV concentrations were BLQ.
AUCs are calculated using the linear trapesoidal rule / linear imputation up to Cmax and natural log trapesoidal rule / log imputation after Cmax.
Day 15 concentrations reflect the values used in AUC calculations. Day 15 concentrations could not necessarily be imputed for all participants.
