# Supplementary material for: Randomized, placebo controlled phase I trial of safety, pharmacokinetics, pharmacodynamics and acceptability of tenofovir and tenofovir plus levonorgestrel vaginal rings in women
Source: PLoS One. 2018 Jun 28;13(6):e0199778. doi: 10.1371/journal.pone.0199778 (PMC6023238; doi:10.1371/journal.pone.0199778)
Supplement: S2 Data — (ZIP) [file pone.0199778.s007.zip › PK Data/PC4_TFVDP.pdf]

**Table 14.4.1.1.1.2 Secondary Objective: Descriptive Statistics: Tenofovir Diphosphate Concentrations, by Compartment, Time Point, and Anatomical Location. Completer Population**

**B. Tenofovir Diphosphate**

| | Treatment Group | | |
|---|---|---|---|
| | **TFV+LNG IVR (N= 20)** | **TFV Alone IVR (N= 20)** | **Overall (N= 40)** |
| **Compartment, Time Point, and Anatomical Location** | | | |
| **Tenofovir Diphosphate** | | | |
| **BXV1 (Near IVR) (fmol/mg)[1]** | | | |
| **Visit 5: 24 Hours Post Insertion** | | | |
| Mean (SD) | 2037.2 (3341.02) | 674.7 (879.52) | 1401.4 (2568.73) |
| Median (Interquartile Range) | 559.8 (209.6 to 1895.1) | 188.8 (123.8 to 1073.6) | 415.8 (171.3 to 1276.1) |
| Range (Min to Max) | (93.8 to 11578.9) | (33.1 to 3157.0) | (33.1 to 11578.9) |
| Total | 16 | 14 | 30 |
| **Visit 7: Pre Removal** | | | |
| Mean (SD) | 5729.6 (7639.78) | 3130.1 (3158.12) | 4555.6 (6097.54) |
| Median (Interquartile Range) | 2847.7 (821.7 to 6076.9) | 1703.4 (1084.2 to 4647.1) | 2433.3 (1027.9 to 6076.9) |
| Range (Min to Max) | (141.0 to 27283.0) | (112.7 to 10276.6) | (112.7 to 27283.0) |
| Total | 17 | 14 | 31 |
| **Visit 8: 24 Hours Post Removal** | | | |
| Mean (SD) | 672.9 (1152.02) | 2081.2 (2999.82) | 1424.0 (2365.84) |
| Median (Interquartile Range) | 135.8 (64.7 to 675.0) | 1267.2 (94.9 to 2440.6) | 497.6 (68.2 to 1624.6) |
| Range (Min to Max) | (44.1 to 3224.9) | (44.7 to 9000.0) | (44.1 to 9000.0) |
| Total | 7 | 8 | 15 |

**Note: All tenofovir diphosphate measurements in PBMCs were below the limit of quantification and are not reported.**

**[1] This excludes samples compromised during preparation.**

**Table 14.4.1.1.1.2 Secondary Objective: Descriptive Statistics: Tenofovir Diphosphate Concentrations, by Compartment, Time Point, and Anatomical Location. Completer Population**

**B. Tenofovir Diphosphate**

| | Treatment Group | | |
|---|---|---|---|
| | **TFV+LNG IVR (N= 20)** | **TFV Alone IVR (N= 20)** | **Overall (N= 40)** |
| **Visit 9: 72 Hours Post Removal** | | | |
| Mean (SD) | 2267.5 (2419.28) | 997.8 (686.26) | 1814.0 (2036.01) |
| Median (Interquartile Range) | 881.5 (309.7 to 5200.0) | 902.2 (716.6 to 1463.6) | 891.8 (309.7 to 2769.2) |
| Range (Min to Max) | (28.6 to 5445.4) | (65.9 to 1840.5) | (28.6 to 5445.4) |
| Total | 9 | 5 | 14 |
| **BXV2 (Introitus)(fmol/mg)** | | | |
| **Visit 5: 24 Hours Post Insertion** | | | |
| Mean (SD) | 2185.4 (5193.86) | 1043.8 (1210.99) | 1629.3 (3810.11) |
| Median (Interquartile Range) | 345.7 (196.0 to 830.4) | 462.1 (223.9 to 1953.1) | 422.2 (205.1 to 1335.9) |
| Range (Min to Max) | (17.6 to 22233.3) | (111.2 to 4389.8) | (17.6 to 22233.3) |
| Total | 20 | 19 | 39 |
| **Visit 7: Pre Removal** | | | |
| Mean (SD) | 5040.2 (7306.54) | 6387.1 (8120.67) | 5713.6 (7649.75) |
| Median (Interquartile Range) | 2328.1 (403.2 to 7206.4) | 4098.4 (1764.9 to 8807.7) | 3132.5 (1283.2 to 8321.0) |
| Range (Min to Max) | (107.7 to 29909.9) | (232.9 to 36683.9) | (107.7 to 36683.9) |
| Total | 19 | 19 | 38 |

**Note: All tenofovir diphosphate measurements in PBMCs were below the limit of quantification and are not reported.**

**[1] This excludes samples compromised during preparation.**
