# Supplementary material for: Randomized, placebo controlled phase I trial of safety, pharmacokinetics, pharmacodynamics and acceptability of tenofovir and tenofovir plus levonorgestrel vaginal rings in women
Source: PLoS One. 2018 Jun 28;13(6):e0199778. doi: 10.1371/journal.pone.0199778 (PMC6023238; doi:10.1371/journal.pone.0199778)
Supplement: S2 Data — (ZIP) [file pone.0199778.s007.zip › PK Data/PC7_LNG.pdf]

Table 14.4.1.2.1 Secondary Objective: Descriptive Statistics: LNG Concentrations, Sex Hormone Binding Globulin (SHBG) Concentrations and Free LNG Index (FLI) in Plasma and Aspirate, Completer Population by Time Point.

	Treatment Group			
	**TFV+LNG IVR (N= 20)**	**TFV Alone IVR (N= 20)**	**Placebo IVR (N= 10)**	**Overall (N= 50)**
**Levonorgestrel (LNG)**				
**Plasma**				
**LNG CONCENTRATION IN PLASMA (pg/mL)**				
**Pre Insertion[1]**				
**1 Hour Post Insertion**				
Mean (SD)	318.7 (132.31)			318.7 (132.31)
Median (Interquartile Range)	279.0 (223.5 to 361.0)			279.0 (223.5 to 361.0)
Range (Min to Max)	(161.0 to 646.0)			(161.0 to 646.0)
Total	20			20
**2 Hours Post Insertion**				
Mean (SD)	416.5 (140.57)			416.5 (140.57)
Median (Interquartile Range)	386.0 (304.5 to 492.5)			386.0 (304.5 to 492.5)
Range (Min to Max)	(215.0 to 656.0)			(215.0 to 656.0)
Total	20			20
**4 Hours Post Insertion**				
Mean (SD)	493.4 (171.23)			493.4 (171.23)
Median (Interquartile Range)	460.5 (352.5 to 622.5)			460.5 (352.5 to 622.5)
Range (Min to Max)	(212.0 to 786.0)			(212.0 to 786.0)
Total	20			20

Note: TFV Alone and Placebo IVR groups are included in order to report naturally occurring SHBG data for these groups.
[1] All LNG concentrations pre-insertion are null (below level of quantification) and could not be imputed.
[2] A high proportion of LNG concentrations in aspirate are null (below level of quantification) and could not be imputed.

Table 14.4.1.2.1 Secondary Objective: Descriptive Statistics: LNG Concentrations, Sex Hormone Binding Globulin (SHBG) Concentrations and Free LNG Index (FLI) in Plasma and Aspirate, Completer Population by Time Point.

	Treatment Group			
	**TFV+LNG IVR (N= 20)**	**TFV Alone IVR (N= 20)**	**Placebo IVR (N= 10)**	**Overall (N= 50)**
**8 Hours Post Insertion**				
Mean (SD)	520.8 (202.27)			520.8 (202.27)
Median (Interquartile Range)	502.5 (343.0 to 700.5)			502.5 (343.0 to 700.5)
Range (Min to Max)	(204.0 to 854.0)			(204.0 to 854.0)
Total	20			20
**Visit 5 : 24 Hour Post Insertion**				
Mean (SD)	640.1 (208.70)			640.1 (208.70)
Median (Interquartile Range)	588.0 (477.5 to 825.5)			588.0 (477.5 to 825.5)
Range (Min to Max)	(244.0 to 1025.0)			(244.0 to 1025.0)
Total	20			20
**Visit 6: Ovulation**				
Mean (SD)	495.3 (186.80)			495.3 (186.80)
Median (Interquartile Range)	444.0 (350.5 to 648.5)			444.0 (350.5 to 648.5)
Range (Min to Max)	(188.0 to 856.0)			(188.0 to 856.0)
Total	20			20
**Visit 7: Pre Removal**				
Mean (SD)	489.1 (249.39)			489.1 (249.39)
Median (Interquartile Range)	430.0 (321.0 to 589.5)			430.0 (321.0 to 589.5)
Range (Min to Max)	(145.0 to 1067.0)			(145.0 to 1067.0)
Total	20			20

Note: TFV Alone and Placebo IVR groups are included in order to report naturally occurring SHBG data for these groups.
[1] All LNG concentrations pre-insertion are null (below level of quantification) and could not be imputed.
[2] A high proportion of LNG concentrations in aspirate are null (below level of quantification) and could not be imputed.

Table 14.4.1.2.1 Secondary Objective: Descriptive Statistics: LNG Concentrations, Sex Hormone Binding Globulin (SHBG) Concentrations and Free LNG Index (FLI) in Plasma and Aspirate, Completer Population by Time Point.

	Treatment Group			
	**TFV+LNG IVR (N= 20)**	**TFV Alone IVR (N= 20)**	**Placebo IVR (N= 10)**	**Overall (N= 50)**
**Visit 8: 24 Hours Post Removal**				
Mean (SD)	251.9 (145.47)			251.9 (145.47)
Median (Interquartile Range)	215.0 (141.5 to 336.5)			215.0 (141.5 to 336.5)
Range (Min to Max)	(56.0 to 538.0)			(56.0 to 538.0)
Total	20			20
**Aspirate**				
**LNG CONCENTRATION IN ASPIRATE (ng/g)[2]**				
**Visit 6: Ovulation**				
Mean (SD)	1.8 (0.06)			1.8 (0.06)
Median (Interquartile Range)	1.8 (1.7 to 1.8)			1.8 (1.7 to 1.8)
Range (Min to Max)	(1.7 to 1.8)			(1.7 to 1.8)
Total	2			2
**Visit 7: Pre Removal**				
Mean (SD)	2.3 (3.50)			2.3 (3.50)
Median (Interquartile Range)	0.2 (0.1 to 5.3)			0.2 (0.1 to 5.3)
Range (Min to Max)	(0.0 to 9.1)			(0.0 to 9.1)
Total	7			7
**Visit 8: 24 Hours Post Removal**				
Mean (SD)	1.2 (1.86)			1.2 (1.86)
Median (Interquartile Range)	0.1 (0.1 to 3.3)			0.1 (0.1 to 3.3)
Range (Min to Max)	(0.1 to 3.3)			(0.1 to 3.3)
Total	3			3

Note: TFV Alone and Placebo IVR groups are included in order to report naturally occurring SHBG data for these groups.
[1] All LNG concentrations pre-insertion are null (below level of quantification) and could not be imputed.
[2] A high proportion of LNG concentrations in aspirate are null (below level of quantification) and could not be imputed.

Table 14.4.1.2.1 Secondary Objective: Descriptive Statistics: LNG Concentrations, Sex Hormone Binding Globulin (SHBG) Concentrations and Free LNG Index (FLI) in Plasma and Aspirate, Completer Population by Time Point.

	Treatment Group			
	**TFV+LNG IVR (N= 20)**	**TFV Alone IVR (N= 20)**	**Placebo IVR (N= 10)**	**Overall (N= 50)**
**Amount LNG Extracted from Returned Ring (mg)**				
**Visit 7**				
Mean (SD)	4.9 (0.20)			4.9 (0.20)
Median (Interquartile Range)	4.9 (4.7 to 5.0)			4.9 (4.7 to 5.0)
Range (Min to Max)	(4.4 to 5.2)			(4.4 to 5.2)
Total	20			20
**STEROID HORMONE BINDING GLOBULIN (SHBG) (nmol/L)**				
**Pre Insertion**				
Mean (SD)	59.1 (27.16)	67.4 (21.70)	67.4 (34.56)	64.2 (26.53)
Median (Interquartile Range)	51.2 (37.2 to 74.3)	61.3 (53.7 to 84.5)	52.9 (47.3 to 101.0)	57.4 (47.3 to 83.1)
Range (Min to Max)	(24.9 to 117.0)	(27.9 to 115.0)	(28.2 to 128.0)	(24.9 to 128.0)
Total	19	20	10	49
**1 Hour Post Insertion**				
Mean (SD)	58.5 (27.32)	63.6 (18.44)	64.7 (34.36)	61.7 (25.66)
Median (Interquartile Range)	51.6 (39.1 to 69.0)	58.1 (53.3 to 70.0)	51.7 (39.5 to 106.0)	56.4 (45.4 to 72.5)
Range (Min to Max)	(25.7 to 123.0)	(26.8 to 114.0)	(28.3 to 120.0)	(25.7 to 123.0)
Total	20	18	10	48

Note: TFV Alone and Placebo IVR groups are included in order to report naturally occurring SHBG data for these groups.
[1] All LNG concentrations pre-insertion are null (below level of quantification) and could not be imputed.
[2] A high proportion of LNG concentrations in aspirate are null (below level of quantification) and could not be imputed.

Table 14.4.1.2.1 Secondary Objective: Descriptive Statistics: LNG Concentrations, Sex Hormone Binding Globulin (SHBG) Concentrations and Free LNG Index (FLI) in Plasma and Aspirate, Completer Population by Time Point.

	Treatment Group			
	**TFV+LNG IVR (N= 20)**	**TFV Alone IVR (N= 20)**	**Placebo IVR (N= 10)**	**Overall (N= 50)**
**2 Hours Post Insertion**				
Mean (SD)	59.6 (28.56)	67.5 (20.88)	66.4 (37.14)	64.1 (27.44)
Median (Interquartile Range)	50.6 (39.5 to 71.4)	61.1 (55.7 to 84.7)	50.8 (40.7 to 99.8)	57.6 (43.5 to 81.5)
Range (Min to Max)	(25.7 to 120.0)	(25.7 to 109.0)	(29.1 to 135.0)	(25.7 to 135.0)
Total	20	20	10	50
**4 Hours Post Insertion**				
Mean (SD)	59.8 (29.60)	64.7 (19.78)	63.6 (31.57)	62.5 (26.18)
Median (Interquartile Range)	51.4 (37.6 to 72.5)	61.6 (52.6 to 81.5)	52.6 (39.5 to 93.9)	54.6 (45.4 to 77.1)
Range (Min to Max)	(25.1 to 122.0)	(27.9 to 109.0)	(30.2 to 124.0)	(25.1 to 124.0)
Total	20	19	10	49
**8 Hours Post Insertion**				
Mean (SD)	57.9 (29.90)	67.1 (22.83)	66.0 (35.69)	63.3 (28.09)
Median (Interquartile Range)	47.8 (36.7 to 65.5)	58.6 (50.7 to 85.4)	52.9 (38.6 to 94.6)	54.2 (46.0 to 79.4)
Range (Min to Max)	(25.6 to 129.0)	(28.6 to 118.0)	(27.8 to 131.0)	(25.6 to 131.0)
Total	19	20	9	48
**Visit 5 : 24 Hour Post Insertion**				
Mean (SD)	52.6 (25.11)	64.8 (20.32)	62.8 (32.92)	59.8 (25.19)
Median (Interquartile Range)	46.3 (37.1 to 59.9)	57.4 (51.5 to 80.6)	48.4 (38.4 to 102.0)	53.7 (43.9 to 75.7)
Range (Min to Max)	(23.5 to 121.0)	(26.6 to 112.0)	(26.1 to 121.0)	(23.5 to 121.0)
Total	18	20	10	48

Note: TFV Alone and Placebo IVR groups are included in order to report naturally occurring SHBG data for these groups.
[1] All LNG concentrations pre-insertion are null (below level of quantification) and could not be imputed.
[2] A high proportion of LNG concentrations in aspirate are null (below level of quantification) and could not be imputed.

Table 14.4.1.2.1 Secondary Objective: Descriptive Statistics: LNG Concentrations, Sex Hormone Binding Globulin (SHBG) Concentrations and Free LNG Index (FLI) in Plasma and Aspirate, Completer Population by Time Point.

	Treatment Group			
	**TFV+LNG IVR (N= 20)**	**TFV Alone IVR (N= 20)**	**Placebo IVR (N= 10)**	**Overall (N= 50)**
**Visit 6: Ovulation**				
Mean (SD)	47.8 (23.67)	64.9 (22.49)	65.3 (42.19)	58.2 (28.19)
Median (Interquartile Range)	41.1 (28.0 to 65.1)	61.4 (48.4 to 77.5)	44.6 (37.5 to 100.0)	50.8 (38.7 to 77.3)
Range (Min to Max)	(18.5 to 92.7)	(26.5 to 118.0)	(26.0 to 140.0)	(18.5 to 140.0)
Total	19	20	9	48
**Visit 7: Pre Removal**				
Mean (SD)	49.8 (24.27)	69.5 (21.87)	67.4 (42.64)	61.0 (28.80)
Median (Interquartile Range)	49.2 (25.6 to 71.0)	64.8 (54.9 to 85.0)	47.2 (40.0 to 109.0)	57.2 (40.0 to 77.5)
Range (Min to Max)	(15.3 to 94.4)	(31.5 to 119.0)	(25.1 to 145.0)	(15.3 to 145.0)
Total	19	18	9	46
**Visit 8: 24 Hours Post Removal**				
Mean (SD)	46.5 (22.00)	72.2 (24.86)	65.4 (37.43)	61.4 (28.89)
Median (Interquartile Range)	45.1 (26.7 to 65.2)	67.5 (54.3 to 90.1)	48.2 (42.1 to 110.0)	54.8 (42.1 to 77.9)
Range (Min to Max)	(15.9 to 86.9)	(30.2 to 125.0)	(26.7 to 123.0)	(15.9 to 125.0)
Total	17	20	10	47

Note: TFV Alone and Placebo IVR groups are included in order to report naturally occurring SHBG data for these groups.
[1] All LNG concentrations pre-insertion are null (below level of quantification) and could not be imputed.
[2] A high proportion of LNG concentrations in aspirate are null (below level of quantification) and could not be imputed.

Table 14.4.1.2.1 Secondary Objective: Descriptive Statistics: LNG Concentrations, Sex Hormone Binding Globulin (SHBG) Concentrations and Free LNG Index (FLI) in Plasma and Aspirate, Completer Population by Time Point.

	Treatment Group			
	**TFV+LNG IVR (N= 20)**	**TFV Alone IVR (N= 20)**	**Placebo IVR (N= 10)**	**Overall (N= 50)**
**FREE LNG INDEX**				
**1 Hour Post Insertion**				
Mean (SD)	1.9 (0.79)			1.9 (0.79)
Median (Interquartile Range)	2.0 (1.3 to 2.6)			2.0 (1.3 to 2.6)
Range (Min to Max)	(0.8 to 3.5)			(0.8 to 3.5)
Total	20			20
**2 Hours Post Insertion**				
Mean (SD)	2.6 (1.07)			2.6 (1.07)
Median (Interquartile Range)	2.3 (1.6 to 3.6)			2.3 (1.6 to 3.6)
Range (Min to Max)	(1.0 to 4.3)			(1.0 to 4.3)
Total	20			20
**4 Hours Post Insertion**				
Mean (SD)	3.0 (1.34)			3.0 (1.34)
Median (Interquartile Range)	2.7 (2.0 to 4.1)			2.7 (2.0 to 4.1)
Range (Min to Max)	(1.4 to 5.7)			(1.4 to 5.7)
Total	20			20
**8 Hours Post Insertion**				
Mean (SD)	3.2 (1.42)			3.2 (1.42)
Median (Interquartile Range)	2.7 (1.7 to 4.7)			2.7 (1.7 to 4.7)
Range (Min to Max)	(1.5 to 5.7)			(1.5 to 5.7)
Total	19			19

Note: TFV Alone and Placebo IVR groups are included in order to report naturally occurring SHBG data for these groups.
[1] All LNG concentrations pre-insertion are null (below level of quantification) and could not be imputed.
[2] A high proportion of LNG concentrations in aspirate are null (below level of quantification) and could not be imputed.

Table 14.4.1.2.1 Secondary Objective: Descriptive Statistics: LNG Concentrations, Sex Hormone Binding Globulin (SHBG) Concentrations and Free LNG Index (FLI) in Plasma and Aspirate, Completer Population by Time Point.

	Treatment Group			
	**TFV+LNG IVR (N= 20)**	**TFV Alone IVR (N= 20)**	**Placebo IVR (N= 10)**	**Overall (N= 50)**
**Visit 5 : 24 Hour Post Insertion**				
Mean (SD)	4.2 (1.39)			4.2 (1.39)
Median (Interquartile Range)	4.1 (3.2 to 5.6)			4.1 (3.2 to 5.6)
Range (Min to Max)	(2.0 to 6.7)			(2.0 to 6.7)
Total	18			18
**Visit 6: Ovulation**				
Mean (SD)	3.8 (1.57)			3.8 (1.57)
Median (Interquartile Range)	3.5 (2.6 to 4.6)			3.5 (2.6 to 4.6)
Range (Min to Max)	(1.7 to 7.8)			(1.7 to 7.8)
Total	19			19
**Visit 7: Pre Removal**				
Mean (SD)	3.3 (1.23)			3.3 (1.23)
Median (Interquartile Range)	3.0 (2.4 to 3.8)			3.0 (2.4 to 3.8)
Range (Min to Max)	(1.5 to 6.0)			(1.5 to 6.0)
Total	19			19
**Visit 8: 24 Hours Post Removal**				
Mean (SD)	1.7 (0.70)			1.7 (0.70)
Median (Interquartile Range)	1.7 (1.2 to 2.1)			1.7 (1.2 to 2.1)
Range (Min to Max)	(0.6 to 3.2)			(0.6 to 3.2)
Total	17			17

Note: TFV Alone and Placebo IVR groups are included in order to report naturally occurring SHBG data for these groups.
[1] All LNG concentrations pre-insertion are null (below level of quantification) and could not be imputed.
[2] A high proportion of LNG concentrations in aspirate are null (below level of quantification) and could not be imputed.
