# Supplementary material for: Randomized, placebo controlled phase I trial of safety, pharmacokinetics, pharmacodynamics and acceptability of tenofovir and tenofovir plus levonorgestrel vaginal rings in women
Source: PLoS One. 2018 Jun 28;13(6):e0199778. doi: 10.1371/journal.pone.0199778 (PMC6023238; doi:10.1371/journal.pone.0199778)
Supplement: S3 Data — (ZIP) [file pone.0199778.s008.zip › PD Data/PD1_ANTIHIV.pdf]

Table 14.4.2.1.1 Exploratory Pharmacodynamic Endpoint for Tenofovir (Exp 1): Surrogates of Microbial Efficacy. Anti-HIV and Anti-HSV Activities in Cervical Vaginal Fluid
Randomized Population
by Treatment Group and Visit Type of Measurement

	Treatment Group			
	**TFV+LNG IVR (N= 20)**	**TFV Alone IVR (N= 21)**	**Placebo IVR (N= 10)**	**Overall (N= 51)**
**Visit 4 (Pre-Insertion)**				
**Anti-HIV Inhibition(%)[1]**				
Mean (SD)	22.8 (54.26)	38.2 (37.54)	25.3 (36.10)	29.6 (44.39)
Median (Interquartile Range)	44.0 (25.5 to 50.0)	44.0 (25.0 to 54.0)	34.5 (13.0 to 50.0)	43.0 (22.0 to 53.0)
Range (Min to Max)	(-168.0 to 60.0)	(-89.0 to 93.0)	(-65.0 to 55.0)	(-168.0 to 93.0)
Total	20	21	10	51
**Anti-HIV Viability(%)**				
Mean (SD)	90.8 (6.51)	87.7 (9.76)	89.1 (12.15)	89.2 (9.10)
Median (Interquartile Range)	91.5 (85.0 to 95.5)	89.0 (80.0 to 96.0)	95.0 (81.0 to 98.0)	90.0 (83.0 to 98.0)
Range (Min to Max)	(79.0 to 100.0)	(71.0 to 101.0)	(62.0 to 100.0)	(62.0 to 101.0)
Total	20	21	10	51
**Anti-HSV Inhibition(%)**				
Mean (SD)	42.1 (27.57)	43.3 (28.88)	46.1 (34.47)	43.4 (28.88)
Median (Interquartile Range)	38.5 (27.0 to 57.0)	40.0 (16.0 to 63.0)	42.0 (10.0 to 84.0)	40.0 (23.0 to 63.0)
Range (Min to Max)	(-3.0 to 89.0)	(-1.0 to 92.0)	(7.0 to 91.0)	(-3.0 to 92.0)
Total	18	19	9	46

[1] Percentage is the ratio of HIV-1 activity observed in cells grown in the presence of CVLs to that observed in cells grown in presence of HIV-1 alone (laboratory control samples), expressed as a percentage, and may be less than 0. The same holds true for HSV % infectivity.

Table 14.4.2.1.1 Exploratory Pharmacodynamic Endpoint for Tenofovir (Exp 1): Surrogates of Microbial Efficacy. Anti-HIV and Anti-HSV Activities in Cervical Vaginal Fluid
Randomized Population
by Treatment Group and Visit Type of Measurement

	Treatment Group			
	**TFV+LNG IVR (N= 20)**	**TFV Alone IVR (N= 21)**	**Placebo IVR (N= 10)**	**Overall (N= 51)**
**Visit 7 (Pre-Removal)**				
**Anti-HIV Inhibition(%)[1]**				
Mean (SD)	99.6 (0.69)	99.6 (1.39)	23.0 (40.55)	84.2 (35.49)
Median (Interquartile Range)	100.0 (99.0 to 100.0)	100.0 (100.0 to 100.0)	29.0 (25.0 to 38.0)	100.0 (98.0 to 100.0)
Range (Min to Max)	(98.0 to 100.0)	(94.0 to 100.0)	(-77.0 to 81.0)	(-77.0 to 100.0)
Total	20	20	10	50
**Anti-HIV Viability(%)**				
Mean (SD)	92.9 (9.86)	89.7 (9.45)	88.3 (7.93)	90.7 (9.35)
Median (Interquartile Range)	96.5 (84.5 to 100.0)	92.0 (82.5 to 97.5)	84.0 (82.0 to 98.0)	92.0 (82.0 to 100.0)
Range (Min to Max)	(71.0 to 106.0)	(72.0 to 100.0)	(81.0 to 100.0)	(71.0 to 106.0)
Total	20	20	10	50
**Anti-HSV Inhibition(%)**				
Mean (SD)	43.6 (29.81)	39.7 (32.80)	52.4 (30.64)	43.8 (30.88)
Median (Interquartile Range)	52.0 (25.0 to 66.0)	26.0 (14.0 to 77.0)	45.0 (33.0 to 78.0)	38.0 (18.5 to 72.5)
Range (Min to Max)	(-17.0 to 82.0)	(1.0 to 98.0)	(11.0 to 100.0)	(-17.0 to 100.0)
Total	17	18	9	44

[1] Percentage is the ratio of HIV-1 activity observed in cells grown in the presence of CVLs to that observed in cells grown in presence of HIV-1 alone (laboratory control samples), expressed as a percentage, and may be less than 0. The same holds true for HSV % infectivity.
