# Supplementary material for: Randomized, placebo controlled phase I trial of safety, pharmacokinetics, pharmacodynamics and acceptability of tenofovir and tenofovir plus levonorgestrel vaginal rings in women
Source: PLoS One. 2018 Jun 28;13(6):e0199778. doi: 10.1371/journal.pone.0199778 (PMC6023238; doi:10.1371/journal.pone.0199778)
Supplement: S3 Data — (ZIP) [file pone.0199778.s008.zip › PD Data/PD2_HIV1.pdf]

Table 14.4.2.1.2 Exploratory Pharmacodynamic Endpoint for Tenofovir (Exp 1): Surrogates of Microbial Efficacy. Descriptive Statistics Anti-HIV Efficacy in Cervicovaginal Tissue at Baseline (Control Cycle) and IVR Removal (Treatment Cycle) (EVMS Only)
by Treatment Group, Visit, and Day of Ex Vivo Infection Assay
Completer Population

	Treatment Group			
	**TFV+LNG IVR (N= 9)**	**TFV Alone IVR (N= 10)**	**Placebo IVR (N= 5)**	**Overall (N= 24)**
**HIV-1 Infectivity**				
**Visit 3 (End of Control Cycle)**				
**P24: Day 0 (ng/mL*mg)**				
Mean (SD)	0.0001 (0.00033)	0.0004 (0.00052)	0.0004 (0.00073)	0.0003 (0.00050)
Median (Interquartile Range)	0.0000 (0.0000 to 0.0000)	0.0000 (0.0000 to 0.0010)	0.0000 (0.0000 to 0.0002)	0.0000 (0.0000 to 0.0006)
Range (Min to Max)	(0.0000 to 0.0010)	(0.0000 to 0.0010)	(0.0000 to 0.0017)	(0.0000 to 0.0017)
Total	9	10	5	24
**P24: Day 7 (ng/mL*mg)**				
Mean (SD)	0.0089 (0.01060)	0.0490 (0.08517)	0.0359 (0.03341)	0.0312 (0.05838)
Median (Interquartile Range)	0.0100 (0.0000 to 0.0100)	0.0300 (0.0100 to 0.0300)	0.0200 (0.0133 to 0.0462)	0.0167 (0.0100 to 0.0300)
Range (Min to Max)	(0.0000 to 0.0300)	(0.0100 to 0.2900)	(0.0100 to 0.0900)	(0.0000 to 0.2900)
Total	9	10	5	24
**P24: Day 14 (ng/mL*mg)**				
Mean (SD)	0.0150 (0.01871)	0.1157 (0.27019)	0.0246 (0.00640)	0.0590 (0.17638)
Median (Interquartile Range)	0.0100 (0.0000 to 0.0200)	0.0185 (0.0100 to 0.0800)	0.0200 (0.0200 to 0.0295)	0.0200 (0.0100 to 0.0317)
Range (Min to Max)	(0.0000 to 0.0500)	(0.0100 to 0.8800)	(0.0200 to 0.0333)	(0.0000 to 0.8800)
Total	9	10	5	24

Table 14.4.2.1.2 Exploratory Pharmacodynamic Endpoint for Tenofovir (Exp 1): Surrogates of Microbial Efficacy. Descriptive Statistics Anti-HIV Efficacy in Cervicovaginal Tissue at Baseline (Control Cycle) and IVR Removal (Treatment Cycle) (EVMS Only) by Treatment Group, Visit, and Day of Ex Vivo Infection Assay Completer Population

	Treatment Group			
	**TFV+LNG IVR (N= 9)**	**TFV Alone IVR (N= 10)**	**Placebo IVR (N= 5)**	**Overall (N= 24)**
**P24: Day 21 (ng/mL*mg)**				
Mean (SD)	0.0152 (0.01535)	0.4869 (1.15177)	0.0250 (0.02849)	0.2138 (0.75824)
Median (Interquartile Range)	0.0110 (0.0030 to 0.0280)	0.0260 (0.0110 to 0.2800)	0.0133 (0.0100 to 0.0170)	0.0137 (0.0070 to 0.0380)
Range (Min to Max)	(0.0000 to 0.0440)	(0.0040 to 3.7180)	(0.0090 to 0.0756)	(0.0000 to 3.7180)
Total	9	10	5	24
**Proviral DNA Day 21**				
Mean (SD)	1.0 (0.00)	1.0 (0.00)	1.0 (0.00)	1.0 (0.00)
Median (Interquartile Range)	1.0 (1.0 to 1.0)	1.0 (1.0 to 1.0)	1.0 (1.0 to 1.0)	1.0 (1.0 to 1.0)
Range (Min to Max)	(1.0 to 1.0)	(1.0 to 1.0)	(1.0 to 1.0)	(1.0 to 1.0)
Total	5	6	2	13
**Visit 7: Pre Removal**				
**P24: Day 0 (ng/mL*mg)**				
Mean (SD)	0.0001 (0.00033)	0.0004 (0.00052)	0.0004 (0.00073)	0.0003 (0.00050)
Median (Interquartile Range)	0.0000 (0.0000 to 0.0000)	0.0000 (0.0000 to 0.0010)	0.0000 (0.0000 to 0.0002)	0.0000 (0.0000 to 0.0006)
Range (Min to Max)	(0.0000 to 0.0010)	(0.0000 to 0.0010)	(0.0000 to 0.0017)	(0.0000 to 0.0017)
Total	9	10	5	24
**P24: Day 7 (ng/mL*mg)**				
Mean (SD)	0.0155 (0.00878)	0.0375 (0.05042)	0.0326 (0.03812)	0.0282 (0.03713)
Median (Interquartile Range)	0.0200 (0.0100 to 0.0200)	0.0200 (0.0100 to 0.0400)	0.0200 (0.0100 to 0.0231)	0.0200 (0.0100 to 0.0215)
Range (Min to Max)	(0.0000 to 0.0298)	(0.0047 to 0.1700)	(0.0100 to 0.1000)	(0.0000 to 0.1700)
Total	9	10	5	24

Table 14.4.2.1.2 Exploratory Pharmacodynamic Endpoint for Tenofovir (Exp 1): Surrogates of Microbial Efficacy. Descriptive Statistics Anti-HIV Efficacy in Cervicovaginal Tissue at Baseline (Control Cycle) and IVR Removal (Treatment Cycle) (EVMS Only) by Treatment Group, Visit, and Day of Ex Vivo Infection Assay Completer Population

	Treatment Group			
	**TFV+LNG IVR (N= 9)**	**TFV Alone IVR (N= 10)**	**Placebo IVR (N= 5)**	**Overall (N= 24)**
**P24: Day 14 (ng/mL*mg)**				
Mean (SD)	0.0114 (0.00706)	0.0212 (0.01827)	0.0240 (0.02862)	0.0181 (0.01788)
Median (Interquartile Range)	0.0100 (0.0100 to 0.0100)	0.0185 (0.0100 to 0.0300)	0.0167 (0.0100 to 0.0200)	0.0120 (0.0100 to 0.0200)
Range (Min to Max)	(0.0000 to 0.0246)	(0.0000 to 0.0600)	(0.0000 to 0.0733)	(0.0000 to 0.0733)
Total	9	10	5	24
**P24: Day 21 (ng/mL*mg)**				
Mean (SD)	0.0160 (0.01021)	0.0155 (0.00657)	0.0599 (0.10631)	0.0249 (0.04852)
Median (Interquartile Range)	0.0130 (0.0130 to 0.0200)	0.0170 (0.0090 to 0.0200)	0.0140 (0.0100 to 0.0154)	0.0145 (0.0098 to 0.0200)
Range (Min to Max)	(0.0000 to 0.0333)	(0.0070 to 0.0250)	(0.0100 to 0.2500)	(0.0000 to 0.2500)
Total	9	10	5	24
**Proviral DNA Day 21**				
Mean (SD)	8.2 (18.33)	0.7 (0.97)	4.0 (3.65)	4.1 (11.25)
Median (Interquartile Range)	0.0 (0.0 to 0.1)	0.2 (0.1 to 0.8)	4.0 (1.4 to 6.5)	0.1 (0.0 to 1.4)
Range (Min to Max)	(0.0 to 41.0)	(0.0 to 2.5)	(1.4 to 6.5)	(0.0 to 41.0)
Total	5	6	2	13
