# Supplementary material for: Randomized, placebo controlled phase I trial of safety, pharmacokinetics, pharmacodynamics and acceptability of tenofovir and tenofovir plus levonorgestrel vaginal rings in women
Source: PLoS One. 2018 Jun 28;13(6):e0199778. doi: 10.1371/journal.pone.0199778 (PMC6023238; doi:10.1371/journal.pone.0199778)
Supplement: S3 Data — (ZIP) [file pone.0199778.s008.zip › PD Data/PD3_LNG1.pdf]

Table 14.4.2.2.1 Tertiary Endpoints: Surrogates of Contraceptive Efficacy: Cervical Mucus Assessment, Sperm Migration, Ovulation, Serum Estradiol Concentrations, Overall Protection Against Pregnancy
Randomized Population.
by Treatment Group and Visit Type

	Treatment Group			
	**TFV+LNG IVR (N= 20)**	**TFV Alone IVR (N= 21)**	**Placebo IVR (N= 10)**	**Overall (N= 51)**
**Visit 6: Cervical Mucus Score (0-15)**				
Poor (<= 10)	19 (100)	9 (45.0)	4 (40.0)	32 (65.3)
Good (> 10)	0 (0.0)	11 (55.0)	6 (60.0)	17 (34.7)
<7	14 (73.7)	7 (35.0)	1 (10.0)	22 (44.9)
7-10	5 (26.3)	2 (10.0)	3 (30.0)	10 (20.4)
>10	0 (0.0)	11 (55.0)	6 (60.0)	17 (34.7)
Mean (SD)	4.4 (2.71)	9.6 (4.03)	10.2 (2.66)	7.7 (4.20)
Median (Interquartile Range)	3.0 (2.0 to 7.0)	11.5 (5.5 to 13.0)	11.0 (9.0 to 12.0)	7.0 (4.0 to 12.0)
Range (Min to Max)	(1.0 to 9.0)	(3.0 to 15.0)	(5.0 to 13.0)	(1.0 to 15.0)
Total	19	20	10	49
**Visit 6: Simplified Slide Test[1]**				
ABNORMAL RESULTS	5 (35.7)	6 (31.6)	0 (0.0)	11 (26.2)
NORMAL RESULTS	3 (21.4)	10 (52.6)	8 (88.9)	21 (50.0)
PENETRATION WITH POOR MOTALITY	0 (0.0)	1 (5.3)	0 (0.0)	1 (2.4)
PENETRATION WITH POOR MOTILITY.	1 (7.1)	0 (0.0)	0 (0.0)	1 (2.4)
POOR RESULTS	5 (35.7)	2 (10.5)	1 (11.1)	8 (19.0)
Total	14	19	9	42

[1] Simplified Slide Test results incorporate verbatim text from an other/specify field.
[2] Protected from Pregnancy defined as: (1) did not ovulate or (2) had either (a) an abnormal simplified slide test result or (b) a poor cervical mucus score.

Table 14.4.2.2.1 Tertiary Endpoints: Surrogates of Contraceptive Efficacy: Cervical Mucus Assessment, Sperm Migration, Ovulation, Serum Estradiol Concentrations, Overall Protection Against Pregnancy
Randomized Population.
by Treatment Group and Visit Type

	Treatment Group			
	**TFV+LNG IVR (N= 20)**	**TFV Alone IVR (N= 21)**	**Placebo IVR (N= 10)**	**Overall (N= 51)**
**Visit 7: Pre-Removal P4 (ng/ml)**				
< 3 ng/ml	11 (55.0)	5 (25.0)	3 (30.0)	19 (38.0)
>= 3 ng/ml	9 (45.0)	15 (75.0)	7 (70.0)	31 (62.0)
Mean (SD)	3.9 (3.60)	8.2 (5.34)	7.8 (6.44)	6.4 (5.30)
Median (Interquartile Range)	2.8 (1.0 to 5.7)	9.1 (2.9 to 11.6)	7.6 (0.6 to 11.7)	6.3 (1.0 to 10.8)
Range (Min to Max)	(0.3 to 13.5)	(0.3 to 17.9)	(0.2 to 19.8)	(0.2 to 19.8)
Total	20	20	10	50
**Estradiol (pg/ml)**				
**Visit 4: Pre Insertion**				
Mean (SD)	70.1 (51.83)	52.5 (24.94)	51.5 (35.04)	59.7 (40.11)
Median (Interquartile Range)	57.0 (30.0 to 91.0)	46.0 (34.0 to 64.0)	34.0 (30.0 to 62.0)	47.0 (31.0 to 73.0)
Range (Min to Max)	(24.0 to 227.0)	(19.0 to 110.0)	(29.0 to 131.0)	(19.0 to 227.0)
Total	18	17	8	43
**Visit 6: Ovulation**				
Mean (SD)	207.5 (157.72)	157.7 (107.90)	143.0 (107.26)	174.7 (130.51)
Median (Interquartile Range)	162.0 (71.5 to 311.0)	142.5 (62.5 to 230.5)	149.5 (33.0 to 203.0)	147.0 (67.0 to 253.0)
Range (Min to Max)	(39.0 to 582.0)	(26.0 to 380.0)	(28.0 to 341.0)	(26.0 to 582.0)
Total	20	20	10	50

[1] Simplified Slide Test results incorporate verbatim text from an other/specify field.
[2] Protected from Pregnancy defined as: (1) did not ovulate or (2) had either (a) an abnormal simplified slide test result or (b) a poor cervical mucus score.

Table 14.4.2.2.1 Tertiary Endpoints: Surrogates of Contraceptive Efficacy: Cervical Mucus Assessment, Sperm Migration, Ovulation, Serum Estradiol Concentrations, Overall Protection Against Pregnancy
Randomized Population.
by Treatment Group and Visit Type

	Treatment Group			
	**TFV+LNG IVR (N= 20)**	**TFV Alone IVR (N= 21)**	**Placebo IVR (N= 10)**	**Overall (N= 51)**
**Visit 7: Pre Removal**				
Mean (SD)	225.4 (208.67)	121.5 (69.09)	122.4 (81.72)	163.2 (150.30)
Median (Interquartile Range)	165.5 (93.0 to 258.0)	110.5 (76.0 to 134.0)	97.5 (54.0 to 186.0)	124.0 (80.0 to 203.0)
Range (Min to Max)	(27.0 to 873.0)	(38.0 to 307.0)	(25.0 to 272.0)	(25.0 to 873.0)
Total	20	20	10	50
**Protected from Pregnancy[2]**				
**Visit 7: Pre Removal**				
NO	0 (0.0)	10 (50.0)	5 (50.0)	15 (30.0)
YES	20 (100)	10 (50.0)	5 (50.0)	35 (70.0)
Total	20	20	10	50

[1] **Simplified Slide Test results incorporate verbatim text from an other/specify field.**
[2] **Protected from Pregnancy defined as: (1) did not ovulate or (2) had either (a) an abnormal simplified slide test result or (b) a poor cervical mucus score.**
