# Supplementary material for: Randomized, placebo controlled phase I trial of safety, pharmacokinetics, pharmacodynamics and acceptability of tenofovir and tenofovir plus levonorgestrel vaginal rings in women
Source: PLoS One. 2018 Jun 28;13(6):e0199778. doi: 10.1371/journal.pone.0199778 (PMC6023238; doi:10.1371/journal.pone.0199778)
Supplement: S4 Data — (ZIP) [file pone.0199778.s009.zip › Accept Adhere Data/A1_ACCEPT.pdf]

Table 14.4.4.1 Responses to Acceptability Questionnaire at IVR Removal, Completer Population by Treatment Group

	Treatment Group						Comparisons Between Treatment Group		
	TFV+LNG IVR (N= 20)		TFV Alone IVR (N= 20)		Placebo IVR (N= 10)		TFV+LNG vs TFV Alone	TFV+LNG vs Placebo	TFV Alone vs Placebo
	n	%	n	%	n	%	p-value[1]	p-value	p-value
**Ease of Insertion**							0.4998	0.3068	0.3816
Very Easy	13	(68.4)	14	(70.0)	8	(88.9)			
Fairly Easy	4	(21.1)	6	(30.0)	1	(11.1)			
Neutral	1	(5.3)	0	(0.0)	0	(0.0)			
Somewhat difficult	1	(5.3)	0	(0.0)	0	(0.0)			
Very difficult	0	(0.0)	0	(0.0)	0	(0.0)			
Total	19		20		9				
**Worries you may have about using the vaginal ring:**[2]									
The ring coming out by accident	4	(20.0)	6	(30.0)	4	(44.4)	0.7164	0.2089	0.6749
The ring not staying correctly in place	3	(15.0)	5	(25.0)	4	(44.4)	0.6948	0.1581	0.3962
The ring getting stuck inside your body	3	(15.0)	0	(0.0)	1	(11.1)	0.2308	1.0000	0.3103
Difficulty inserting the ring	1	(5.0)	1	(5.0)	0	(0.0)	1.0000	1.0000	1.0000
Difficulty removing the ring	2	(10.0)	0	(0.0)	0	(0.0)	0.4872	1.0000	
The ring feeling uncomfortable or painful during your normal daily activities	3	(15.0)	0	(0.0)	1	(11.1)	0.2308	1.0000	0.3103
The ring causing infection, infertility or other health problems	3	(15.0)	0	(0.0)	1	(11.1)	0.2308	1.0000	0.3103
Feeling sick from wearing the ring	1	(5.0)	1	(5.0)	0	(0.0)	1.0000	1.0000	1.0000
Any other worries	1	(5.0)	2	(10.0)	0	(0.0)	1.0000	1.0000	1.0000

[1] **P-values are from two-sided Mantel-Haenszel Chi-square exact test (ordinal variables) or Fisher's exact test (nominal variables).**

[2] **Solicited response categories.**

[3] **'Would you like it if you could tell, just by looking at the vaginal ring, that while it was in your vagina, it had been releasing medicine to prevent HIV and/or pregnancy?'**

[4] **Please see the acceptability listing for details.**

Table 14.4.4.1 Responses to Acceptability Questionnaire at IVR Removal, Completer Population by Treatment Group

	Treatment Group						Comparisons Between Treatment Group		
	TFV+LNG IVR (N= 20)		TFV Alone IVR (N= 20)		Placebo IVR (N= 10)		TFV+LNG vs TFV Alone	TFV+LNG vs Placebo	TFV Alone vs Placebo
	n	%	n	%	n	%	p-value[1]	p-value	p-value
**Worries you MAY have in the future about using the ring to prevent HIV infection and/or pregnancy:[2]**									
The ring coming out during sex	9	(45.0)	6	(30.0)	4	(44.4)	0.5145	1.0000	0.6749
The ring feeling uncomfortable or painful during sex.	7	(35.0)	8	(40.0)	3	(33.3)	1.0000	1.0000	1.0000
Partner feeling the ring during sex	11	(55.0)	7	(35.0)	5	(55.6)	0.3406	1.0000	0.4223
Using the ring during menses	4	(20.0)	4	(20.0)	2	(22.2)	1.0000	1.0000	1.0000
The type of protection you normally use during menses									
Pads	15	(75.0)	12	(60.0)	5	(55.6)			
Tampons	2	(10.0)	6	(30.0)	2	(22.2)			
Both pads and tampons	2	(10.0)	1	(5.0)	2	(22.2)			
Other, specify	1	(5.0)	1	(5.0)	0	(0.0)			
Total	20		20		9				
Any other worries	1	(5.0)	1	(5.0)	0	(0.0)	1.0000	1.0000	1.0000

[1] P-values are from two-sided Mantel-Haenszel Chi-square exact test (ordinal variables) or Fisher's exact test (nominal variables).
[2] Solicited response categories.
[3] 'Would you like it if you could tell, just by looking at the vaginal ring, that while it was in your vagina, it had been releasing medicine to prevent HIV and/or pregnancy?'
[4] Please see the acceptability listing for details.

Table 14.4.4.1 Responses to Acceptability Questionnaire at IVR Removal, Completer Population by Treatment Group

	Treatment Group						Comparisons Between Treatment Group		
	TFV+LNG IVR (N= 20)		TFV Alone IVR (N= 20)		Placebo IVR (N= 10)		TFV+LNG vs TFV Alone	TFV+LNG vs Placebo	TFV Alone vs Placebo
	n	%	n	%	n	%	p-value[1]	p-value	p-value
**Aware of ring during daily activities**							0.4872	0.7783	
Never	18	(90.0)	20	(100)	9	(100)			
Sometimes	1	(5.0)	0	(0.0)	0	(0.0)			
Most of the time	1	(5.0)	0	(0.0)	0	(0.0)			
Total	20		20		9				
**Comfort during use**							1.0000	1.0000	
Usually comfortable	19	(95.0)	20	(100)	9	(100)			
Sometimes comfortable and sometimes uncomfortable	1	(5.0)	0	(0.0)	0	(0.0)			
Usually uncomfortable	0	(0.0)	0	(0.0)	0	(0.0)			
Total	20		20		9				
**How worried are you that you might get infected with HIV in the future?**							0.7834	0.4649	0.7272
Very worried	1	(5.0)	1	(5.0)	0	(0.0)			
Somewhat worried	3	(15.0)	5	(25.0)	4	(44.4)			
Not worried at all	16	(80.0)	14	(70.0)	5	(55.6)			
Total	20		20		9				

[1] P-values are from two-sided Mantel-Haenszel Chi-square exact test (ordinal variables) or Fisher's exact test (nominal variables).
[2] Solicited response categories.
[3] 'Would you like it if you could tell, just by looking at the vaginal ring, that while it was in your vagina, it had been releasing medicine to prevent HIV and/or pregnancy?'
[4] Please see the acceptability listing for details.

Table 14.4.4.1 Responses to Acceptability Questionnaire at IVR Removal, Completer Population by Treatment Group

	Treatment Group						Comparisons Between Treatment Group		
	TFV+LNG IVR (N= 20)		TFV Alone IVR (N= 20)		Placebo IVR (N= 10)		TFV+LNG vs TFV Alone	TFV+LNG vs Placebo	TFV Alone vs Placebo
	n	%	n	%	n	%	p-value[1]	p-value	p-value
**If a vaginal ring provided some protection against HIV, how likely would you be to keep it inserted in your vagina every day?**							1.0000	0.6119	0.5881
Very unlikely	1	(5.0)	1	(5.0)	0	(0.0)			
Unlikely	2	(10.0)	1	(5.0)	0	(0.0)			
Likely	5	(25.0)	7	(35.0)	7	(77.8)			
Very likely	12	(60.0)	11	(55.0)	2	(22.2)			
Total	20		20		9				
**If a vaginal ring provided some protection against HIV and protected against pregnancy, how likely would you be to keep it inserted in your vagina every day?**							1.0000	1.0000	1.0000
Very unlikely	0	(0.0)	1	(5.0)	0	(0.0)			
Unlikely	2	(10.0)	1	(5.0)	0	(0.0)			
Likely	8	(40.0)	7	(35.0)	5	(55.6)			
Very likely	10	(50.0)	11	(55.0)	4	(44.4)			
Total	20		20		9				
**How likely overall would you be to keep it inserted in your vagina every day?**							1.0000	1.0000	1.0000
Unlikely/Very unlikely	2	(10.0)	2	(10.0)	0	(0.0)			
Likely/Very likely	18	(90.0)	18	(90.0)	9	(100)			
Total	20		20		9				

[1] P-values are from two-sided Mantel-Haenszel Chi-square exact test (ordinal variables) or Fisher's exact test (nominal variables).
[2] Solicited response categories.
[3] 'Would you like it if you could tell, just by looking at the vaginal ring, that while it was in your vagina, it had been releasing medicine to prevent HIV and/or pregnancy?'
[4] Please see the acceptability listing for details.

Table 14.4.4.1 Responses to Acceptability Questionnaire at IVR Removal, Completer Population by Treatment Group

	Treatment Group						Comparisons Between Treatment Group		
	TFV+LNG IVR (N= 20)		TFV Alone IVR (N= 20)		Placebo IVR (N= 10)		TFV+LNG vs TFV Alone	TFV+LNG vs Placebo	TFV Alone vs Placebo
	n	%	n	%	n	%	p-value[1]	p-value	p-value
**Have you ever used any of the following products for contraception?**									
Nuvaring	0	(0.0)	4	(20.0)	1	(11.1)	0.1060	0.3103	1.0000
Diaphragm	3	(15.0)	0	(0.0)	2	(22.2)	0.2308	0.6328	0.0887
Contraceptive Sponge	0	(0.0)	0	(0.0)	0	(0.0)			
Intrauterine Device (IUD)	3	(15.0)	1	(5.0)	2	(22.2)	0.6050	0.6328	0.2200
**Would Like to Know if Medicine Being Released[3]**							0.6050	0.5320	1.0000
No	1	(5.0)	3	(15.0)	1	(11.1)			
Yes	19	(95.0)	17	(85.0)	8	(88.9)			
Total	20		20		9				
**Do you have any suggestions for changes or improvements to the ring?[4]**							0.3416	0.2800	1.0000
No	16	(80.0)	19	(95.0)	9	(100)			
Yes	4	(20.0)	1	(5.0)	0	(0.0)			
Total	20		20		9				
**If a vaginal ring was available, what color do you think the vaginal ring should be?[4]**									
**Additional comments[4]**									

[1] P-values are from two-sided Mantel-Haenszel Chi-square exact test (ordinal variables) or Fisher's exact test (nominal variables).
[2] Solicited response categories.
[3] 'Would you like it if you could tell, just by looking at the vaginal ring, that while it was in your vagina, it had been releasing medicine to prevent HIV and/or pregnancy?'
[4] Please see the acceptability listing for details.
