# Supplementary material for: Randomized, placebo controlled phase I trial of safety, pharmacokinetics, pharmacodynamics and acceptability of tenofovir and tenofovir plus levonorgestrel vaginal rings in women
Source: PLoS One. 2018 Jun 28;13(6):e0199778. doi: 10.1371/journal.pone.0199778 (PMC6023238; doi:10.1371/journal.pone.0199778)
Supplement: S4 Data — (ZIP) [file pone.0199778.s009.zip › Accept Adhere Data/adhere.pdf]

Table 14.1.5 IVR Adherence and Exposure
Randomized Population

	Profamilia DR (853)				EVMS (908)				Overall			
	TFV+ LNG n (%)	TFV Alone n (%)	Placebo n (%)	Total n (%)	TFV+ LNG n (%)	TFV Alone n (%)	Placebo n (%)	Total n (%)	TFV+ LNG n (%)	TFV Alone n (%)	Placebo n (%)	Total n (%)
**Duration of Use (days)[1]**												
Mean (SD)	15.8 (3.59)	15.7 (1.97)	16.4 (2.80)	15.9 (2.80)	14.1 (1.77)	13.5 (1.95)	15.0 (4.02)	14.1 (2.39)	15.1 (2.97)	14.6 (2.21)	15.7 (3.34)	15.0 (2.75)
Median	15.0	15.0	17.0	15.0	14.0	13.0	14.1	14.0	14.0	14.0	15.1	14.0
Range	(12.0 to 23.0)	(13.9 to 19.1)	(13.0 to 19.0)	(12.0 to 23.0)	(12.0 to 18.0)	(11.0 to 17.0)	(10.0 to 21.1)	(10.0 to 21.1)	(12.0 to 23.0)	(11.0 to 19.1)	(10.0 to 21.1)	(10.0 to 23.0)
Total	11	10	5	26	9	10	5	24	20	20	10	50
**Adherence[2]**												
No	0 (0.0)	0 (0.0)	0 (0.0)	0 (0.0)	0 (0.0)	0 (0.0)	0 (0.0)	0 (0.0)	0 (0.0)	0 (0.0)	0 (0.0)	0 (0.0)
Yes	11 (100)	10 (100)	5 (100)	26 (100)	9 (100)	10 (100)	5 (100)	24 (100)	20 (100)	20 (100)	10 (100)	50 (100)
Total	11	10	5	26	9	10	5	24	20	20	10	50
**Number of removals or expulsions**												
1	11 (100)	11 (100)	5 (100)	27 (100)	8 (88.9)	10 (100)	5 (100)	23 (95.8)	19 (95.0)	21 (100)	10 (100)	50 (98.0)
2	0 (0.0)	0 (0.0)	0 (0.0)	0 (0.0)	1 (11.1)	0 (0.0)	0 (0.0)	1 (4.2)	1 (5.0)	0 (0.0)	0 (0.0)	1 (2.0)
Total	11	11	5	27	9	10	5	24	20	21	10	51
**How did ring come out?[3]**												
Removed	11 (100)	11 (100)	5 (100)	27 (100)	9 (90.0)	10 (100)	5 (100)	24 (96.0)	20 (95.2)	21 (100)	10 (100)	51 (98.1)
Expelled partially & removed	0 (0.0)	0 (0.0)	0 (0.0)	0 (0.0)	1 (10.0)	0 (0.0)	0 (0.0)	1 (4.0)	1 (4.8)	0 (0.0)	0 (0.0)	1 (1.9)
Expelled completely	0 (0.0)	0 (0.0)	0 (0.0)	0 (0.0)	0 (0.0)	0 (0.0)	0 (0.0)	0 (0.0)	0 (0.0)	0 (0.0)	0 (0.0)	0 (0.0)
Total	11	11	5	27	10	10	5	25	21	21	10	52

[1]Cumulative amount of time IVR in place
[2]Participant meets protocol definition for adherence (removed no more than twice before visit 7 with IVR out no more than 2 hours either time)
[3]Denominator is number of removals. The duration of the single expulsion was one minute.

Table 14.1.5 IVR Adherence and Exposure
Randomized Population

	Profamilia DR (853)								EVMS (908)								Overall							
	TFV+ LNG		TFV Alone		Placebo		Total		TFV+ LNG		TFV Alone		Placebo		Total		TFV+ LNG		TFV Alone		Placebo		Total	
	n	(%)	n	(%)	n	(%)	n	(%)	n	(%)	n	(%)	n	(%)	n	(%)	n	(%)	n	(%)	n	(%)	n	(%)
**Ring removed by whom?**[3]																								
Clinician	11	(100)	10	(90.9)	5	(100)	26	(96.3)	9	(90.0)	10	(100)	5	(100)	24	(96.0)	20	(95.2)	20	(95.2)	10	(100)	50	(96.2)
Participant	0	(0.0)	1	(9.1)	0	(0.0)	1	(3.7)	0	(0.0)	0	(0.0)	0	(0.0)	0	(0.0)	0	(0.0)	1	(4.8)	0	(0.0)	1	(1.9)
Other	0	(0.0)	0	(0.0)	0	(0.0)	0	(0.0)	1	(10.0)	0	(0.0)	0	(0.0)	1	(4.0)	1	(4.8)	0	(0.0)	0	(0.0)	1	(1.9)
Total	11		11		5		27		10		10		5		25		21		21		10		52	
**Reason for removal or expulsion**[3]																								
Scheduled removal	11	(100)	10	(90.9)	5	(100)	26	(96.3)	9	(90.0)	10	(100)	5	(100)	24	(96.0)	20	(95.2)	20	(95.2)	10	(100)	50	(96.2)
Discomfort/symptoms	0	(0.0)	0	(0.0)	0	(0.0)	0	(0.0)	0	(0.0)	0	(0.0)	0	(0.0)	0	(0.0)	0	(0.0)	0	(0.0)	0	(0.0)	0	(0.0)
Wanted to look or show someone, or check to see if the ring was still in place	0	(0.0)	0	(0.0)	0	(0.0)	0	(0.0)	0	(0.0)	0	(0.0)	0	(0.0)	0	(0.0)	0	(0.0)	0	(0.0)	0	(0.0)	0	(0.0)
Menses/Bleeding	0	(0.0)	0	(0.0)	0	(0.0)	0	(0.0)	0	(0.0)	0	(0.0)	0	(0.0)	0	(0.0)	0	(0.0)	0	(0.0)	0	(0.0)	0	(0.0)
Urination	0	(0.0)	0	(0.0)	0	(0.0)	0	(0.0)	0	(0.0)	0	(0.0)	0	(0.0)	0	(0.0)	0	(0.0)	0	(0.0)	0	(0.0)	0	(0.0)
Bowel movement	0	(0.0)	0	(0.0)	0	(0.0)	0	(0.0)	1	(10.0)	0	(0.0)	0	(0.0)	1	(4.0)	1	(4.8)	0	(0.0)	0	(0.0)	1	(1.9)
Before, during or after sex	0	(0.0)	0	(0.0)	0	(0.0)	0	(0.0)	0	(0.0)	0	(0.0)	0	(0.0)	0	(0.0)	0	(0.0)	0	(0.0)	0	(0.0)	0	(0.0)
Physical activity (other than sex) including squatting, sitting or changing body	0	(0.0)	0	(0.0)	0	(0.0)	0	(0.0)	0	(0.0)	0	(0.0)	0	(0.0)	0	(0.0)	0	(0.0)	0	(0.0)	0	(0.0)	0	(0.0)
Other	0	(0.0)	1	(9.1)	0	(0.0)	1	(3.7)	0	(0.0)	0	(0.0)	0	(0.0)	0	(0.0)	0	(0.0)	1	(4.8)	0	(0.0)	1	(1.9)
Total	11		11		5		27		10		10		5		25		21		21		10		52	

[1]Cumulative amount of time IVR in place
[2]Participant meets protocol definition for adherence (removed no more than twice before visit 7 with IVR out no more than 2 hours either time)
[3]Denominator is number of removals. The duration of the single expulsion was one minute.
