# Supplementary material for: Randomized, placebo controlled phase I trial of safety, pharmacokinetics, pharmacodynamics and acceptability of tenofovir and tenofovir plus levonorgestrel vaginal rings in women
Source: PLoS One. 2018 Jun 28;13(6):e0199778. doi: 10.1371/journal.pone.0199778 (PMC6023238; doi:10.1371/journal.pone.0199778)
Supplement: S5 Data — (ZIP) [file pone.0199778.s010.zip › Safety Data/CVL_SM47.pdf]

Table 14.3.6.1.1 Descriptive Statistics: Soluble Markers of Innate Mucosal Immunity and Inflammatory Response in Cervicovaginal Lavage (CVL) at Baseline and IVR Removal
Completer Population

	Treatment Group					
	TFV+LNG (N= 20)		TFV Alone (N= 21)		Placebo (N= 10)	
	Visit 4	Visit 7	Visit 4	Visit 7	Visit 4	Visit 7
**IL-1alpha (pg/ml)**						
Mean (SD)	445.5 (612.12)	1395.6 (4057.02)	326.2 (238.59)	875.7 (1125.93)	472.3 (728.74)	539.2 (807.87)
Median (Interquartile Range)	210.3 (130.7 to 495.1)	441.5 (258.4 to 648.5)	224.7 (162.5 to 438.2)	414.0 (174.5 to 1244.5)	178.1 (76.0 to 503.0)	188.1 (143.2 to 571.7)
Range (Min to Max)	(65.6 to 2565.7)	(70.4 to 18554.8)	(78.9 to 938.3)	(82.2 to 4574.5)	(74.8 to 2452.6)	(97.5 to 2725.7)
Total	20	20	20	20	10	10
**IL-6 (pg/ml)**						
Mean (SD)	78.7 (142.87)	40.2 (86.02)	115.6 (136.49)	29.7 (39.35)	68.4 (85.10)	26.2 (38.55)
Median (Interquartile Range)	25.3 (11.3 to 65.1)	7.6 (3.7 to 34.4)	57.9 (12.9 to 188.2)	17.8 (5.0 to 32.1)	14.7 (8.0 to 149.3)	9.5 (4.2 to 25.0)
Range (Min to Max)	(8.2 to 630.0)	(0.4 to 378.5)	(2.0 to 457.0)	(0.2 to 157.5)	(2.2 to 236.7)	(0.5 to 121.4)
Total	20	20	20	20	10	9
**IP10 (pg/ml)**						
Mean (SD)	297.1 (447.52)	442.4 (634.71)	190.1 (144.40)	107.1 (103.81)	171.3 (154.30)	122.0 (139.50)
Median (Interquartile Range)	86.4 (29.8 to 417.5)	155.4 (50.7 to 544.7)	172.0 (75.4 to 280.8)	82.4 (21.1 to 146.3)	122.2 (44.3 to 317.7)	74.7 (19.4 to 167.2)
Range (Min to Max)	(7.4 to 1483.3)	(1.1 to 2156.2)	(2.8 to 491.0)	(2.8 to 404.9)	(3.1 to 466.4)	(3.9 to 444.9)
Total	20	20	20	20	10	10
**MIP-1alpha (pg/ml)**						
Mean (SD)	47.5 (24.04)	51.5 (33.99)	121.4 (255.47)	20.1 (4.51)	24.2 (10.51)	20.2 (2.62)
Median (Interquartile Range)	40.0 (31.6 to 58.7)	41.2 (24.8 to 87.2)	39.6 (23.4 to 83.4)	17.8 (17.3 to 24.4)	20.5 (17.6 to 27.2)	20.8 (17.3 to 22.5)
Range (Min to Max)	(21.4 to 94.7)	(18.3 to 96.6)	(16.9 to 961.8)	(15.9 to 30.0)	(15.3 to 46.1)	(17.3 to 22.5)
Total	11	6	13	11	7	3

Table 14.3.6.1.1 Descriptive Statistics: Soluble Markers of Innate Mucosal Immunity and Inflammatory Response in Cervicovaginal Lavage (CVL) at Baseline and IVR Removal
Completer Population

	Treatment Group					
	TFV+LNG (N= 20)		TFV Alone (N= 21)		Placebo (N= 10)	
	Visit 4	Visit 7	Visit 4	Visit 7	Visit 4	Visit 7
**RANTES (pg/ml)**						
Mean (SD)	36.8 (118.81)	7.3 (8.81)	41.2 (121.92)	57.1 (180.68)	183.3 (556.83)	6.6 (9.87)
Median (Interquartile Range)	6.1 (3.3 to 17.9)	4.2 (1.4 to 9.6)	7.5 (3.7 to 17.8)	2.2 (1.6 to 5.7)	8.2 (2.6 to 11.9)	3.4 (0.8 to 6.2)
Range (Min to Max)	(0.6 to 539.7)	(0.1 to 36.7)	(0.6 to 553.0)	(0.2 to 756.2)	(1.7 to 1768.0)	(0.2 to 31.9)
Total	20	20	20	20	10	10
**TNF alpha (pg/ml)**						
Mean (SD)	5.5 (11.06)	3.2 (5.99)	22.9 (51.73)	1.3 (1.62)	1.5 (1.90)	0.9 (0.33)
Median (Interquartile Range)	2.3 (0.9 to 4.6)	1.0 (0.6 to 1.9)	2.2 (0.7 to 8.6)	0.7 (0.5 to 0.9)	0.9 (0.8 to 1.3)	0.9 (0.5 to 1.2)
Range (Min to Max)	(0.4 to 49.8)	(0.5 to 21.1)	(0.5 to 198.8)	(0.4 to 7.2)	(0.4 to 6.5)	(0.5 to 1.3)
Total	19	19	19	18	9	9
**GM-CSF (pg/ml)**						
Mean (SD)	1.3 (0.81)	1.6 (1.71)	4.3 (11.52)	1.3 (0.85)	1.9 (2.32)	2.5 (3.27)
Median (Interquartile Range)	1.2 (0.6 to 1.7)	0.9 (0.6 to 1.5)	1.0 (0.5 to 2.6)	0.8 (0.6 to 1.8)	0.9 (0.7 to 2.0)	1.0 (0.8 to 4.2)
Range (Min to Max)	(0.5 to 3.3)	(0.5 to 6.8)	(0.4 to 51.3)	(0.4 to 3.2)	(0.6 to 7.4)	(0.6 to 7.4)
Total	18	16	19	17	8	4
**IL-10 (pg/ml)**						
Mean (SD)	6.4 (14.99)	3.9 (10.21)	113.3 (460.93)	1.0 (1.07)	0.7 (0.26)	0.5 (0.27)
Median (Interquartile Range)	0.9 (0.5 to 2.0)	1.0 (0.4 to 2.4)	1.4 (0.6 to 4.5)	0.7 (0.4 to 1.0)	0.7 (0.6 to 0.8)	0.5 (0.3 to 0.5)
Range (Min to Max)	(0.2 to 60.8)	(0.2 to 41.9)	(0.2 to 1959.7)	(0.3 to 3.7)	(0.4 to 1.2)	(0.2 to 1.1)
Total	17	16	18	15	8	9

Table 14.3.6.1.1 Descriptive Statistics: Soluble Markers of Innate Mucosal Immunity and Inflammatory Response in Cervicovaginal Lavage (CVL) at Baseline and IVR Removal
Completer Population

	Treatment Group					
	TFV+LNG (N= 20)		TFV Alone (N= 21)		Placebo (N= 10)	
	Visit 4	Visit 7	Visit 4	Visit 7	Visit 4	Visit 7
**IL-8 (pg/ml)**						
Mean (SD)	2368.3 (2859.00)	2130.3 (2352.38)	4646.2 (6435.76)	3076.2 (4350.72)	2766.8 (2488.03)	1352.9 (1131.29)
Median (Interquartile Range)	1433.2 (515.4 to 2975.5)	1066.2 (393.5 to 3493.1)	3298.6 (1024.0 to 4912.1)	1621.7 (865.7 to 3412.2)	1809.0 (989.7 to 4198.4)	1267.9 (82.2 to 2406.4)
Range (Min to Max)	(204.9 to 12793.6)	(2.4 to 9591.0)	(81.0 to 29333.3)	(142.8 to 19518.1)	(165.4 to 7646.4)	(1.7 to 2909.2)
Total	20	20	20	20	10	10
**BD2 (pg/ml)**						
Mean (SD)	135231.7 (237183.79)	298742.1 (640883.97)	227606.0 (319950.13)	587265.4 (1196729.28)	276205.8 (432394.28)	372245.6 (476048.15)
Median (Interquartile Range)	37773.6 (13120.8 to 95979.3)	121403.1 (34639.7 to 214919.4)	46903.4 (7000.9 to 323610.9)	119891.2 (31456.1 to 446732.0)	45073.8 (26644.4 to 354476.8)	124223.6 (93647.4 to 656466.4)
Range (Min to Max)	(4618.8 to 898592.1)	(0.0 to 2870986.7)	(617.7 to 1121144.3)	(965.7 to 5106960.5)	(2786.9 to 1288965.0)	(3016.7 to 1269567.5)
Total	20	20	20	20	10	10
**SLPI (pg/ml)**						
Mean (SD)	382271.4 (451557.85)	741857.7 (1049073.35)	519760.1 (942373.93)	370093.9 (495446.57)	524849.7 (462116.43)	551287.7 (808562.98)
Median (Interquartile Range)	204796.9 (99070.3 to 532023.0)	575986.3 (83883.3 to 983453.1)	143263.3 (65609.8 to 680597.1)	165659.9 (47983.7 to 519923.1)	434475.2 (184987.3 to 590171.7)	196406.4 (119658.2 to 418237.9)
Range (Min to Max)	(7658.2 to 1932007.6)	(7387.6 to 4680625.7)	(27522.8 to 4274380.6)	(7813.4 to 1810518.5)	(21127.4 to 1455217.5)	(7826.4 to 2135437.1)
Total	20	20	20	20	10	10

Table 14.3.6.1.1 Descriptive Statistics: Soluble Markers of Innate Mucosal Immunity and Inflammatory Response in Cervicovaginal Lavage (CVL) at Baseline and IVR Removal
Completer Population

	Treatment Group					
	TFV+LNG (N= 20)		TFV Alone (N= 21)		Placebo (N= 10)	
	Visit 4	Visit 7	Visit 4	Visit 7	Visit 4	Visit 7
**IL-1RA (pg/ml)**						
Mean (SD)	298633.5 (330956.96)	319099.3 (341698.19)	333529.2 (333707.11)	296959.2 (356705.66)	426513.3 (345376.42)	247274.7 (261555.05)
Median (Interquartile Range)	155254.0 (79187.1 to 334007.0)	141048.2 (71752.2 to 516054.0)	194881.9 (72773.8 to 592592.4)	125473.1 (80182.3 to 330365.7)	347760.8 (163824.8 to 632852.3)	175149.2 (24721.3 to 286789.9)
Range (Min to Max)	(50237.4 to 1074583.8)	(19912.0 to 1079336.4)	(40217.0 to 1091227.8)	(22621.3 to 1319059.0)	(75833.3 to 1100838.4)	(18459.2 to 752622.6)
Total	20	20	20	20	10	10
