# Supplementary material for: Randomized, placebo controlled phase I trial of safety, pharmacokinetics, pharmacodynamics and acceptability of tenofovir and tenofovir plus levonorgestrel vaginal rings in women
Source: PLoS One. 2018 Jun 28;13(6):e0199778. doi: 10.1371/journal.pone.0199778 (PMC6023238; doi:10.1371/journal.pone.0199778)
Supplement: S5 Data — (ZIP) [file pone.0199778.s010.zip › Safety Data/FLORA_SQC47.pdf]

Table 14.3.6.3.3. Descriptive Statistics: Nugent Score and Microflora (Semi-Quantitative Culture) at Baseline and IVR Removal
Completer Population

	Treatment Group					
	TFV+LNG (N= 20)		TFV Alone (N= 20)		Placebo (N= 10)	
	Visit 4	Visit 7	Visit 4	Visit 7	Visit 4	Visit 7
**NUGENT SCORE**						
0	3 (20.0)	7 (46.7)	3 (17.6)	6 (37.5)	3 (33.3)	3 (33.3)
1	4 (26.7)	2 (13.3)	2 (11.8)	1 (6.3)	0 (0.0)	2 (22.2)
2	3 (20.0)	2 (13.3)	2 (11.8)	2 (12.5)	2 (22.2)	0 (0.0)
3	2 (13.3)	1 (6.7)	3 (17.6)	0 (0.0)	1 (11.1)	0 (0.0)
4	1 (6.7)	2 (13.3)	3 (17.6)	3 (18.8)	1 (11.1)	1 (11.1)
5	1 (6.7)	0 (0.0)	1 (5.9)	0 (0.0)	0 (0.0)	1 (11.1)
6	0 (0.0)	0 (0.0)	1 (5.9)	1 (6.3)	0 (0.0)	0 (0.0)
7	0 (0.0)	0 (0.0)	0 (0.0)	2 (12.5)	0 (0.0)	0 (0.0)
8	0 (0.0)	0 (0.0)	2 (11.8)	1 (6.3)	2 (22.2)	2 (22.2)
9	1 (6.7)	1 (6.7)	0 (0.0)	0 (0.0)	0 (0.0)	0 (0.0)
Total	15	15	17	16	9	9
**NUGENT SCORE (categorical)**						
Normal (0-3)	12 (80.0)	12 (80.0)	10 (58.8)	9 (56.3)	6 (66.7)	5 (55.6)
Intermediate (4-6)	2 (13.3)	2 (13.3)	5 (29.4)	4 (25.0)	1 (11.1)	2 (22.2)
High (7-10)	1 (6.7)	1 (6.7)	2 (11.8)	3 (18.8)	2 (22.2)	2 (22.2)
Total	15	15	17	16	9	9

Table 14.3.6.3.3. Descriptive Statistics: Nugent Score and Microflora (Semi-Quantitative Culture) at Baseline and IVR Removal
Completer Population

	Treatment Group					
	TFV+LNG (N= 20)		TFV Alone (N= 20)		Placebo (N= 10)	
	Visit 4	Visit 7	Visit 4	Visit 7	Visit 4	Visit 7
**ANAEROBIC GRAM-NEGATIVE BACILLUS**						
0 (NONE SEEN)	3 (18.8)	6 (37.5)	4 (21.1)	6 (35.3)	3 (33.3)	3 (33.3)
1+ (RARE)	5 (31.3)	2 (12.5)	2 (10.5)	1 (5.9)	0 (0.0)	2 (22.2)
2+ (FEW)	3 (18.8)	3 (18.8)	3 (15.8)	2 (11.8)	2 (22.2)	0 (0.0)
3+ (MODERATE)	1 (6.3)	1 (6.3)	2 (10.5)	1 (5.9)	1 (11.1)	0 (0.0)
4+ (MANY)	4 (25.0)	4 (25.0)	8 (42.1)	7 (41.2)	3 (33.3)	4 (44.4)
Total	16	16	19	17	9	9
**CANDIDA**						
0 (NONE SEEN)	14 (87.5)	15 (93.8)	19 (100)	14 (82.4)	7 (87.5)	7 (77.8)
1+ (RARE)	1 (6.3)	0 (0.0)	0 (0.0)	1 (5.9)	0 (0.0)	1 (11.1)
2+ (FEW)	0 (0.0)	0 (0.0)	0 (0.0)	1 (5.9)	0 (0.0)	0 (0.0)
3+ (MODERATE)	1 (6.3)	1 (6.3)	0 (0.0)	1 (5.9)	1 (12.5)	1 (11.1)
Total	16	16	19	17	8	9
**ESCHERICHIA COLI**						
0 (NONE SEEN)	15 (93.8)	16 (100)	16 (84.2)	15 (88.2)	6 (75.0)	7 (77.8)
2+ (FEW)	1 (6.3)	0 (0.0)	1 (5.3)	0 (0.0)	1 (12.5)	1 (11.1)
3+ (MODERATE)	0 (0.0)	0 (0.0)	1 (5.3)	2 (11.8)	1 (12.5)	1 (11.1)
4+ (MANY)	0 (0.0)	0 (0.0)	1 (5.3)	0 (0.0)	0 (0.0)	0 (0.0)
Total	16	16	19	17	8	9

Table 14.3.6.3.3. Descriptive Statistics: Nugent Score and Microflora (Semi-Quantitative Culture) at Baseline and IVR Removal
Completer Population

	Treatment Group					
	TFV+LNG (N= 20)		TFV Alone (N= 20)		Placebo (N= 10)	
	Visit 4	Visit 7	Visit 4	Visit 7	Visit 4	Visit 7
**ENTEROCOCCUS**						
0 (NONE SEEN)	12 (75.0)	12 (75.0)	14 (73.7)	11 (64.7)	6 (75.0)	6 (66.7)
1+ (RARE)	1 (6.3)	0 (0.0)	2 (10.5)	2 (11.8)	0 (0.0)	0 (0.0)
2+ (FEW)	2 (12.5)	1 (6.3)	2 (10.5)	1 (5.9)	1 (12.5)	1 (11.1)
3+ (MODERATE)	1 (6.3)	3 (18.8)	1 (5.3)	2 (11.8)	0 (0.0)	1 (11.1)
4+ (MANY)	0 (0.0)	0 (0.0)	0 (0.0)	1 (5.9)	1 (12.5)	1 (11.1)
Total	16	16	19	17	8	9
**GARDNERELLA VAGINALIS**						
0 (NONE SEEN)	11 (68.8)	10 (62.5)	10 (52.6)	10 (58.8)	4 (50.0)	4 (44.4)
1+ (RARE)	1 (6.3)	0 (0.0)	0 (0.0)	0 (0.0)	0 (0.0)	2 (22.2)
2+ (FEW)	1 (6.3)	0 (0.0)	1 (5.3)	3 (17.6)	2 (25.0)	0 (0.0)
3+ (MODERATE)	3 (18.8)	5 (31.3)	5 (26.3)	3 (17.6)	1 (12.5)	2 (22.2)
4+ (MANY)	0 (0.0)	1 (6.3)	3 (15.8)	1 (5.9)	1 (12.5)	1 (11.1)
Total	16	16	19	17	8	9
**STREPTOCOCCUS AGALACTIAE**						
0 (NONE SEEN)	15 (93.8)	15 (93.8)	17 (89.5)	16 (94.1)	4 (50.0)	6 (66.7)
2+ (FEW)	0 (0.0)	0 (0.0)	2 (10.5)	1 (5.9)	1 (12.5)	1 (11.1)
3+ (MODERATE)	0 (0.0)	1 (6.3)	0 (0.0)	0 (0.0)	2 (25.0)	2 (22.2)
4+ (MANY)	1 (6.3)	0 (0.0)	0 (0.0)	0 (0.0)	1 (12.5)	0 (0.0)
Total	16	16	19	17	8	9

Table 14.3.6.3.3. Descriptive Statistics: Nugent Score and Microflora (Semi-Quantitative Culture) at Baseline and IVR Removal
Completer Population

	Treatment Group					
	TFV+LNG (N= 20)		TFV Alone (N= 20)		Placebo (N= 10)	
	Visit 4	Visit 7	Visit 4	Visit 7	Visit 4	Visit 7
**LACTOBACILLUS H2O2 -**						
0 (NONE SEEN)	10 (62.5)	11 (68.8)	15 (78.9)	13 (76.5)	6 (75.0)	6 (66.7)
1+ (RARE)	1 (6.3)	1 (6.3)	0 (0.0)	1 (5.9)	0 (0.0)	0 (0.0)
2+ (FEW)	0 (0.0)	0 (0.0)	2 (10.5)	1 (5.9)	0 (0.0)	2 (22.2)
3+ (MODERATE)	3 (18.8)	1 (6.3)	2 (10.5)	0 (0.0)	0 (0.0)	0 (0.0)
4+ (MANY)	2 (12.5)	3 (18.8)	0 (0.0)	2 (11.8)	2 (25.0)	1 (11.1)
Total	16	16	19	17	8	9
**LACTOBACILLUS H2O2 +**						
0 (NONE SEEN)	3 (18.8)	3 (18.8)	6 (31.6)	4 (23.5)	1 (12.5)	0 (0.0)
2+ (FEW)	5 (31.3)	1 (6.3)	1 (5.3)	4 (23.5)	1 (12.5)	0 (0.0)
3+ (MODERATE)	5 (31.3)	5 (31.3)	5 (26.3)	6 (35.3)	2 (25.0)	2 (22.2)
4+ (MANY)	3 (18.8)	7 (43.8)	7 (36.8)	3 (17.6)	4 (50.0)	7 (77.8)
Total	16	16	19	17	8	9
**MYCOPLASMA**						
0 (NONE SEEN)	13 (81.3)	13 (81.3)	17 (89.5)	14 (82.4)	8 (100)	8 (100)
1+ (RARE)	2 (12.5)	2 (12.5)	0 (0.0)	1 (5.9)	0 (0.0)	0 (0.0)
2+ (FEW)	1 (6.3)	0 (0.0)	1 (5.3)	0 (0.0)	0 (0.0)	0 (0.0)
3+ (MODERATE)	0 (0.0)	0 (0.0)	1 (5.3)	1 (5.9)	0 (0.0)	0 (0.0)
4+ (MANY)	0 (0.0)	1 (6.3)	0 (0.0)	1 (5.9)	0 (0.0)	0 (0.0)
Total	16	16	19	17	8	8

Table 14.3.6.3.3. Descriptive Statistics: Nugent Score and Microflora (Semi-Quantitative Culture) at Baseline and IVR Removal
Completer Population

	Treatment Group					
	TFV+LNG (N= 20)		TFV Alone (N= 20)		Placebo (N= 10)	
	Visit 4	Visit 7	Visit 4	Visit 7	Visit 4	Visit 7
**PIGMENTED ANAEROBIC GRAM NEGATIVE RODS**						
0 (NONE SEEN)	13 (81.3)	11 (68.8)	15 (78.9)	14 (82.4)	7 (87.5)	7 (77.8)
1+ (RARE)	1 (6.3)	3 (18.8)	1 (5.3)	0 (0.0)	0 (0.0)	1 (11.1)
2+ (FEW)	0 (0.0)	0 (0.0)	1 (5.3)	1 (5.9)	1 (12.5)	0 (0.0)
3+ (MODERATE)	1 (6.3)	1 (6.3)	1 (5.3)	1 (5.9)	0 (0.0)	1 (11.1)
4+ (MANY)	1 (6.3)	1 (6.3)	1 (5.3)	1 (5.9)	0 (0.0)	0 (0.0)
Total	16	16	19	17	8	9
**STAPHYLOCOCCUS AUREUS**						
0 (NONE SEEN)	16 (100)	14 (87.5)	16 (84.2)	16 (94.1)	6 (75.0)	8 (88.9)
1+ (RARE)	0 (0.0)	1 (6.3)	1 (5.3)	0 (0.0)	0 (0.0)	0 (0.0)
2+ (FEW)	0 (0.0)	0 (0.0)	0 (0.0)	0 (0.0)	1 (12.5)	1 (11.1)
3+ (MODERATE)	0 (0.0)	0 (0.0)	1 (5.3)	1 (5.9)	1 (12.5)	0 (0.0)
4+ (MANY)	0 (0.0)	1 (6.3)	1 (5.3)	0 (0.0)	0 (0.0)	0 (0.0)
Total	16	16	19	17	8	9
**UREAPLASMA**						
0 (NONE SEEN)	11 (68.8)	12 (75.0)	10 (52.6)	10 (58.8)	5 (62.5)	7 (87.5)
1+ (RARE)	1 (6.3)	2 (12.5)	3 (15.8)	3 (17.6)	0 (0.0)	0 (0.0)
2+ (FEW)	4 (25.0)	1 (6.3)	5 (26.3)	3 (17.6)	2 (25.0)	1 (12.5)
3+ (MODERATE)	0 (0.0)	1 (6.3)	1 (5.3)	1 (5.9)	0 (0.0)	0 (0.0)
4+ (MANY)	0 (0.0)	0 (0.0)	0 (0.0)	0 (0.0)	1 (12.5)	0 (0.0)
Total	16	16	19	17	8	8
