# Supplementary material for: Randomized, placebo controlled phase I trial of safety, pharmacokinetics, pharmacodynamics and acceptability of tenofovir and tenofovir plus levonorgestrel vaginal rings in women
Source: PLoS One. 2018 Jun 28;13(6):e0199778. doi: 10.1371/journal.pone.0199778 (PMC6023238; doi:10.1371/journal.pone.0199778)
Supplement: S5 Data — (ZIP) [file pone.0199778.s010.zip › Safety Data/IMMUNE_CELL_ACTIVATION37.pdf]

Table 14.3.6.2.1 Descriptive Statistics: HIV-1 Target Immune Cell Phenotype and Activation/Proliferation Markers in Tissue at Baseline and IVR Removal
Completer Population

	Treatment Group					
	TFV+LNG (N= 20)		TFV Alone (N= 20)		Placebo (N= 10)	
	Visit 3	Visit 7	Visit 3	Visit 7	Visit 3	Visit 7
**Epithelial thickness (um)**						
Mean (SD)	228.5 (91.80)	224.9 (94.24)	238.3 (91.09)	203.0 (96.75)	227.7 (72.84)	217.5 (72.95)
Median (Interquartile Range)	209.2 (166.7 to 254.0)	216.2 (143.3 to 265.0)	218.2 (167.5 to 265.9)	202.0 (150.0 to 245.0)	215.9 (195.0 to 285.0)	228.0 (153.3 to 252.0)
Range (Min to Max)	(96.0 to 442.0)	(121.7 to 458.0)	(148.3 to 461.7)	(24.0 to 450.0)	(95.0 to 363.3)	(115.0 to 335.0)
Total	18	18	20	20	10	10
**Epithelial thickness (cell layers)**						
Mean (SD)	20.7 (5.73)	22.2 (5.09)	20.5 (5.52)	21.0 (5.63)	21.2 (5.91)	20.1 (5.12)
Median (Interquartile Range)	20.0 (17.6 to 24.3)	21.3 (18.4 to 26.5)	19.9 (16.6 to 22.3)	21.4 (18.4 to 23.4)	22.2 (17.7 to 24.0)	19.6 (15.7 to 24.2)
Range (Min to Max)	(9.4 to 34.2)	(12.0 to 32.4)	(13.4 to 33.4)	(8.2 to 36.2)	(9.8 to 30.2)	(11.8 to 29.0)
Total	18	18	20	20	10	10
**CD45 Epithelium (cells/sqmm)**						
Mean (SD)	108.7 (43.29)	138.4 (72.62)	105.9 (43.43)	115.3 (73.91)	96.2 (38.49)	129.1 (51.04)
Median (Interquartile Range)	103.1 (85.4 to 117.4)	145.1 (74.4 to 165.5)	103.8 (72.2 to 126.8)	94.8 (78.7 to 113.3)	100.9 (75.6 to 106.0)	132.4 (74.2 to 149.9)
Range (Min to Max)	(37.6 to 207.3)	(49.7 to 312.7)	(38.7 to 193.9)	(50.7 to 385.8)	(38.1 to 178.8)	(60.1 to 215.3)
Total	18	18	20	20	10	10
**CD 45 Lamina Propria (cells/HPF)**						
Mean (SD)	78.2 (39.87)	125.3 (96.32)	77.0 (28.89)	104.7 (49.01)	95.4 (33.86)	115.8 (45.80)
Median (Interquartile Range)	70.7 (48.0 to 96.0)	89.4 (67.2 to 140.0)	76.8 (56.0 to 85.3)	88.0 (65.3 to 162.7)	95.0 (64.0 to 122.7)	104.0 (82.7 to 150.4)
Range (Min to Max)	(38.4 to 201.6)	(24.0 to 410.0)	(35.2 to 166.4)	(32.0 to 179.2)	(52.0 to 147.2)	(64.0 to 198.4)
Total	18	18	19	19	10	10

[1] >25% of values are 0. Per SAP the variable represented as dichotomy '0' vs '>0'.

Table 14.3.6.2.1 Descriptive Statistics: HIV-1 Target Immune Cell Phenotype and Activation/Proliferation Markers in Tissue at Baseline and IVR Removal
Completer Population

	Treatment Group					
	TFV+LNG (N= 20)		TFV Alone (N= 20)		Placebo (N= 10)	
	Visit 3	Visit 7	Visit 3	Visit 7	Visit 3	Visit 7
**CD3 Epithelium (cells/sqmm)**						
Mean (SD)	83.2 (37.43)	97.5 (54.97)	80.7 (36.20)	87.7 (62.12)	68.9 (30.82)	99.4 (44.87)
Median (Interquartile Range)	78.7 (66.7 to 92.5)	93.8 (63.3 to 120.5)	80.5 (50.8 to 101.6)	67.1 (56.6 to 92.6)	61.1 (50.4 to 82.1)	102.1 (56.1 to 142.2)
Range (Min to Max)	(29.9 to 174.5)	(33.6 to 243.6)	(26.9 to 147.7)	(43.2 to 317.1)	(33.8 to 145.5)	(33.4 to 160.8)
Total	18	18	20	20	10	10
**CD3 Lamina Propria (cells/HPF)**						
Mean (SD)	58.1 (35.05)	92.0 (75.52)	53.1 (24.43)	73.4 (38.97)	68.0 (30.67)	74.1 (31.70)
Median (Interquartile Range)	49.9 (34.7 to 66.7)	64.0 (42.7 to 120.0)	44.0 (36.0 to 64.0)	64.0 (40.0 to 109.3)	63.0 (40.0 to 90.7)	65.3 (48.0 to 92.8)
Range (Min to Max)	(16.0 to 158.7)	(16.0 to 284.0)	(19.2 to 124.8)	(26.7 to 138.8)	(28.0 to 121.6)	(38.4 to 136.0)
Total	18	18	19	19	10	10
**HLADR Epithelium (cells/sqmm)**						
Mean (SD)	46.9 (15.40)	58.4 (34.23)	52.0 (30.84)	62.9 (47.67)	43.9 (19.22)	62.3 (28.68)
Median (Interquartile Range)	48.7 (31.3 to 60.6)	49.0 (36.7 to 71.0)	45.3 (30.3 to 61.8)	47.0 (39.7 to 72.5)	43.2 (31.9 to 57.4)	66.4 (34.3 to 78.6)
Range (Min to Max)	(25.0 to 75.1)	(31.7 to 145.5)	(17.7 to 113.3)	(19.5 to 243.1)	(16.7 to 81.8)	(21.5 to 102.1)
Total	18	18	20	20	10	10
**HLADR Lamina Propria (cells/HPF)**						
Mean (SD)	36.9 (8.27)	51.3 (26.49)	37.1 (15.95)	54.1 (19.85)	30.3 (8.88)	46.1 (24.47)
Median (Interquartile Range)	34.7 (32.0 to 41.6)	45.4 (37.3 to 60.0)	34.7 (25.6 to 44.0)	56.0 (40.0 to 73.6)	32.0 (25.6 to 32.0)	40.8 (29.3 to 57.6)
Range (Min to Max)	(24.0 to 52.0)	(16.0 to 128.0)	(21.3 to 92.0)	(16.0 to 92.8)	(16.0 to 48.0)	(18.7 to 104.6)
Total	18	18	19	19	10	10

[1] >25% of values are 0. Per SAP the variable represented as dichotomy '0' vs '>0'.

Table 14.3.6.2.1 Descriptive Statistics: HIV-1 Target Immune Cell Phenotype and Activation/Proliferation Markers in Tissue at Baseline and IVR Removal
Completer Population

	Treatment Group					
	TFV+LNG (N= 20)		TFV Alone (N= 20)		Placebo (N= 10)	
	Visit 3	Visit 7	Visit 3	Visit 7	Visit 3	Visit 7
**CCR5 in epithelium (cells/sqmm)[1]**						
Zero	18 (100)	18 (100)	20 (100)	19 (95.0)	10 (100)	10 (100)
Greater than zero	0 (0.0)	0 (0.0)	0 (0.0)	1 (5.0)	0 (0.0)	0 (0.0)
Total	18	18	20	20	10	10
**CCR5 Lamina Propria (cells/HPF)[1]**						
Zero	12 (66.7)	9 (50.0)	12 (63.2)	8 (42.1)	8 (80.0)	3 (30.0)
Greater than zero	6 (33.3)	9 (50.0)	7 (36.8)	11 (57.9)	2 (20.0)	7 (70.0)
Total	18	18	19	19	10	10
**CD4 in epithelium (cells/sqmm)[1]**						
Zero	13 (72.2)	12 (66.7)	14 (70.0)	16 (80.0)	9 (90.0)	10 (100)
Greater than zero	5 (27.8)	6 (33.3)	6 (30.0)	4 (20.0)	1 (10.0)	0 (0.0)
Total	18	18	20	20	10	10
**CD4 Lamina Propria (cells/HPF)[1]**						
Zero	8 (44.4)	4 (22.2)	5 (26.3)	5 (26.3)	1 (10.0)	4 (40.0)
Greater than zero	10 (55.6)	14 (77.8)	14 (73.7)	14 (73.7)	9 (90.0)	6 (60.0)
Total	18	18	19	19	10	10

[1] >25% of values are 0. Per SAP the variable represented as dichotomy '0' vs '>0'.

Table 14.3.6.2.1 Descriptive Statistics: HIV-1 Target Immune Cell Phenotype and Activation/Proliferation Markers in Tissue at Baseline and IVR Removal
Completer Population

	Treatment Group					
	TFV+LNG (N= 20)		TFV Alone (N= 20)		Placebo (N= 10)	
	Visit 3	Visit 7	Visit 3	Visit 7	Visit 3	Visit 7
**CD8 in epithelium (cells/sqmm)**						
Mean (SD)	58.4 (29.57)	71.6 (43.80)	53.1 (28.03)	60.2 (52.25)	43.4 (18.97)	64.4 (27.53)
Median (Interquartile Range)	48.6 (42.9 to 68.2)	60.1 (39.6 to 100.2)	46.1 (36.3 to 74.2)	43.8 (33.3 to 70.8)	43.6 (23.9 to 61.5)	59.8 (44.3 to 77.0)
Range (Min to Max)	(23.1 to 131.8)	(20.2 to 189.1)	(14.4 to 119.0)	(23.9 to 253.7)	(19.1 to 72.0)	(32.8 to 124.5)
Total	18	18	20	20	10	10
**CD8 Lamina Propria (cells/HPF)**						
Mean (SD)	36.1 (26.16)	58.7 (49.11)	34.6 (18.72)	48.4 (28.10)	38.4 (15.36)	49.4 (26.55)
Median (Interquartile Range)	30.4 (16.0 to 42.7)	38.7 (29.3 to 69.3)	32.0 (22.4 to 38.4)	37.3 (24.0 to 72.0)	36.3 (24.0 to 52.0)	40.0 (29.3 to 60.8)
Range (Min to Max)	(0.0 to 104.0)	(8.0 to 198.0)	(9.6 to 96.0)	(13.3 to 97.6)	(16.0 to 58.7)	(21.3 to 98.7)
Total	18	18	19	19	10	10

[1] >25% of values are 0. Per SAP the variable represented as dichotomy '0' vs '>0'.
