# Supplementary material for: Randomized, placebo controlled phase I trial of safety, pharmacokinetics, pharmacodynamics and acceptability of tenofovir and tenofovir plus levonorgestrel vaginal rings in women
Source: PLoS One. 2018 Jun 28;13(6):e0199778. doi: 10.1371/journal.pone.0199778 (PMC6023238; doi:10.1371/journal.pone.0199778)
Supplement: S5 Data — (ZIP) [file pone.0199778.s010.zip › Safety Data/SWAB_EVMS7.pdf]

Table 14.3.6.4.1 Exploratory Endpoint (Exp 3): Microbial Levels in Returned IVRs and Microbial Growth on Swabs from Returned IVRs (EVMS Only)
Completer Population

	Treatment Group		
	**TFV+LNG (N= 9)**	**TFV Alone (N= 10)**	**Placebo (N= 5)**
**ANAEROBIC GRAM-NEGATIVE BACILLUS**			
0 (NONE SEEN)	9 (100)	10 (100)	4 (100)
Total	9	10	4
**CANDIDA**			
0 (NONE SEEN)	9 (100)	10 (100)	4 (100)
Total	9	10	4
**ESCHERICHIA COLI**			
0 (NONE SEEN)	9 (100)	10 (100)	4 (100)
Total	9	10	4
**ENTEROCOCCUS**			
0 (NONE SEEN)	8 (88.9)	9 (90.0)	4 (100)
1+ (RARE)	1 (11.1)	0 (0.0)	0 (0.0)
2+ (FEW)	0 (0.0)	1 (10.0)	0 (0.0)
Total	9	10	4
**GARDNERELLA VAGINALIS**			
0 (NONE SEEN)	9 (100)	10 (100)	3 (75.0)
1+ (RARE)	0 (0.0)	0 (0.0)	1 (25.0)
Total	9	10	4
**STREPTOCOCCUS AGALACTIAE**			
0 (NONE SEEN)	9 (100)	10 (100)	4 (100)
Total	9	10	4

Table 14.3.6.4.1 Exploratory Endpoint (Exp 3): Microbial Levels in Returned IVRs and Microbial Growth on Swabs from Returned IVRs (EVMS Only)
Completer Population

	Treatment Group		
	**TFV+LNG (N= 9)**	**TFV Alone (N= 10)**	**Placebo (N= 5)**
**LACTOBACILLUS H2O2 -**			
0 (NONE SEEN)	9 (100)	7 (70.0)	4 (100)
1+ (RARE)	0 (0.0)	2 (20.0)	0 (0.0)
3+ (MODERATE)	0 (0.0)	1 (10.0)	0 (0.0)
Total	9	10	4
**LACTOBACILLUS H2O2 +**			
0 (NONE SEEN)	2 (22.2)	5 (50.0)	3 (75.0)
1+ (RARE)	4 (44.4)	4 (40.0)	1 (25.0)
2+ (FEW)	2 (22.2)	0 (0.0)	0 (0.0)
3+ (MODERATE)	1 (11.1)	0 (0.0)	0 (0.0)
4+ (MANY)	0 (0.0)	1 (10.0)	0 (0.0)
Total	9	10	4
**MYCOPLASMA**			
0 (NONE SEEN)	9 (100)	9 (90.0)	4 (100)
1+ (RARE)	0 (0.0)	1 (10.0)	0 (0.0)
Total	9	10	4
**PIGMENTED ANAEROBIC GRAM NEGATIVE RODS**			
0 (NONE SEEN)	9 (100)	10 (100)	3 (75.0)
1+ (RARE)	0 (0.0)	0 (0.0)	1 (25.0)
Total	9	10	4

Table 14.3.6.4.1 Exploratory Endpoint (Exp 3): Microbial Levels in Returned IVRs and Microbial Growth on Swabs from Returned IVRs (EVMS Only)
Completer Population

	Treatment Group		
	**TFV+LNG (N= 9)**	**TFV Alone (N= 10)**	**Placebo (N= 5)**
**STAPHYLOCOCCUS AUREUS**			
0 (NONE SEEN)	9 (100)	9 (90.0)	4 (100)
1+ (RARE)	0 (0.0)	1 (10.0)	0 (0.0)
Total	9	10	4
**UREAPLASMA**			
0 (NONE SEEN)	9 (100)	10 (100)	4 (100)
Total	9	10	4
**ANAEROBIC GRAM-NEGATIVE RODS, OTHER**			
0 (NONE SEEN)	9 (100)	10 (100)	4 (100)
Total	9	10	4
**ANAEROBIC GNR NON-PIGMENTED**			
0 (NONE SEEN)	8 (88.9)	10 (100)	4 (100)
1+ (RARE)	1 (11.1)	0 (0.0)	0 (0.0)
Total	9	10	4
**YEAST NOT ALBICANS**			
0 (NONE SEEN)	9 (100)	10 (100)	4 (100)
Total	9	10	4
